# Permanent porous hydrogen-bonded frameworks with two types of Brønsted acid sites for heterogeneous asymmetric catalysis

**DOI:** 10.1038/s41467-019-08416-6

**Published:** 2019-02-05

**Authors:** Wei Gong, Dandan Chu, Hong Jiang, Xu Chen, Yong Cui, Yan Liu

**Affiliations:** 1 School of Chemistry and Chemical Engineering and State Key Laboratory of Metal Matrix Composites, Shanghai Jiao Tong University, Shanghai, 200240 China; 2 Collaborative Innovation Center of Chemical Science and Engineering, Tianjin, 300072 China

## Abstract

The search for porous materials with strong Brønsted acid sites for challenging reactions has long been of significant interest, but it remains a formidable synthetic challenge. Here we demonstrate a cage extension strategy to construct chiral permanent porous hydrogen-bonded frameworks with strong Brønsted acid groups for heterogeneous asymmetric catalysis. We report the synthesis of two octahedral coordination cages using enantiopure 4,4’,6,6’-tetra(benzoate) ligand of 1,1’-spirobiindane-7,7’-phosphoric acid and Ni_4_/Co_4_-*p*-*tert*-butylsulfonylcalix[4]arene clusters. Intercage hydrogen-bonds and hydrophobic interactions between *tert*-butyl groups direct the hierarchical assembly of the cages into a permanent porous material. The chiral phosphoric acid-containing frameworks can be high efficient and recyclable heterogeneous Brønsted acid catalysts for asymmetric [3+2] coupling of indoles with quinone monoimine and Friedel-Crafts alkylations of indole with aryl aldimines. The afforded enantioselectivities (up to 99.9% ee) surpass those of the homogeneous counterparts and compare favorably with those of the most enantioselective homogeneous phosphoric acid catalysts reported to date.

## Abstract (web summary)

The search for porous materials with strong Brønsted acid sites for challenging chemical reactions has been of significant interest, but remains challenging. Here the authors report a cage extension strategy to construct chiral permanent porous hydrogen-bonded frameworks with strong Brønsted acid groups for heterogeneous asymmetric catalysis.

## Introduction

Porous solid materials with Brønsted acid sites (BASs) have been extensively used as environmentally friendly heterogeneous catalysts in chemical and petrochemical processes^1^. Continued efforts have been focused on the search for new solid Brønsted acid catalysts for challenging and practically important reactions^2–5^ including asymmetric reactions, which afford enantiopure and high value-added chemicals still catalyzed by homogeneous catalysts^6^. Metal-organic frameworks (MOFs) have recently been explored as asymmetric catalysts for their structural diversity and tunable porosity^7–12^, with the most efficient examples all containing organic linkers derived from privileged chiral Lewis acid catalysts^11,12^. By contrast, there are only few reports on Brønsted acidic MOFs that are catalytically active but with low enantioselectivities (6–84% ee)^13–16^. It is hard to make chiral MOFs featuring strong BASs from sulfonated or phosphonated organocatalysts because they are often the ligating functionality of choice for framework construction^7,17,18^. For example, arylphosphocarboxylates can form stronger bonds with metal ions than pure carboxylates, but they tend to form dense frameworks without BASs through both carboxylate and phosphonate groups^17,18^. Remarkably, very recently, hydrogen-bonded organic frameworks (HOFs) constructed by linking organic molecules or metal-complexes through intermolecular hydrogen bonds have become an alternative to extended framework materials^19–23^. The mild conditions typically used to synthesize HOFs allow the rational design of crystalline frameworks with reactive functional groups such as phosphonic acids through judicious choices of building blocks, thus making them interesting complements to MOFs^24–26^. In this work, we demonstrated that strong chiral BASs can be directly built in HOFs through a cage extension strategy for heterogeneous asymmetric catalysis.

Metal-organic cages are molecular containers^27–31^ and are attractive building blocks for construction of supramolecular porous materials as they provide high chemical and structural diversity and possess inherent porosity and functions^27,32–34^. Although their structures are intrinsically porous, the cage solids generally collapse upon solvent removal due to the lack of strong bonding between cages^27–34^. Hydrogen-bonding interactions between coordination cages have been observed before^35–40^. For example, Eddaoudi et al. reported two porous materials with zeolite-like topologies constructed from metal-imidazolate cubes through the vertex-to-vertex hydrogen bonding^35^. Since then, more hydrogen-bonded frameworks based on cubic metal-imidazolate cages and tetrahedral metal-carboxylate cages have been reported^36–40^, but only one of them was capable of retaining its crystalline structure upon desolvation^40^. Here we report how to address this issue through careful design of coordination cages covering with two types of acid groups capable of forming strong hydrogen bonds to another, as exemplified in the context of hierarchical construction of chiral permanent porous HOFs based on octahedral cages.

**Fig. 1:**
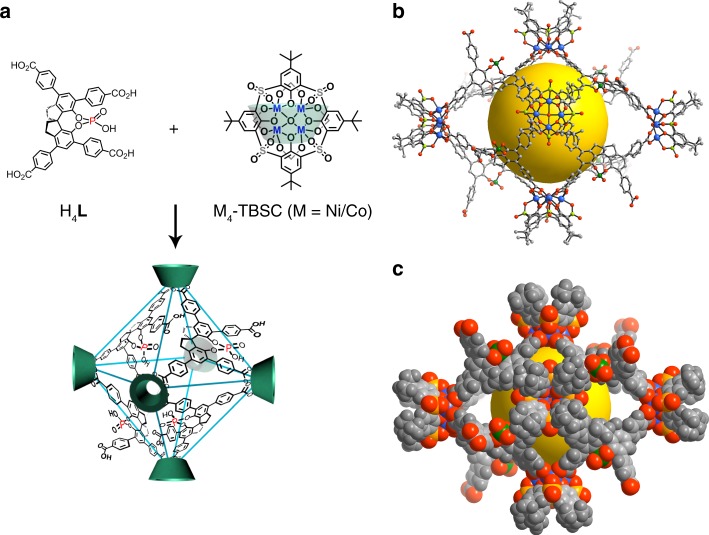
Assembly procedures. **a** Self-assembly of cages **1**-Ni and **1**-Co (only half of the H**L**^3−^ ligand are shown in the cage for clarity). **b** The single-crystal structure of the octahedral cage in **1**-Ni and **c** the space-filling model with an elliptical shape viewed along the short axis (sky-blue, Ni; green, P; yellow, S; gray, C; red, O). The cavities are highlighted by colored spheres

Chiral phosphoric acids derived from axially diols such as 1,1′-binaphthol (BINOL)^41^ and 1,1′-spirobiindane-7,7′-diol (SPINOL)^42^ have found widespread application as Brønsted acid catalysts in enantioselective transformations involving imines, such as Mannich^43^, Friedel-Crafts (F-C)^44^, Pictet-Spengler^45^, and aza-Diels-Alder reactions^46^. On the other hand, sulfonylcalix[4]arenes react with metal ions to generate a shuttlecock-like tetrametallic cluster, which is an ideal four-connected node in assembling nanosized cages by binding to auxiliary organic linkers^47,48^. Following the construction principles of coordination cages, we chose the M_4_-calixarene as the square unit and the multitopic ligand 4,4′,6,6′-tetrakis(4-benzoic acid)-1,1′-spinol phosphonate (H_4_**L**) as the potential triangular unit (Fig. 1a). Intercage hydrogen bonds formed between carboxylic and phosphoric acid groups direct the assembly of octahedral cages into a robust porous HOF. The phosphoric acid-containing solids can be highly enantioselective heterogeneous catalysts for [3+2] coupling of indoles with quinone monoimine and F-C alkylation of indole with aryl aldimines, with enantioselectivities surpassing the homogeneous counterparts and comparing favorably with the most stereoselective homogeneous phosphoric acid catalysts that have been reported so far.

## Results

### Synthesis and characterization

The ligand H_4_**L** was prepared in an overall 58% yield through four steps by utilizing enantiopure spinol^49^ as starting material. Heating a mixture of H_4_**L**, H_4_TBSC and NiCl_2_·6H_2_O or Co(NO_3_)_2_·6H_2_O (a 1:1:10 molar ratio) in DMF and water in the presence of 0.1 M HCl solution at 80 °C for 72 h afforded rectangular-shaped crystals of {[M_4_(*μ*_4_-H_2_O)(TBSC)]_6_[H**L**]_8_}·G (M = Ni and Co for **1**-Ni, **1**-Co, respectively) in good yields (Supplementary Figure 8). The formulations of them were supported by the results of IR spectroscopy (Supplementary Figure 12), microanalysis, and thermogravimetric analysis (TGA). The products were insoluble in H_2_O and common organic solvents including MeOH, DMF, and DMSO (Supplementary Figure 10). However, we were capable of isolating the discrete octahedral cages in MeOH by adding a little amount of formic acid, as evidenced by quadrupole-time-of-flight mass spectrometry (Q-TOF-MS), UV-vis and circular dichroism (CD) spectra (Supplementary Figures 6, 7 and 11). The phase purity of the bulk samples was established by comparison of their observed and simulated powder X-ray diffraction (PXRD) patterns (Fig. 4a and Supplementary Figure 14). The slight discrepancy between the calculated and experimental patterns probably result from framework distortion caused by partial loss of guest molecules of the samples after being exposed to air.

Single-crystal X-ray diffraction showed that (*S*)-**1**-Ni crystallizes in the chiral triclinic *P*1 space group, with a whole cage in the asymmertic unit. The structure has no crystallographic symmetry and is comprised of six shuttlecock-like tetranuclear Ni_4_-TBSC nodes and eight H**L**^3−^ linkers. Three of the four crystallographically independent carboxylate groups are deprotonated and coordinate to six Ni centers from three Ni_4_-TBSC clusters in a bidentate fashion, while the fourth carboxylate group remains protonated and uncoordinated to the Ni_4_-TBSC vertex (Fig. 1b and Supplementary Figure 1). Each Ni(II) center adopts an octahedral geometry coordinated by one sulfonyl and two phenoxo oxygen atoms from the deprotonated TBSC^4−^ ligand, two carboxylate oxygen atoms from two different H**L**^3−^ ligands and one *μ*_4_-H_2_O molecule. Thus, six Ni_4_-TBSC clusters as the four-connected vertexes are linked by eight tritopic H**L**^3−^ linkers as the faces to form a truncated octahedral cage with an elliptical shape. A space-filling representation of **1**-Ni clearly shows the formation of a porous cage with a substantially elliptical shape (Fig. 1c). The cage has totally eight pairs of free phosphoric and benzoic acid groups, each of which located on one of the eight faces of an octahedron. The overall size of the cage is ~4.0 × 4.0 × 5.1 nm^3^, and the size of the inner cavity is 2.1 × 2.1 × 3.2 nm^3^ (excluding Van der Waals radius). The cage featuring a large internal volume (~3339 Å^3^) contains two kinds of apertures. The large one has a diagonal distance of 1.2 ×1.9 nm^2^ and the small one has a diagonal distance of 1.0 × 1.5 nm^2^. There are many examples of discrete coordination cages with large cavities that contain functional groups on their periphery surfaces, but none of them are functionalized with carboxylic or phosphoric acid groups^29–31^.

**Fig. 2:**
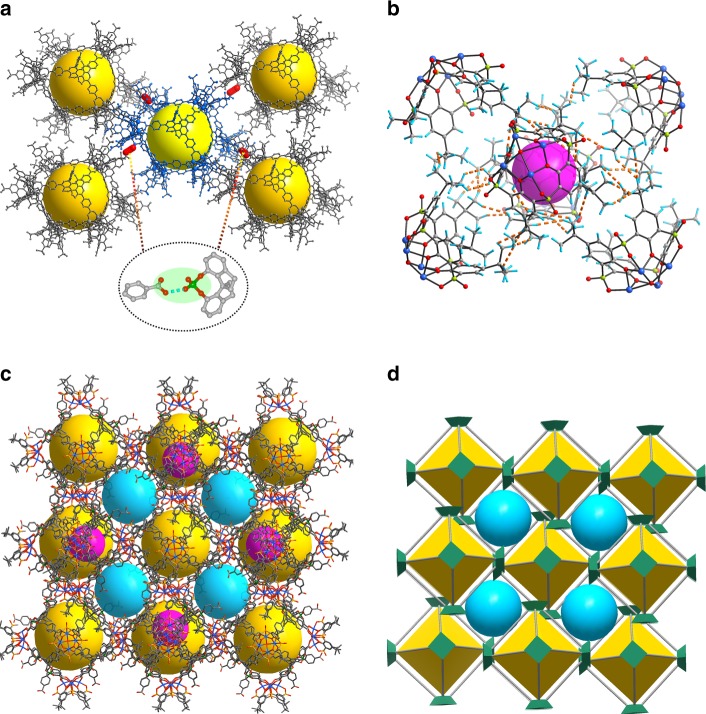
Crystal structures. **a** Four hydrogen bonds formed between five adjacent cages. **b** A hydrophobic cavity formed by six TBSC moieties from six cages. **c** The 3D HOF structure showing octahedral cages interconnected by two types of newly generated cages. **d** Simplified 3D packing structure. The cavities are highlighted by colored spheres

Each cage utilizes two pairs of carboxylic and phosphoric acid groups to form four O–H···O intermolecular hydrogen bonds with two pairs of phosphoric and carboxylic acids from four neighboring cages, with other six pairs of the organic acid groups not involved in hydrogen bonding (Fig. 2a and Supplementary Figures 2 and 3). The short O···O distances of 2.608(1) and 2.695(1) Å are indicative of strong hydrogen-bonding interactions^50^. Meanwhile, each TBSC unit in the cage interacts with five neighboring TBSC units from five different cages forming complex intercluster π–π, CH···π, CH···O, and C–H···H–C interactions (Fig. 2b). As a result, two kinds of extrinsic octahedral cages with a maximum diameter of 1.5 and 0.36 nm, respectively, are generated via the arrangement of six TBSC units of six cages and eight triangular faces of eight cages, as shown in Fig. 2c and Supplementary Figure 4. Intercluster interactions thus direct the assembly of molecular octahedral cages into an extended 3D porous supramolecular framework functionalized with phosphoric and benzoic acid groups (Fig. 2d).

**1**-Co is isostructural to **1**-Ni and adopts a similar porous cage structure that is built from Co_4_-TBSC clusters and H**L**^3−^ linkers with an inner cavity size of 2.1 × 2.1 × 3.2 nm^3^ (excluding Van der Waals radius). The cage **1**-Co also features eight pairs of carboxylic and phosphoric acid groups, directed outside the cage, thus serving as a building block which extends to a 3D polymer. Calculations using PLATON show that both **1**-Co and **1**-Ni have about ∼70% of the total volume available for guest inclusion^51^. These volumes are presumably filled with solvent molecules such as DMF and/or H_2_O, which are unfortunately highly disordered and could not be located by X-ray crystallography. It should be noted that the chiral phosphoric acid groups of the H**L**^3−^ ligands are exposed to the interstitial pores accessible to guest molecules. Porous solids of this type may offer great opportunities for studies of supramolecular chemistry and host-guest interactions, and the present two cages are attractive examples of supramolecular assemblies decorated with chiral functional groups that have been characterized by single-crystal X-ray crystallography^52–57^. To the best of our knowledge, they represent the first two examples of crystalline porous frameworks with channels decorated with two types of BASs^58,59^. In addition, we were also capable of preparing single crystals of racemic **1**-Ni and **1**-Co from the racemic ligand H_4_**L** under similar reaction conditions. X-ray diffraction revealed each of them adopts a porous hydrogen-bonded 3D structure that is built from racemic [M_4_(*μ*_4_-H_2_O)(TBSC)]_6_[H**L**]_8_}cages (the result will be reported in the near future). To evaluate the generality of our synthetic approach to HOFs, we synthesized a new 4,4′,6,6′-tetra(1-naphthoate) ligand of 1,1′-spirobiindane-7,7′-phosphoric acid (H_4_**L**′), as shown in Supplementary Figure 5. As expected, heating a DMF/H_2_O solution containing H_4_**L**′, H_4_TBSC, and Co(NO_3_)_2_·6H_2_O afforded a similar octahedral cage **1**-Co', as revealed by single-crystal X-ray diffraction (Supplementary Figure 5). Strong intercluster hydrogen bonding and supramolecular interactions direct the packing of the cages into a 3D porous HOF. So, the present synthetic strategy may hold great promise for preparing more cage-based expanded structures. Further work on related porous materials is ongoing in this lab.

**Fig. 3:**
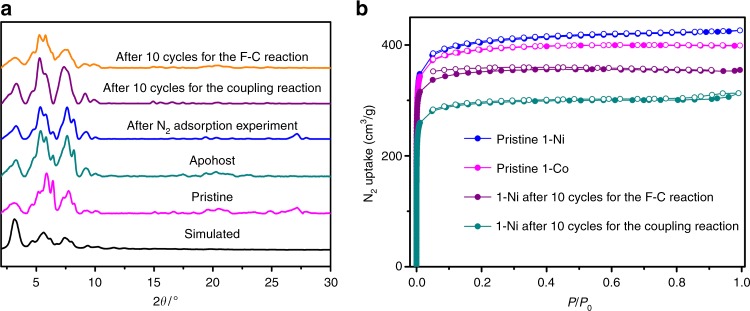
PXRD and N_2_ adsorption/desoption spectra. **a** PXRD patterns of **1**-Ni. **b** N_2_ adsorption (filled symbols) and desorption (open symbols) isotherms at 77 K

Circular dichroism (CD) spectra of **1**-Ni and **1**-Co made from *R* and *S* enantiomers of the H_4_**L** ligand are mirror images of each other, indicative of their enantiomeric nature (Supplementary Figure 11). TGA revealed that the guest molecules could be readily removed in the temperature range from 50 to 150 °C and the frameworks started to decompose at about 420 °C (Supplementary Figure 9). Removal of the guest molecules led to structural distortion of the framework, thereby displaying some differences in relative peak intensities in the PXRD patterns for the evacuated and pristine samples (Fig. 3a). Their permanent porosity was demonstrated by N_2_ adsorption measurements at 77 K. After desolvation of the n-hexane-exchanged samples, both **1**-Ni and **1**-Co exhibit a type I sorption behavior, with a Brunauer–Emmett–Teller (BET) surface area of 1239 and 1192 m^2^ g^−1^, respectively (Fig. 3b).

### Heterogeneous asymmetric catalysis

The permanent porosity and regularly distributed phosphoric acid sites of the solids prompted us to explore their utilization as heterogeneous catalysts. In each cage, two of eight phosphoric moieties are engaged in H-bonding interactions, and the remaining six phosphoric acids are available for catalysis. [3+2] coupling of 3-substituted indoles with quinone monoimine catalyzed by enantiopure Brønsted acid catalysts offers direct access to optically active benzofuroindoline derivatives that are commonly found in a diverse array of important natural alkaloids^60^. So, our initial reaction was carried out with 3-methyl indole and 4-methyl-*N*-(4-oxocyclohexa-2,5-dienylidene)benzenesulfonamide. After screening a variety of reaction conditions including solvent, temperature and catalyst loading (Supplementary Table 5), we found that, in the presence of 0.04 mol% loading of **1**-Ni (0.04 μmol cage), the reaction proceeded smoothly to give the benzofuroindoline in 92% yield and 99.9% ee within 14 h (Table 1, entry 1 and Supplementary Figure 15). Further lowering the catalyst loading to 0.02 mol% led to a decrease in both the yield and ee value (Supplementary Table 5, entry 12).

**Table 1:** Asymmetric [3+2] coupling of 3-substituted indoles with quinone monoimine^a^


Entry	Cat.	R_1_	R_2_	R_3_	Yield (%)^b^	ee (%)^c^
1	**1**-Ni	Me	H	H	92	99.9 (*S*)
2		Me	Me	H	90	95 (*S*)
3		Me	H	5-Me	92	94 (*S*)
4		Me	H	6-F	90	95 (*S*)
5		Me	H	6-Me	95	99.9 (*S*)
6		Me	H	5-OMe	86	91 (*S*)
7		Me	H	5-Br	83	92 (*S*)
8^d^		Phenyl	H	H	65	99.7 (*S*)
9^d^		Benzyl	H	H	37	94 (*S*)
10^d^		PIME^e^	H	H	9	n.d.
11		Me	H	H	94	99.9 (*R*)^f^
12	**1**-Co	Me	Me	H	92	94 (*S*)
13		Me	H	5-Me	89	93 (*S*)
14		Me	H	6-F	88	93 (*S*)
15	PhCO_2_H^g^	Me	H	H	n.d.	n.d.
16	**1**-Ni*/Me_4_**L**^h^	Me	H	H	94 (79)	89 (78) (*S*)
17		Me	H	5-Me	90 (77)	87 (78) (*S*)
18		Me	H	6-F	93 (76)	89 (75) (*S*)
19		Me	H	5-Br	91 (72)	87 (76) (*S*)
20		Phenyl	H	H	70 (73)	91 (80) (*S*)
21		Benzyl	H	H	69 (72)	90 (87) (*S*)
22		PIME^e^	H	H	56 (64)	n.d. (n.d.)

^a^Reaction conditions: 3-substituted indole (0.10 mmol), quinone monoimine (0.15 mmol), and (*S*)-**1**-Ni (0.04 mol% catalyst based on indole) in CH_3_CN (1 mL), 0 °C, 14 h

^b^Isolated yield

^c^Determined by HPLC

^d^Reaction time: 48 h

^e^PIME = phthalimidoethyl (size: 6.5  ×  15.2  Å^2^, Supplementary Figure 13)

^f^The *R* enantiomer was produced using (*R*)-**1**-Ni as the catalyst

^g^Catalyzed by 0.24 mol% of benzoic acid

^h^Catalyzed by 0.03 mol% of the discrete cage **1**-Ni* and 0.24 mol% of (*S*)-Me_4_**L**

With the optimal reaction conditions in hand, we next examined the substrate scope with respect to various 3-substituted indoles. As shown in Table 1 (entries 2–7), the electronic nature or positions of the substituent on the indole appeared to have limited effects on the catalytic results, and the benzofuroindolines were afforded in 83–95% yields and 91–99% ee. The absolute configurations of the products were established to be *S* by the comparison of their HPLC peaks with the reported results^60^. The chiral nature of the product is dominated by the handedness of the catalyst, as evidenced by that reaction catalyzed by (*R*)-**1**-Ni gave the *R* enantiomer over the *S* enantiomer (Table 1, entry 11). We also examined the catalytic activity of **1**-Co for the coupling reactions. Under identical conditions, the reactions afforded the products with almost the same yields and enantioselectivities as those obtained with **1**-Ni (Table 1, entries 12–14), indicating that the M_4_(*μ*_4_-H_2_O)(TBSC) nodes in the cages have no obvious effect on the catalytic performance. In addition, when benzoic acid was used as catalyst, no coupling reaction of 3-methyl indole and quinone monoimine was observed (Table 1, entry 15), indicating the reaction was catalyzed by the immobilized phosphoric acids. We also performed the coupling reaction involving deprotonation of **1**-Ni by adding a little amount of Et_3_N or NH_3_·H_2_O, but did not detect any targeted product even after 72 h, further indicating the catalysis emanates from the phosphoric acid sites.

**Fig. 4:**
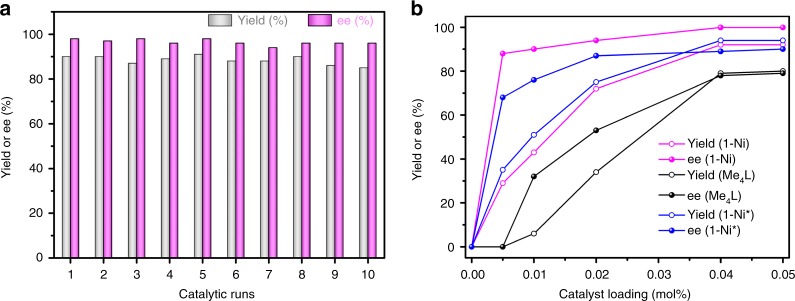
Recycle and confinement effect. **a** Recycling tests of the HOF **1**-Ni catalyst in the [3+2] coupling reaction of 3-methyl indole with quinone monoamine. **b** Plots of the [3+2] coupling reaction of 3-methyl indole with quinone monoimine at different catalyst loadings (catalyst loadings of Me_4_**L** and **1**-Ni* are 6 and 0.75 times of HOF **1**-Ni, respectively)

Multiple experiments were conducted to study the heterogeneity and recyclability of the solid catalyst. First, by using 3-methyl indole as substrate, removal of **1**-Ni by filtration after 2 h completely shut down the reaction, affording only 45% total conversion upon stirring for 14 h. This indicates that there exists no active species in the reaction solution. Second, upon completion of the reaction, the catalyst could be readily recovered from the catalytic reaction via centrifugation, and used repeatedly with negligible deterioration in yields and enantioselectivities for the following 10 runs (Fig. 4a). After ten cycles, the recovered catalyst retained crystallinity and porosity, as indicated by the PXRD and BET surface area (1069 m^2^ g^−1^) (Fig. 3). Third, inductively coupled plasma mass spectrometric (ICP-MS) analysis of the product solution showed that <0.003% of the nickel had leached into the solvent per cycle, either as molecular species or as particles too small to be removed by filtration through Celite.

To ascertain that the substrates access the internal phosphoric acid sites via the open channels, we have examined three indole derivatives of different sizes. Molecular mechanics simulations indicated that these indoles have estimated sizes ranging from 6.5 × 11.5 Å^2^ to 6.5 × 15.2 Å^2^ (Supplementary Figure 16). As expected, the substrate sizes have no obvious effects on the efficiency for the discrete cage **1**-Ni* or Me_4_**L** catalyzed [3+2] coupling reactions (Table 1, entries 20–22). In contrast, the yields of the reaction products catalyzed by **1**-Ni greatly depend on the substituent size: as the size of the indoles increases, the yield of the final product steadily decreases (65, 37, and 9%; Table 1, entries 8–10). Specially, only a 9% yield of the desired product was observed for the very bulky reactant 3-(2-phthalimidoethyl)-indole, which was much lower than 56% and 64% yields obtained with **1**-Ni* and Me_4_**L**, respectively, presumably because it cannot access the catalytic sites in the cavity through the windows (10.5 × 14 Å^2^) due to its large diameters (6.5 × 15.2 Å^2^). This finding indicated that the catalytic reaction may mainly occur in the pores of the solid catalyst.

To further study the confinement effect of the porous structure on phosphoric acids, the catalytic performance of the homogeneous controls **1**-Ni* and Me_4_**L** were tested. Under otherwise identical conditions, with the equal loading of phosphoric acid sites, both **1**-Ni* and Me_4_**L** can catalyze [3+2] coupling of 3-methyl indole with 4-methyl-N-(4-oxocyclohexa-2,5-dienylidene) benzenesulfonamide affording 94% yield with 89% ee and 79% yield with 78% ee of the products, respectively (Table 1, entry 16). Therefore, the HOF **1**-Ni displayed higher enantioselectivity (99.9% ee) than the homogeneous controls **1**-Ni* and Me_4_**L** under otherwise identical reaction conditions. Similar behaviors were observed for other substrates (Table 1, entries 17–19). The increased ee values may arise from geometrical constraints imposed by the chiral micro-environments around the phosphoric acids, which may restrict molecular motion to improve enantiodiscrimination. As shown in Fig. 4b, the difference in catalytic performances of **1**-Ni, 1-Ni*, and Me_4_**L** became large at a low catalyst loading. Specially, when the loading was decreased from 0.04 to 0.02 mol%, **1**-Ni can give 72% yield with 94% ee of the product, while the homogeneous controls **1**-Ni* and Me_4_**L** only gave 75% yield with 87% ee and 34% yield with 53% ee, respectively. Continuing to decrease the loading to 0.01 mol%, **1**-Ni also can afford 43% yield with 90% ee, which obviously surpass the results catalyzed by **1**-Ni* and Me_4_**L** (51% yield with 76% ee and 6% yield with 32% ee, respectively). When the catalyst loading reach to a very small magnitude of 0.005 mol%, the molecular catalyst Me_4_**L** nearly lost its activity, however, the discrete cage **1**-Ni* and the HOF catalyst **1**-Ni still can achieve 35% yield with 68% ee and 29% yield with 88% ee, respectively. The catalytic performance became poor on lowing the loading of Me_4_**L**, probably due to conversion of catalytic sites into less active and selective species in dilute solutions. So, confinement of catalytically active Brønsted acids in the cage, especially the cage-based solid can stabilize the organocatalyst and even enhance the catalyst activity and stereoselectivity at a very low catalyst loading. It should be noted that increasing the catalysts loading from 0.04 to 0.05 mol% cannot obviously increase yield and enantioselectivity (80% yield with 80% ee for Me_4_**L**, 94% yield with 90% ee for **1**-Ni*, and 92% yield with 99.9% ee for **1**-Ni).

**1**-Ni can also catalyze the asymmetric F-C alkylations of indole and aryl aldimines, which provides a completely atom-economical access to enantiopure 3-indolylmethanamine derivatives^44,61^, key components of many biologically active natural and natural-like products. Under optimized conditions (Supplementary Table 6), the reaction of N-tosylimine with indole catalyzed by 0.04 mol% loading of (*R*)-**1**-Ni gave the product in 92% isolated yield and 91% ee in DCE at 0 °C after 10 h (Table 2, entry 1). A variety of aryl-substituted aldimines containing electron-donating and electron-withdrawing groups on the aromatic rings were tolerated, affording 89–99% isolated yields with 90–99.7% ee. The highest enantioselectivity was attained with *o*-methyl-substituted arylimine, affording 89% yield with 99.7% ee. Again, homogeneous controls **1**-Ni* and Me_4_**L** (0.03 mol% and 0.24 mol% loadings, respectively) exhibited lower enantioselectivities than the HOF **1**-Ni, further illustrating the superiority of the polycage catalyst. Several tests also proved the heterogeneous nature of **1**-Ni in the above F-C reactions. The heterogeneous catalyst can be recycled and reused for at least 10 runs with negligible loss of efficiency and enantioselectivity as well, and the recovered solid retained crystallinity and porosity and remained structurally intact (Fig. 3, Supplementary Table 8).

**Table 2:** Asymmetric Friedel-Crafts reaction of *N*-sulfonyl aldimines with indole^a^


Entry	Cat.	R	Yield (%)^b^	ee (%)^c^
1	(*R*)-**1**-Ni	4-H	92	91 (*R*)
2		4-Br	95	97 (*R*)
3		4-F	96	94 (*R*)
4		4-Cl	96	98 (*R*)
5		4-CF_3_	93	99 (*R*)
6		4-Me	89	90 (*R*)
7		2-NO_2_	99	97 (*R*)
8		2-Me	89	99.7 (*R*)
9		3-Br	92	99.6 (*R*)
10	(*R*)-**1**-Ni*/Me_4_**L**^d^	4-H	87 (93)	88 (86) (*R*)
11		4-Br	90 (95)	95 (95) (*R*)
12		4-Me	80 (92)	88 (86) (*R*)

^a^Reaction conditions: *N*-sulfonyl aldimine (0.1 mmol), indole (0.3 mmol), and (*R)*-**1**-Ni (0.04 mol% catalyst based on *N*-sulfonyl aldimine) in DCE (1 mL), 0 °C, 10 h

^b^Isolated yield

^c^Determined by HPLC

^d^Catalyzed by 0.03 mol% of the discrete (*R*)-**1**-Ni and 0.24 mol% of (*R*)-Me_4_**L**

Considerable efforts have been made on immobilizing chiral phosphoric acids on solid supports such as silica, linear and dendrimer-like polymers and amorphous porous organic polymers for high recyclability despite the fact that the resulting catalysts generally suffer from the disadvantages of uneven catalytic sites and low catalyst loading^62–67^(Supplementary Table 7). With few exceptions^63^, the examined catalysts typically provide unsatisfactory selectivity. A pair of chiral MOFs has also been prepared based on enantiopure binol-phosphoric acids and investigated as heterogeneous Brønsted acid catalysts for F-C alkylation of indole with imines^14^, but with much lower activity (20–45% yield) and stereoselectivity (6–44% ee) than the HOF **1**-Ni. To the best of our knowledge, the stereoselectivity of **1**-Ni exceeded those of reported heterogeneous catalysts for the F-C reactions^68^ and were comparable even to those of the most enantioselective homogeneous systems based on phosphoric acids reported to date^44,61^. On the other hand, only few chiral phosphoric acids have been reported to be highly enantioselective homogeneous catalysts for [3+2] coupling of indoles with quinone monoimine and no heterogeneous catalysts have been reported for such coupling reactions. The 3,3′-bis(2,4,6-triisopropylphenyl)-binol phosphoric acid catalyst was shown to afford the best result, with 86–99% ee for the asymmetric [3+2] coupling reactions, which are comparable to the results of the HOF catalyst **1-**Ni^60^. Therefore, high stereoselectivities are noteworthy features of the present **1**-Ni-based protocol, which are even among the highest enantioselective values reported for homogeneous organocatalysts^41,60,61^.

## Discussion

We have reported two chiral spinol-based octahedral cages covered with uncoordinated phosphoric and carboxylic acid groups, allowing for cages to self-assemble into permanent porous HOFs through preordered hydrogen bonds. The HOF frameworks possess channels decorated with free chiral phosphoric acid groups can be heterogeneous and recyclable Brønsted acid catalysts to promote [3+2] coupling of indoles with quinone monoimine and F-C alkylations of indole and aryl aldimines with high enantioselectivities (up to 99.9% ee). The HOF catalyst can be recovered and recycled at least ten times without loss of activity and enantioselectivity. This work thus not only provides a new approach to prepare highly enantioselective heterogeneous Brønsted acid catalysts but also lays a solid foundation for the development of novel chiral permanent porous materials from metallocages and metallacycles for enantioslective processes such as enantioselective recognition, sensing and separation, asymmetric catalysis, and chiral optics.

## Methods

### Synthesis of HOFs **1**-Ni and **1**-Co

A mixture of NiCl_2_·6H_2_O (142.6 mg, 0.6 mmol) or Co(NO_3_)_2_·6H_2_O (174.6 mg, 0.6 mmol), H_4_TBSC (51 mg, 0.06 mmol), and H_4_**L** (47.7 mg, 0.06 mmol) was ultrasonic dissolved in DMF/H_2_O (10 mL/1 mL). 1.2 mL 0.1 M HCl was added and the mixture was heated at 80 °C for 3 days. Light green crystals of **1**-Ni (75.5 mg, 78%) or burgundy crystals of **1**-Co (81.4 mg, 84%) were collected, washed with DMF and acetone, and dried in air. FTIR (KBr, cm^−1^) for **1**-Ni: 3440 (m), 2964 (w), 1711 (w), 1606 (s), 1497 (s), 1459 (w), 1400 (s), 1262 (s), 1194 (w), 1136 (w), 1085 (s), 1018 (m), 900 (w), 838 (s), 795 (s), 627 (w), 568 (s), 497 (m). **1**-Co: 3429 (m), 2968 (w), 1722 (w), 1612 (s), 1502 (s), 1450 (w), 1436 (s), 1259 (s), 1189 (w), 1140 (w), 1046 (s), 1022 (m), 896 (w), 843 (s), 789 (s), 622 (w), 567 (s), 493 (m). Elemental Analysis for **1**-Ni: Calcd for C_600_ H_472_ Ni_24_ O_174_ P_8_ S_24_: C, 55.85; H, 3.66; Found: C, 56.72; H, 3.21. **1**-Co: Calcd for C_600_ H_472_ Co_24_ O_174_ P_8_ S_24_: C, 55.76; H, 3.46; Found: C, 56.44; H, 3.38.

### Synthesis of HOF **1**-Co′

Synthesis of **1**-Co′: A mixture of Co(NO_3_)_2_·6H_2_O (174.6 mg, 0.6 mmol), H_4_TBSC (51 mg, 0.06 mmol), and H_4_**L**′ (59.7 mg, 0.06 mmol) was ultrasonic dissolved in 10 mL DMF, 2 mL 0.1 M HCl was added and the mixture was heated at 80 °C for 3 days. Square light red crystals were collected and subjected to X-ray test. FTIR (KBr, cm^−1^): 3350 (m), 2960 (w), 1654 (w), 1606 (s), 1594 (s), 1467 (m), 1371 (s), 1263 (s), 1201 (w), 1131 (w), 1084 (s), 1018 (m), 906 (w), 838 (s), 797 (s), 624 (w), 564 (s). Elemental Analysis: Calcd for C_728_ H_536_ Co_24_ O_178_ P_8_ S_24_: C, 59.99; H, 3.68; Found: C, 56.52; H, 3.40.

### Synthesis of discrete cage **1**-Ni*

Hundred milligram fresh HOF **1**-Ni were immersed into 1 mL MeOH and 10 μL anhydrous formic acid was added. After ultrasonic treatment for a while (about 5 min), the crystals of HOF **1**-Ni were dissolved and gave a clear solution. Then, Et_2_O were added and the solution turned cloudy immediately. The discret **1**-Ni* were collected by centrifugation and dried in air (81 mg). **1**-Ni* was characterized by quadrupole-time-of-flight mass spectrometry (Q-TOF-MS), UV-vis, and circular dichroism (CD) spectra etc.

### General procedure for asymmetric [3+2] coupling reaction

To a flame-dried Schlenk Pressure Tube was added **1**-Ni or **1**-Co (0.52 mg, 0.04 μmol), 3-substituted indoles (0.1 mmol) and 0.5 mL anhydrous MeCN, and the mixture was stirred at 0 °C under N_2_ atmosphere for 30 min. Quinone monoimine (0.15 mmol) in 0.5 mL precooled anhydrous MeCN was then added via syringe. The mixture was stirred at 0 °C until the reaction completed. Chromatography on silica gel (1:2, EA-PE, v/v) afforded the desired products.

### General procedure for asymmetric Friedel-Crafts reaction

To a flame-dried Schlenk Pressure Tube was added **1**-Ni (0.52 mg, 0.04 μmol), indole (0.3 mmol) and 0.5 mL anhydrous DCE, and the mixture was stirred at 0 °C under N_2_ atmosphere for 30 min. N-sulfonyl aldimines (0.1 mmol) in 0.5 mL precooled anhydrous DCE was then added via syringe. The mixture was stirred at 0 °C until the reaction completed. Chromatography on silica gel (1:3, PE-EA, v/v) afforded the desired products.

### Single-crystal X-ray crystallography

Single-crystal XRD data for **1**-Ni and **1**-Co were collected on a Bruker SMART Apex II CCD-based X-ray diffractometer with Cu-Kα radiation in the Instrumental Analysis Center of Shanghai Jiao Tong University, China. Single-crystal XRD data for **1**-Co′ were collected on Beamline BL17B of National Facility for Protein Science at Shanghai Synchrotron Radiation Facility (SSRF). The empirical absorption correction was applied by using the SADABS program^69^. The structures were solved using direct method, and refined by full-matrix least-squares on *F*^2^ by the SHELXTL-2014 software package. All non-H atoms were refined anisotropically. DFIX, SADI, FLAT, DANG, EADP, and SIMU restrains were used to obtain reasonable parameters due to the poor quality of crystal data. All the phenyl rings were constrained to ideal six-membered rings. The solvent molecules were highly disordered, and attempts to locate and refine the solvent peaks were unsuccessful. Contributions to scattering due to these solvent molecules were removed using the SQUEEZE routine of PLATON, structures were then refined again using the data generated under *OLEX2-1.2*^70^. The contents of the solvent region are not represented in the unit cell contents in the crystal data. Crystal data and details of the data collection are given in Supplementary Table 1, while the selected bond distances and angles are presented in Supplementary Tables 2–4.

## Supplementary information

Supplementary Information

## Data Availability

The X-ray crystallographic coordinates for the structures reported in this article have been deposited at the Cambridge Crystallographic Data Centre (CCDC), under deposition numbers CCDC 1823162, 1823163, and 1880376. These data can be obtained free of charge from The Cambridge Crystallographic Data Centre via www.ccdc.cam.ac.uk/data_request/cif. All other data supporting the findings of this study are available within the article and its Supplementary Information, or from the corresponding author upon reasonable request.
