# Supplementary material for: Permanent porous hydrogen-bonded frameworks with two types of Brønsted acid sites for heterogeneous asymmetric catalysis
Source: Nat Commun. 2019 Feb 5;10:600. doi: 10.1038/s41467-019-08416-6 (PMC6363736; doi:10.1038/s41467-019-08416-6)
Supplement: Supplementary file 1 — Supplementary Information [file 41467_2019_8416_MOESM1_ESM.pdf]

Supporting Information for

Permanent Porous Hydrogen-Bonded Frameworks with Two Types of Brønsted Acid Sites for Heterogeneous Asymmetric Catalysis

Gong et al.

Supplementary Methods

Materials and general procedures. All the chemicals are commercial available, and used without further purification. Elemental analyses of C and H were performed with an EA1110 CHNS-0 CE elemental analyzer. The IR (KBr pellet) spectra were recorded (400-4000 cm^{-1} region) on a Nicolet Magna 750 FT-IR spectrometer. The samples for solid-state CD spectroscopy were prepared by mixing the crystals with KBr to get homodispersed powders after grinding. Then, the powders were subjected to a punching machine to get uniform discs. The discs were then directly subjected to J-800 spectropolarimeter (Jasco, Japan). Thermogravimetric analyses (TGA) were carried out in an N_2 atmosphere with a heating rate of 10 ℃/min on a STA449C integration thermal analyzer. Powder X-ray diffraction (PXRD) data were collected on a Bruker D8 Advance diffractometer using Cu Kα radiation. The calculated PXRD patterns were produced using the SHELXTL-XPOW program and single crystal reflection data. ^{1}H NMR ,^{13}C NMR and ^{31}P NMR experiments were carried out on a MERCURY plus 400 spectrometer operating at resonance frequencies of 400 MHz. ESI-MS were recorded on a Finnigan LCQ mass spectrometer using dichloromethane-methanol as mobile phase. Analytical high performance liquid chromatography (HPLC) was performed on a Shimadzu 2010A with UV detection. Analytical CHIRALCEL OD-H columns (4.6 mm×25 cm), CHIRALCEL AD-H columns (4.6 mm×25 cm) from Daicel were used. N_2 adsorption isotherms were measured using a Micrometritics ASAP 2020 surface area analyzer at 77 K with a liquid N_2 bath. Before the adsorption measurement, all the samples were washed with DMF 3 times, and then first exchanged with CH_2Cl_2 3 times over 1 hour (20 minutes each) and then immersed in n-hexane over 1 hour replacing the solvent every 20 minutes. The as-treated samples were degassed on ASAP 2020 for 10 h at room temperature.

Synthesis of Ligands

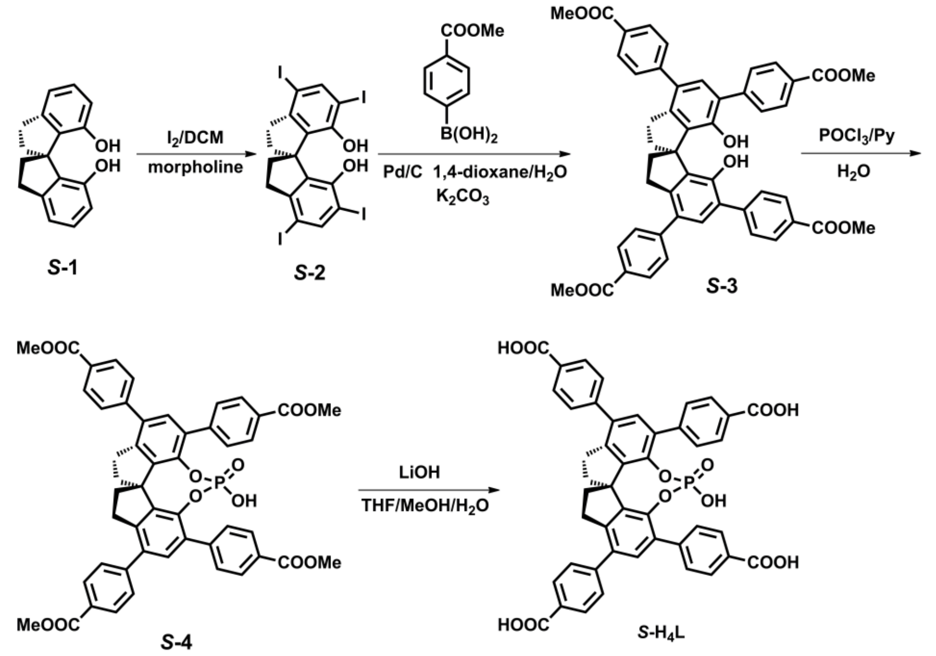

Synthesis of *S*-2. ***S*-1** was synthesized according to the reported literature.[1,2] To a solution of *S*-1 (2 g, 7.93 mmol) in DCM (250 mL) was added morpholine (8.98 g, 103 mmol) and I_2 (14.1 g, 55.51 mmol). The mixture was stirred at room temperature for 12 h. After that, 250 mL water was

added. The organic phase was washed with diluent HCl and saturated $Na_2S_2O_3$, dried over anhydrous Na_2SO_4, and concentrated under reduced pressure. The resulted crude product was purified by column chromatography on silica gel (1:5, DCM-PE, v/v) to yield *S*-2 as a white solid (5.4 g, 90%). ^{1}H NMR (400 MHz, $CDCl_3$) δ 7.85 (s, 2H), 5.08 (s, 2H), 3.03 – 2.88 (m, 4H), 2.32 (dt, *J* = 12.8, 9.2 Hz, 2H), 2.26 – 2.14 (m, 2H). ^{13}C NMR (101 MHz, $CDCl_3$) δ: 151.43, 150.53, 144.41, 133.75, 84.82, 83.49, 63.69, 37.07, 36.71.

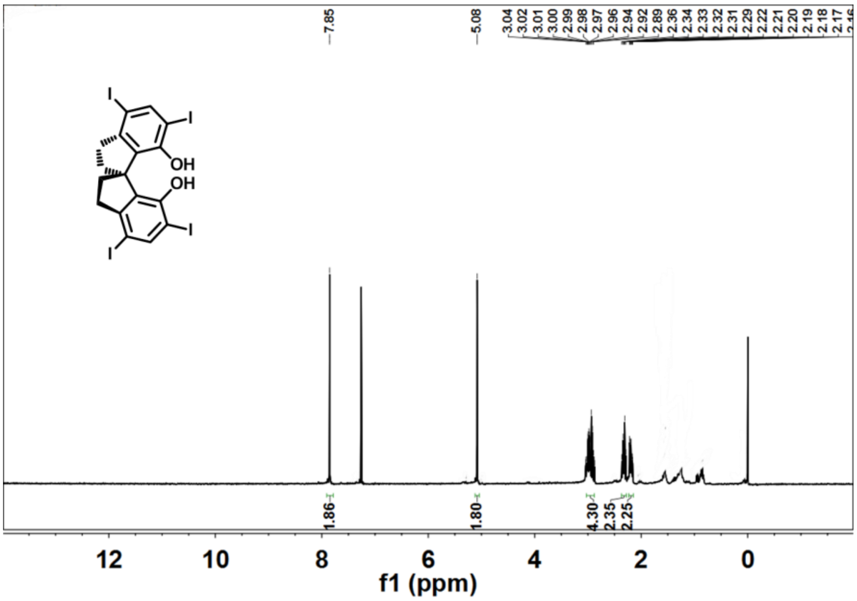

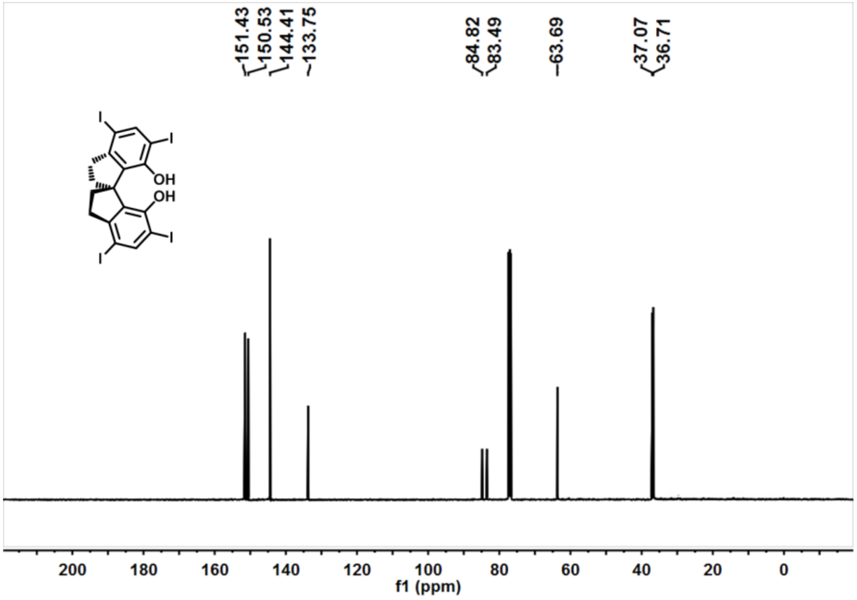

Synthesis of *S*-3. *S*-2 (2.5 g, 3.3 mmol), 4-(methoxycarbonyl)benzeneboronic acid (4.75 g, 26.4 mmol), K_2CO_3 (4.56 g, 33 mmol) and 10% Pd/C (0.7 g, 0.66 mmol) were added into a 250 mL flame-dried round-bottom flask. The flask was evacuated under vacuum and refilled with N_2 for three times. Degassed dioxane (40 mL) and water (40 mL) were added, and the mixture was heated at 85 ^{o}C for 4 h. Then the reaction mixture was cooled to room temperature and concentrated. The residue was extracted with DCM. The organic layer was collected, dried over anhydrous Na_2SO_4, and concentrated under reduced pressure. The crude product was purified by column chromatography on silica gel (3:1, PE-EA, v/v) to yield *S*-3 as a white solid (1.88 g, 75%).

^{1}H NMR (400 MHz, $CDCl_3$) δ 8.09 (dd, *J* = 12.6, 8.1 Hz, 4H), 7.57 (dd, *J* = 20.5, 8.1 Hz, 4H), 7.31 (s, 1H), 5.18 (s, 1H), 3.94 (s, 3H), 3.91 (s, 3H), 3.30 – 3.19 (m, 1H), 3.10 (dd, *J* = 16.5, 7.9 Hz, 1H), 2.45 (ddd, *J* = 32.1, 17.8, 10.0 Hz, 2H). ^{13}C NMR (101 MHz, CDCl3) δ: 166.99, 166.81, 149.44, 144.96, 143.59, 141.75, 132.27, 131.27, 130.83, 129.89, 129.73, 129.28, 129.09, 128.55, 128.47, 126.98, 58.72, 52.19, 37.41, 31.18.

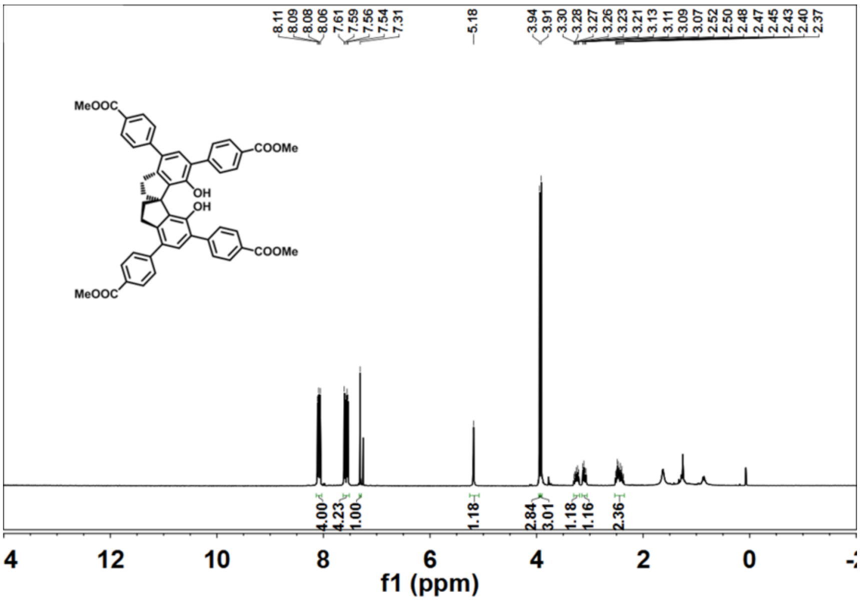

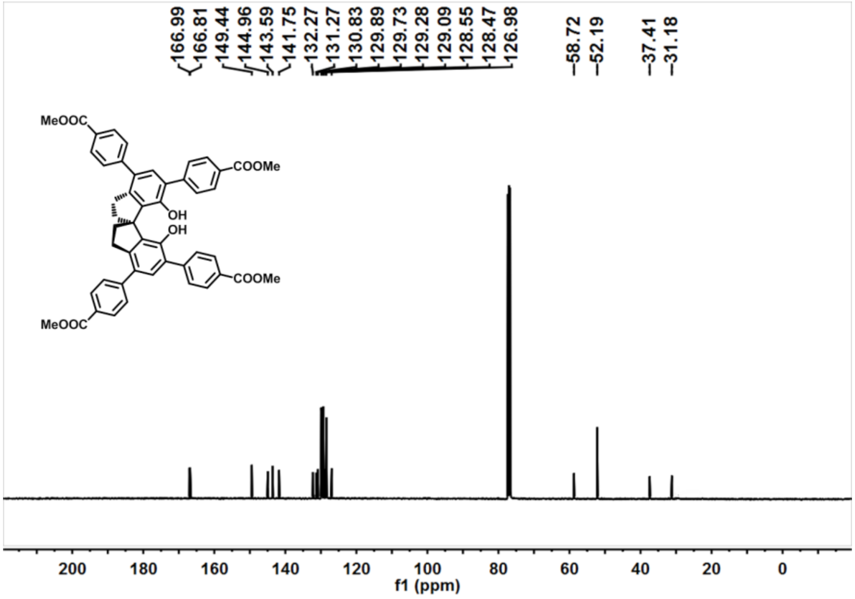

Synthesis of *S*-4. To a solution of *S*-3 (1.88 g, 2.38 mmol) in anhydrous pyridine (20 mL) was added $POCl_3$ (730 mg, 4.76 mmol) slowly at 0 °C. After stirring at 70 ℃ for 6 h, the reaction mixture was quenched by addition of distilled water (5 mL) slowly at 0 ℃ and then stirred at 110 ℃ for 12 h. After cooling to room temperature, the mixture was acidified to pH=5 with 6 M HCl and then extracted with DCM. The organic layer was washed with brine, dried over Na_2SO_4, and concentrated. The crude product was purified by column chromatography on silica gel (1:10, MeOH-DCM, v/v) to yield *S*-4 as a white solid (1.83 g, 90%). ^{1}H NMR (400 MHz, $CDCl_3$) δ: 8.12 (d, J = 6.8 Hz, 4H), 7.88 (d, J = 7.8 Hz, 4H), 7.56 (d, J = 5.7 Hz, 9H), 7.34 (s, 2H), 3.96 (s, 6H), 3.75 (s, 6H), 3.24 (dd, J = 14.6, 9.5 Hz, 2H), 2.91 (dd, J = 14.6, 6.8 Hz, 2H), 2.46 (dd, J = 10.9,

4.0 Hz, 2H), 2.34-2.24 (m, 2H).^{13}C NMR (101 MHz, CDCl3) δ: 167.35, 166.92, 144.42, 143.87, 143.59, 142.52, 142.25, 141.35, 141.07, 135.57, 134.47, 130.52, 129.70, 129.54, 129.02, 128.57, 60.39, 52.26, 38.44, 30.26. ^{31}P NMR (162 MHz, $CDCl_3$) δ: -9.80.

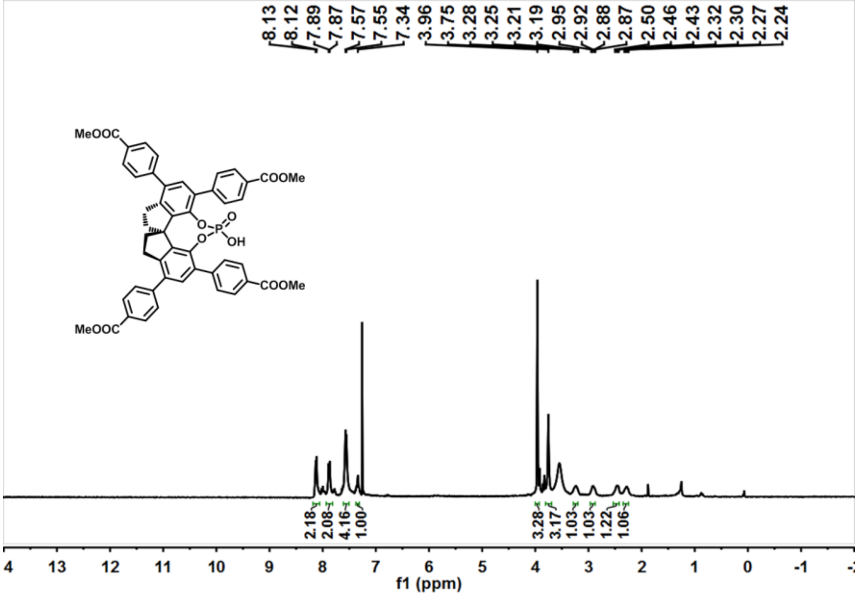

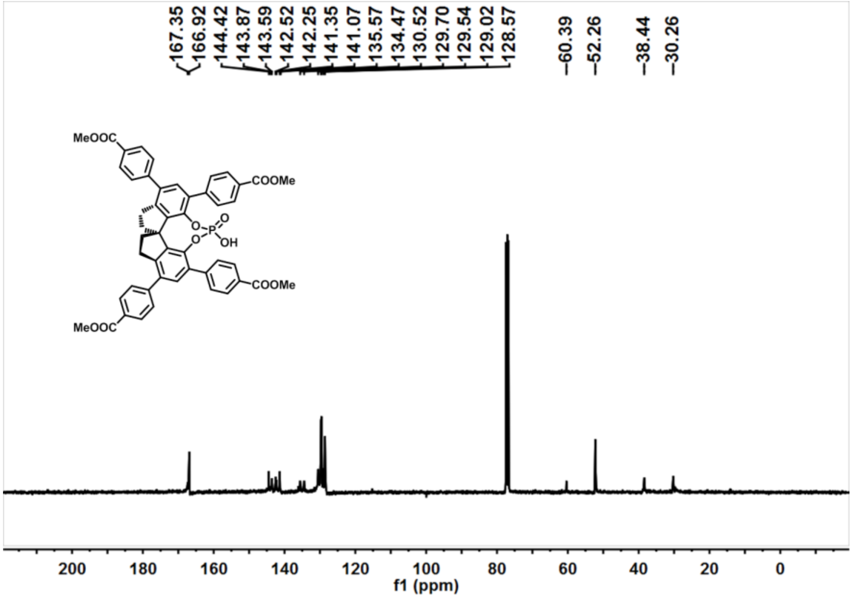

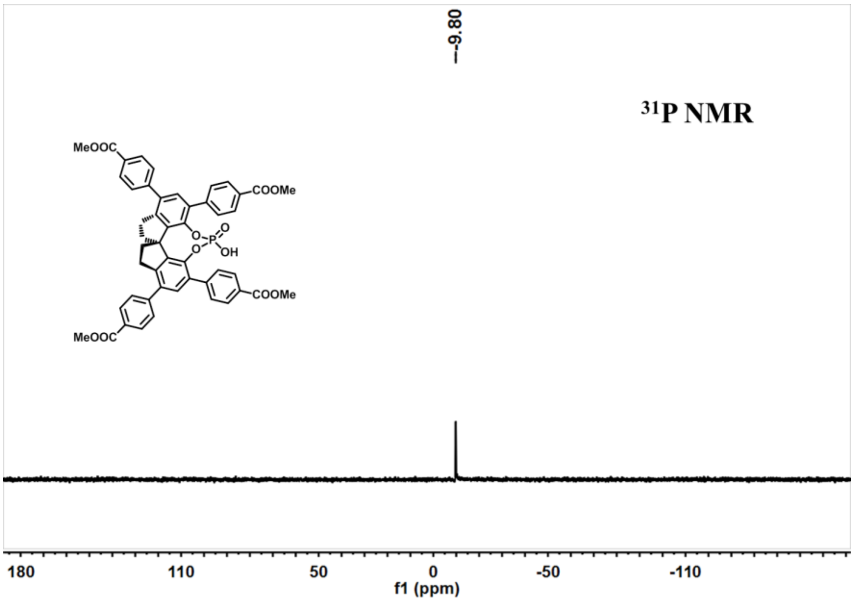

Synthesis of *S*-H_4L. A solution of *S*-4 (1.83 g, 2.15 mmol) and LiOH H_2O (3.62 g, 86 mmol) in THF (20 mL), MeOH (60 mL) and H_2O (60 mL) was heated at 70 ℃ for 12 h. After cooling to room temperature, the organic solvents were removed *in vacuo* and the water phase was acidified to pH = 2 with 3 M HCl. The precipitate was filtrated and dried under vacuum at 60 ℃ overnight. *S*-H_4**L** was obtained as a white solid (1.63 g, 95%). ^{1}H NMR (400 MHz, DMSO-d_6) δ: 12.88 (s, 4H), 8.02 (d, J = 8.2 Hz, 4H), 7.85 (d, J = 8.2 Hz, 4H), 7.72 (t, J = 7.0 Hz, 8H), 7.28 (s, 2H), 2.77 (dd, J = 16.2, 8.2 Hz, 4H), 2.41 (dd, J = 10.9, 5.9 Hz, 2H), 2.02 (dd, J = 20.0, 9.9 Hz, 2H). ^{13}C NMR (101 MHz, DMSO-d_6) δ: 167.94, 167.69, 145.80, 145.71, 144.77, 143.73, 142.94, 134.75, 133.09, 129.99, 129.75, 129.30, 129.11, 99.98, 60.02, 30.27. ^{31}P NMR (162 MHz, DMSO-d_6) δ: -11.73. ESI-MS: m/z 792.8235 (Calcd m/z 793.1475 for [H_4**L** - H]$^-$). FTIR (KBr, cm^{-1}): 3386 (s), 1697 (s), 1607 (s), 1396 (m), 1244 (s), 1179 (m), 1094 (m), 1021 (m), 979 (w), 856 (m), 781 (w), 719 (w), 546 (w).

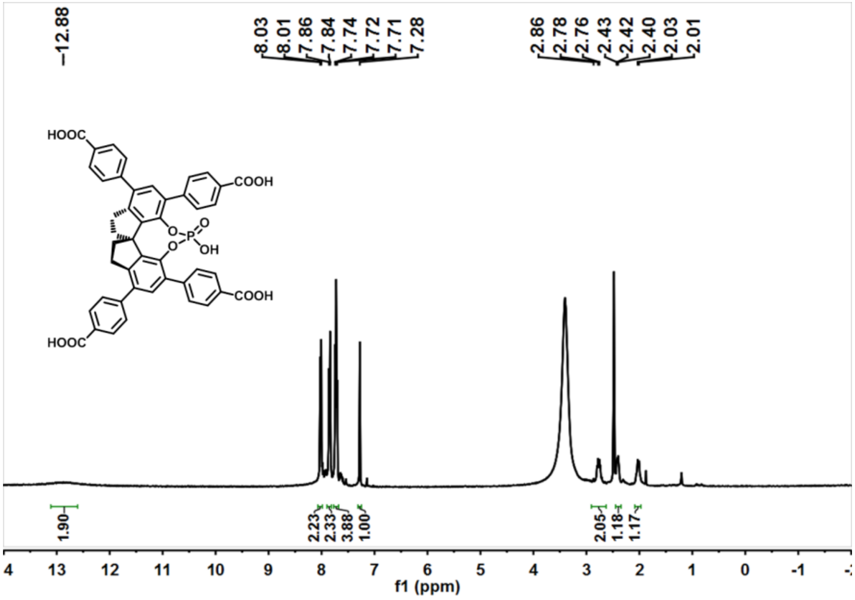

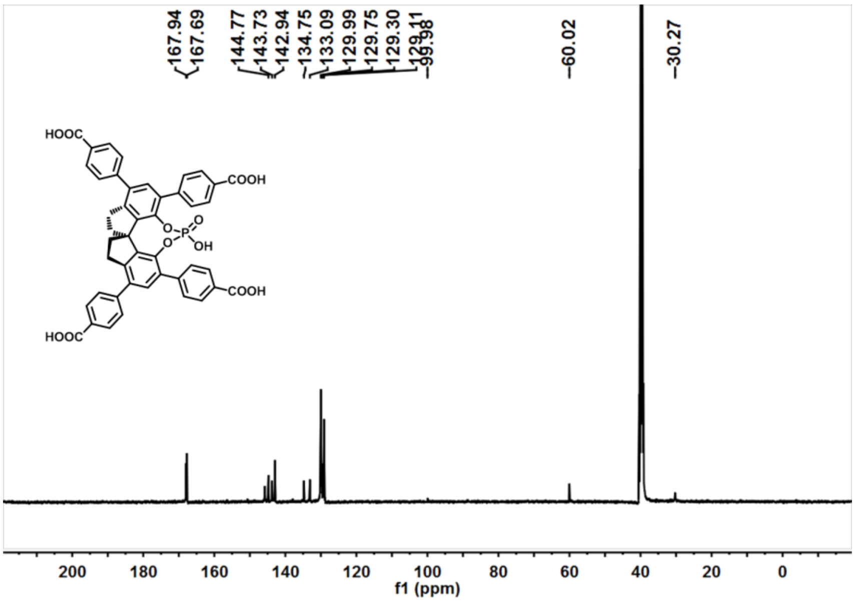
167.94
167.69
144.77
143.73
142.94
134.75
133.09
129.99
129.75
129.30
129.11
99.98
60.02
30.27
HOOC
COOH
O
P
OH
f1 (ppm)

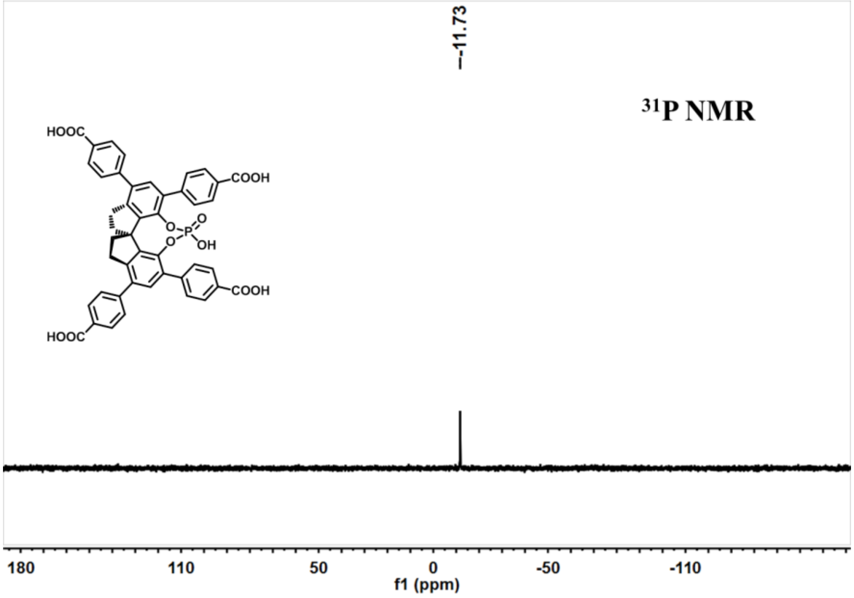
-11.73
31P NMR
HOOC
COOH
O
P
OH
f1 (ppm)

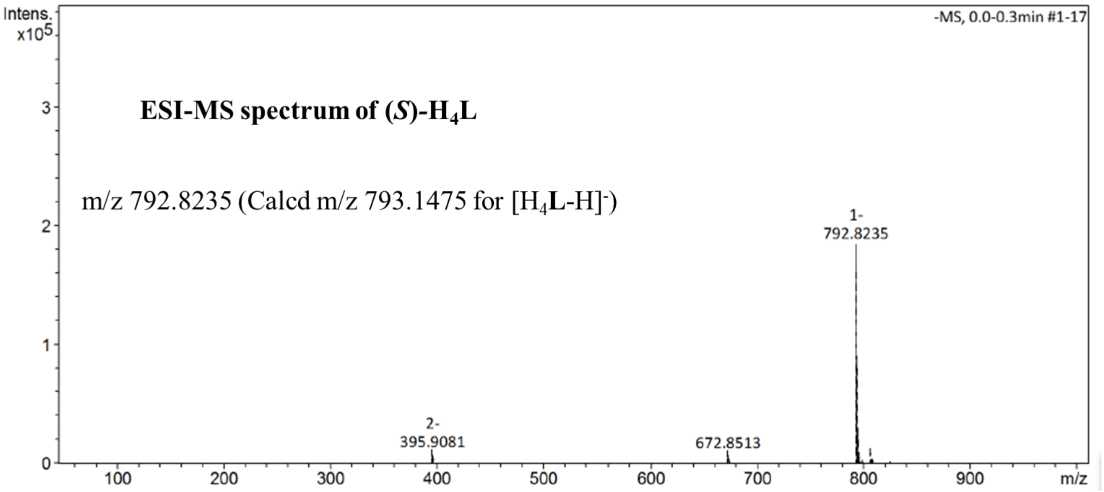
Intens.
x10^5
-MS, 0.0-0.3min #1-17
ESI-MS spectrum of (S)-H4L
m/z 792.8235 (Calcd m/z 793.1475 for [H4L-H]-)
1-
792.8235
2-
395.9081
672.8513
m/z

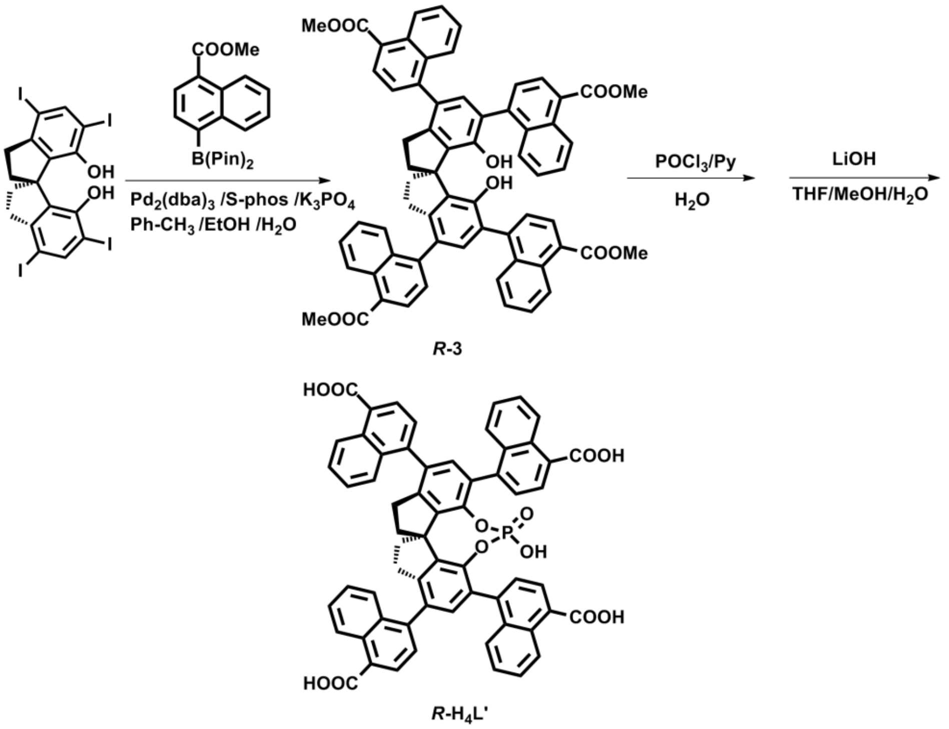

Synthesis of *R*-3. *R*-2 (1.5 g, 1.98 mmol), 4-(methoxycarbobyl) naphthalene-1-boronic acid pinacol ester (4.94 g, 15.84 mmol), K_3PO_4 (8.4 g, 39.6 mmol), $Pd_2(dba)_3$ (0.35 g, 0.396 mmol) and S-phos (0.35g, 0.792 mmol) were added into a 250 mL flame-dried round-bottom flask. The flask was evacuated under vacuum and refilled with N_2 for three times. Degassed toluene (75 mL), EtOH (25 mL) and water (25 mL) were added, and the mixture was heated at 95 ^{o}C for 24 h. Then the reaction mixture was cooled to room temperature and concentrated. The residue was extracted with DCM. The organic layer was collected, dried over anhydrous Na_2SO_4, and concentrated under reduced pressure. The crude product was purified by column chromatography on silica gel (4:1:1, PE-EA-DCM, v/v) to yield *R*-3 as a white solid (1.2 g, 62%). ^{1}H NMR (500 MHz, $CDCl_3$) δ 9.12 – 8.94 (m, 2H), 8.36 – 8.16 (m, 2H), 7.90 – 7.38 (m, 8H), 7.27 – 7.16 (m, 1H), 5.35 – 5.01 (m, 1H), 4.11 – 4.00 (m, 6H), 3.08 – 2.67 (m, 2H), 2.65 – 2.42 (m, 2H). ^{13}C NMR (126 MHz, $CDCl_3$) δ 171.13, 168.07, 167.92, 149.64, 145.15, 143.62, 140.38, 137.29, 133.92, 132.22, 131.91, 131.63, 129.93, 129.58, 128.83, 128.45, 127.87, 127.55, 126.50, 84.19, 60.44, 59.34, 52.25, 41.38, 36.08, 34.71, 31.63, 30.94, 29.10, 24.94, 21.07, 14.18, 11.49.

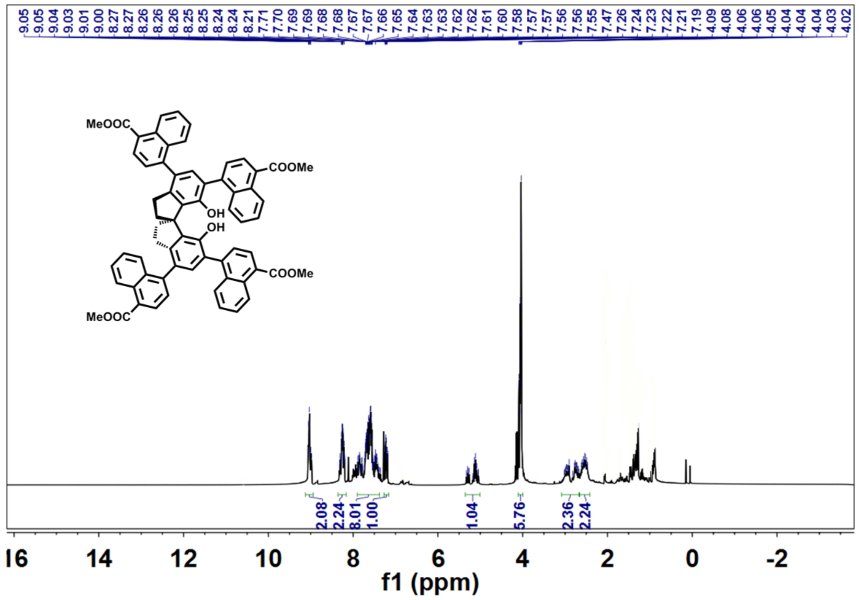

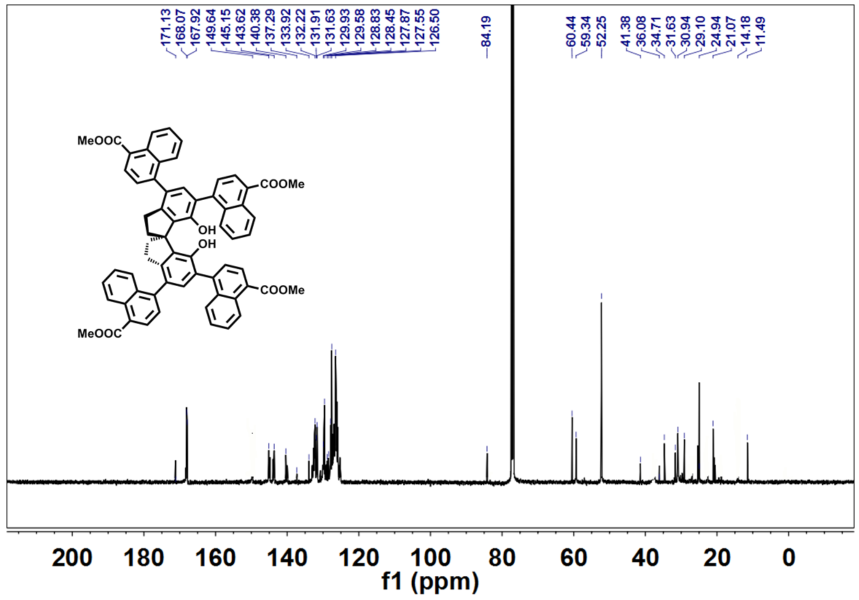

Synthesis of *R*-H$_4$L'. To a solution of *R*-3 (1.2 g, 1.21 mmol) in anhydrous pyridine (15 mL) was added $POCl_3$ (650 mg, 4.24 mmol) slowly at 0 ^{o}C. After stirring at 70 ℃ for 6 h, dioxane (10 mL) was added and the reaction mixture was quenched by addition of distilled water (4 mL) slowly at 0 ℃ and then stirred at 110 ℃ for 12 h. After cooling to room temperature, the mixture was acidified to pH=5 with 6 M HCl and then extracted with DCM. The organic layer was washed with brine, dried over Na_2SO_4, and concentrated (without further purification) to get the crude product. Then, LiOH H_2O (2.74 g, 65 mmol), THF (20 mL), MeOH (60 mL) and H_2O (60 mL) were added and heated at 70 ℃ for 12 h. After cooling to room temperature, the organic solvents were removed *in vacuo* and the water phase was acidified to pH = 2 with 3 M HCl. The precipitate was filtrated and dried under vacuum at 60 ℃ overnight. *R*-H$_4$**L'** was obtained as an off-white solid (0.95 g, 84%). ^{1}H NMR (500 MHz, DMSO) δ 8.90 (dd, *J* = 41.1, 34.9 Hz, 2H), 8.30 – 8.02 (m, 2H), 7.72 (ddd, *J* = 77.8, 76.5, 17.3 Hz, 8H), 7.15 – 6.98 (m, 1H), 3.17 (s, 2H), 2.26 (s, 2H). ^{13}C NMR (101 MHz, DMSO) δ 169.73, 169.70, 169.66, 146.24, 143.96, 142.84, 141.95, 133.53, 133.20, 132.97, 132.17, 132.06, 131.86, 131.49, 130.87, 129.55, 128.86, 128.74, 127.55, 126.83,

60.32, 60.18, 59.83, 36.29, 31.66, 31.23, 29.87, 29.37, 29.03, 22.49, 14.27. ^{31}P NMR (162 MHz, DMSO-d_6) δ: -11.22. Q-TOF-MS: m/z 1017.2373 (Calcd m/z 1017.2179 for [H_4**L'**+Na]$^+$). FTIR (KBr, cm^{-1}): 3396 (s), 1692 (s), 1579 (s), 1427 (m), 1241 (s), 1201 (m), 1089 (m), 1058 (m), 959 (w), 841 (m), 776 (s), 717 (w), 558 (w).

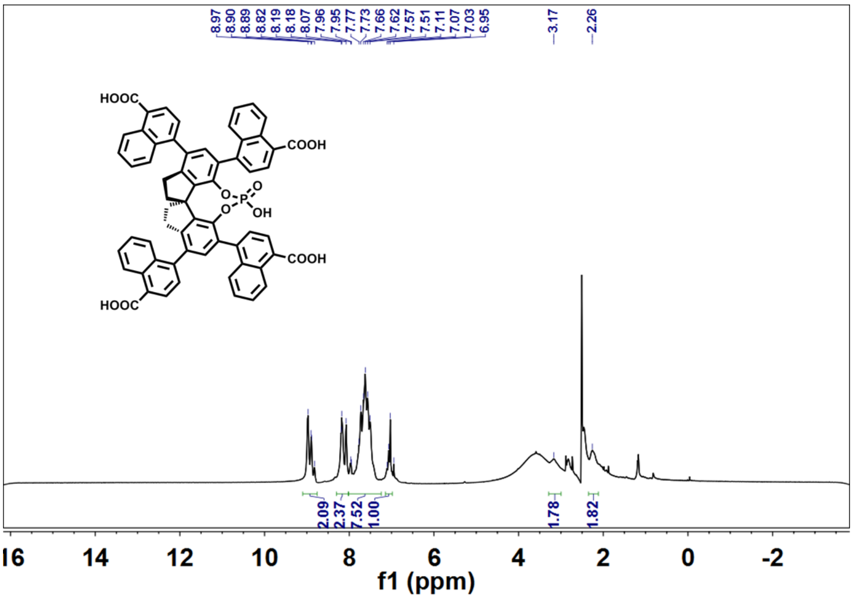

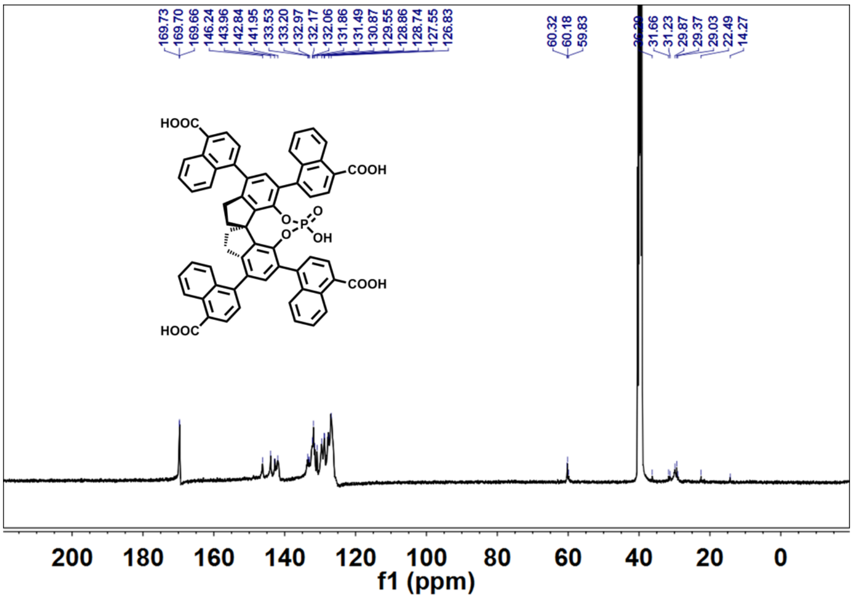

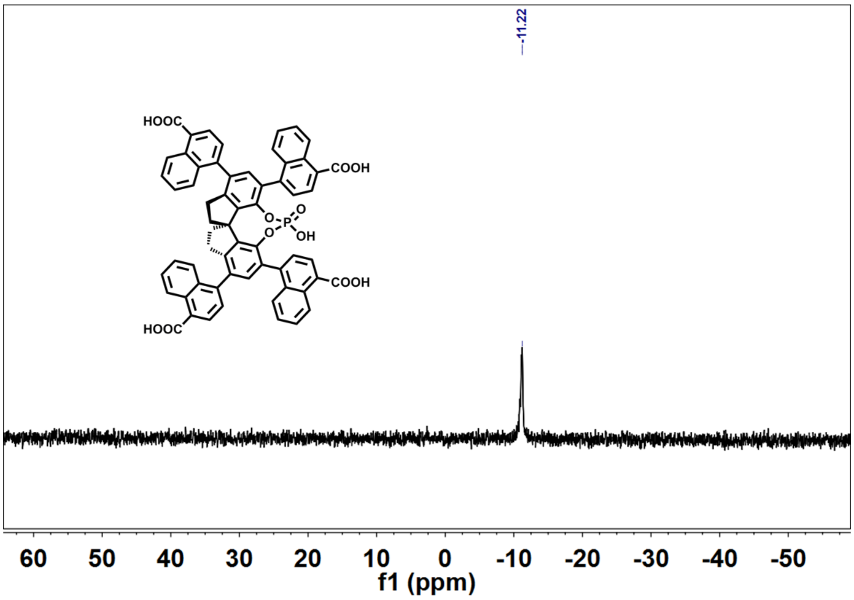

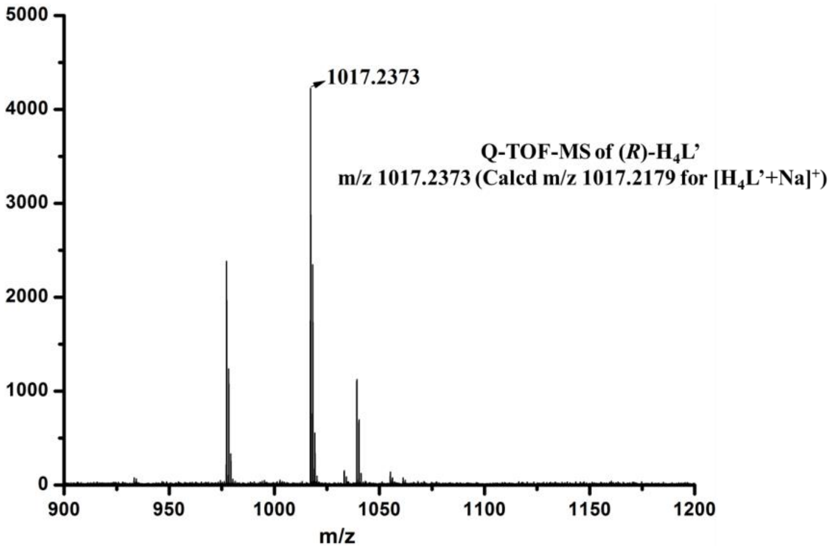

Substrate synthesis

The substrates 4-methyl-N-(4-oxocyclohexa-2,5-dienylidene)benzenesulfonamid, 3,5-dimethyl indole, 3-methyl-6-fluorine indole, 3-methyl-5-bromine indole, 3-methyl-5-methoxyl indole, 3,6-dimethyl indole, 3-phthalimidoethyl indole and 3-phenyl indole and 3-benzyl indole were prepared according to reported procedures. [3-7]

All N-Ts aryl aldimines were prepared according to reported procedures.[8]

Supplementary Tables

Supplementary Table 1. Crystal data and structure refinement for 1-Ni, 1-Co and 1-Co’

Identification code	**1**-Ni	**1**-Co	**1**-Co’
Empirical formula	C_{600} H_{472} Ni_{24} O_{174} P_8 S_{24}	C_{600} H_{472} Co_{24} O_{174} P_8 S_{24}	C_{728} H_{536} Co_{24} O_{178} P_8 S_{24}
Formula weight	12891.99	12897.27	14563.06
Temperature (K)	173.0	173.0	173.0
Wavelength (Å)	1.54178	1.54178	0.71073
Crystal system	Triclinic	Triclinic	Tetragonal
Space group	*P*1	*P*1	*I*4
Unit cell dimensions	*a* = 33.9737(14) Å *α*= 92.736(3) º *b* = 35.8121(15) Å *β*= 114.065(2) º *c* = 38.6607(16) Å *γ* =117.330(2) º	*a* = 33.883(7) Å *α*= 92.88 (3) º *b* = 35.698(7) Å *β*= 113.94(3) º *c* = 38.432(8) Å *γ* = 117.23(3) º	*a* = 33.009(5) Å *b* = 33.009(5) Å *c* = 64.894(13) Å *α*=*β*=*γ* =90 º
Volume ($Å^3$), *Z*	36519(3), 1	36149 (17), 1	70708(25), 2
Density (calculated) (mg/m^3)	0.586	0.592	0.684
Absorption coefficient (mm^{-1})	1.018	0.346	0.359
F(000)	6640	6616	14960
Reflections collected / unique	269387 / 87995	181095/ 181095	29397/ 29397
Completeness to theta	67.679, 98.1%	25.242, 94.5%	24.431, 99.6%
R_{int}	0.0690	0.061	0.133
Refinement method	Full-matrix	Full-matrix	Full-matrix
Data / restraints / parameters	87995 / 18179 / 6631	181095/ 17434 / 6619	29397 / 5514 / 1819
Goodness-of-fit on F^2	1.069	0.929	1.192
Final *R* indices [I>2sigma(I)]	R_1 = 0.1040, wR_2 = 0.2887	R_1 = 0.0621, wR_2 = 0.1693	R_1 = 0.1180, wR_2 = 0.3327
R indices (all data)	R_1 = 0.1302, wR_2 = 0.3107	R_1 = 0.0770, wR_2 = 0.1784	R_1 = 0.1388, wR_2 = 0.3457
Absolute structure parameter	0.165(13)	0.070(4)	0.03(2)
Largest diff. peak and hole (e.$Å^{-3}$)	2.132 and -0.615	0.935 and -1.182	0.628 and -1.608

Supplementary Table 2. Selected bond lengths [Å] and angles [°] for 1-Ni

Ni(2)-O(109)	1.989(15)	Ni(24)-O(51)	2.065(14)
Ni(2)-O(106)	1.971(16)	Ni(24)-O(50)	2.037(17)
Ni(2)-O(118)	2.221(13)	Ni(24)-O(58)	2.066(18)
Ni(17)-O(128)	1.985(14)	Ni(24)-O(49)	2.037(19)
Ni(17)-O(127)	2.122(16)	Ni(24)-O(40)	2.071(16)
Ni(17)-O(124)	2.100(16)	Ni(24)-O(56)	2.159(14)
Ni(17)-O(138)	1.996(17)	Ni(21)-O(51)	2.019(14)
Ni(17)-O(136)	1.946(16)	Ni(21)-O(44)	1.954(18)
Ni(17)-O(135)	2.050(15)	Ni(21)-O(42)	2.063(17)
Ni(19)-O(130)	2.113(17)	Ni(21)-O(57)	2.063(17)
Ni(19)-O(129)	2.070(14)	Ni(21)-O(43)	2.097(16)
Ni(19)-O(131)	2.061(14)	Ni(21)-O(56)	2.206(15)
Ni(19)-O(141)	1.935(18)	Ni(22)-O(54)	2.072(16)
Ni(19)-O(142)	2.025(16)	Ni(22)-O(55)	2.102(14)
Ni(19)-O(135)	2.197(15)	Ni(22)-O(43)	2.014(16)
Ni(8)-O(165)	2.001(17)	Ni(22)-O(45)	1.998(16)
Ni(8)-O(163)	2.113(16)	Ni(22)-O(56)	2.218(14)
Ni(8)-O(166)	2.202(15)	Ni(22)-O(46)	1.913(19)
Ni(8)-O(168)	1.973(17)	Ni(23)-O(54)	2.025(15)
Ni(8)-O(161)	2.155(17)	Ni(23)-O(50)	2.142(16)
Ni(8)-O(171)	1.956(17)	Ni(23)-O(48)	2.008(18)
Ni(18)-O(128)	1.992(15)	Ni(23)-O(38)	2.069(18)
Ni(18)-O(129)	2.063(15)	Ni(23)-O(56)	2.118(15)
Ni(18)-O(139)	1.912(17)	Ni(23)-O(47)	1.979(19)
Ni(18)-O(135)	2.270(15)	Ni(3)-O(117)	2.056(15)
Ni(18)-O(140)	1.960(17)	Ni(3)-O(103)	2.009(17)
Ni(18)-O(134)	2.068(17)	Ni(3)-O(102)	1.964(15)
Ni(20)-O(130)	2.041(15)	Ni(3)-O(118)	2.231(14)
Ni(20)-O(143)	1.973(16)	Ni(3)-O(113)	2.098(16)
Ni(20)-O(127)	2.091(14)	Ni(1)-O(117)	2.066(16)
Ni(20)-O(137)	1.959(15)	Ni(1)-O(116)	2.063(14)
Ni(20)-O(126)	2.032(18)	Ni(1)-O(101)	1.948(15)
Ni(20)-O(135)	2.176(16)	Ni(1)-O(114)	2.048(16)
Ni(7)-O(165)	1.979(15)	Ni(1)-O(118)	2.168(14)
Ni(7)-O(172)	1.935(18)	Ni(1)-O(100)	1.990(18)
Ni(7)-O(164)	2.050(18)	Ni(4)-O(108)	2.072(15)
Ni(7)-O(173)	2.050(17)	Ni(4)-O(105)	2.035(16)
Ni(7)-O(166)	2.067(14)	Ni(4)-O(109)	2.122(14)
Ni(7)-O(159)	2.068(17)	Ni(4)-O(118)	2.141(14)
Ni(5)-O(162)	2.102(16)	Ni(2)-O(111)	2.107(16)
Ni(5)-O(163)	2.001(15)	Ni(2)-O(99)	2.068(18)
Ni(5)-O(169)	1.998(15)	Ni(2)-O(116)	2.078(15)
Ni(5)-O(155)	2.067(17)	Ni(5)-O(167)	1.945(19)

O(117)-Ni(3)-O(118)	82.8(6)	O(49)-Ni(24)-O(50)	92.8(7)
O(117)-Ni(3)-O(113)	89.0(6)	O(49)-Ni(24)-O(58)	84.2(7)
O(103)-Ni(3)-O(117)	176.7(7)	O(40)-Ni(24)-Ni(23)	132.8(5)
O(103)-Ni(3)-O(118)	100.1(6)	O(40)-Ni(24)-O(56)	169.7(6)
O(103)-Ni(3)-O(113)	88.4(7)	O(56)-Ni(24)-Ni(21)	47.8(4)
O(102)-Ni(3)-O(117)	91.9(6)	O(51)-Ni(21)-Ni(22)	107.6(5)
O(102)-Ni(3)-O(103)	86.1(7)	O(51)-Ni(21)-O(42)	87.8(6)
O(102)-Ni(3)-O(118)	101.5(6)	O(51)-Ni(21)-O(57)	92.2(6)
O(102)-Ni(3)-O(113)	86.5(6)	O(51)-Ni(21)-O(43)	90.4(6)
O(113)-Ni(3)-O(118)	168.6(6)	O(44)-Ni(21)-O(42)	87.5(7)
O(117)-Ni(1)-O(118)	84.1(6)	O(42)-Ni(21)-Ni(24)	127.9(5)
O(116)-Ni(1)-O(117)	91.2(6)	O(42)-Ni(21)-O(43)	89.4(6)
O(116)-Ni(1)-O(118)	85.9(5)	O(42)-Ni(21)-O(56)	167.1(6)
O(101)-Ni(1)-O(117)	93.1(6)	O(57)-Ni(21)-O(43)	177.3(7)
O(101)-Ni(1)-O(116)	173.3(6)	O(57)-Ni(21)-O(56)	98.3(6)
O(101)-Ni(1)-O(114)	89.6(6)	O(43)-Ni(21)-Ni(24)	105.8(4)
O(101)-Ni(1)-O(118)	99.6(6)	O(43)-Ni(21)-Ni(22)	43.2(4)
O(101)-Ni(1)-O(100)	82.7(7)	O(43)-Ni(21)-O(56)	82.4(6)
O(114)-Ni(1)-O(117)	87.8(6)	Ni(21)-Ni(22)-Ni(23)	89.43(18)
O(114)-Ni(1)-O(116)	85.4(6)	O(54)-Ni(22)-Ni(21)	105.2(5)
O(114)-Ni(1)-O(118)	168.1(6)	O(54)-Ni(22)-Ni(23)	43.0(4)
O(100)-Ni(1)-O(117)	170.0(7)	O(55)-Ni(22)-O(56)	166.5(5)
O(100)-Ni(1)-O(116)	92.2(6)	O(43)-Ni(22)-Ni(21)	45.5(5)
O(100)-Ni(1)-O(114)	83.0(7)	O(43)-Ni(22)-Ni(23)	105.1(5)
O(100)-Ni(1)-O(118)	105.5(6)	O(43)-Ni(22)-O(54)	88.1(6)
O(8AA)-Ni(4)-O(108)	87.0(6)	O(45)-Ni(22)-Ni(23)	141.8(5)
O(8AA)-Ni(4)-O(105)	173.0(7)	O(45)-Ni(22)-O(54)	171.4(7)
O(8AA)-Ni(4)-O(109)	91.5(6)	O(45)-Ni(22)-O(55)	84.8(6)
O(8AA)-Ni(4)-O(118)	84.4(6)	O(45)-Ni(22)-O(43)	95.9(7)
O(8AA)-Ni(4)-O(104)	92.9(6)	O(45)-Ni(22)-O(56)	107.6(6)
O(108)-Ni(4)-O(109)	89.6(6)	O(46)-Ni(22)-Ni(21)	137.7(6)
O(108)-Ni(4)-O(118)	167.6(6)	O(46)-Ni(22)-Ni(23)	77.9(6)
O(105)-Ni(4)-O(108)	87.7(6)	O(46)-Ni(22)-O(43)	176.1(8)
O(105)-Ni(4)-O(109)	93.0(6)	O(46)-Ni(22)-O(45)	83.0(7)
O(105)-Ni(4)-O(118)	101.6(6)	O(46)-Ni(22)-O(56)	99.9(7)
O(105)-Ni(4)-O(104)	82.4(6)	Ni(24)-Ni(23)-Ni(22)	90.65(18)
O(109)-Ni(4)-O(118)	81.8(5)	O(54)-Ni(23)-O(50)	89.2(6)
O(104)-Ni(4)-O(108)	88.8(6)	O(48)-Ni(23)-Ni(24)	76.0(5)
O(104)-Ni(4)-O(109)	175.2(7)	O(48)-Ni(23)-Ni(22)	136.8(5)
O(104)-Ni(4)-O(118)	100.5(6)	O(48)-Ni(23)-O(54)	177.2(8)
O(111)-Ni(2)-O(118)	168.7(6)	O(38)-Ni(23)-Ni(24)	127.5(5)
O(99)-Ni(2)-O(111)	86.2(7)	O(38)-Ni(23)-O(56)	168.7(6)
O(99)-Ni(2)-O(116)	89.6(6)	O(56)-Ni(23)-Ni(24)	46.8(4)

O(99)-Ni(2)-O(118)	102.5(6)	O(56)-Ni(23)-Ni(22)	48.3(4)
O(116)-Ni(2)-O(111)	88.5(6)	O(47)-Ni(23)-Ni(22)	77.9(6)
O(116)-Ni(2)-O(118)	84.2(5)	O(47)-Ni(23)-O(54)	92.1(7)
O(109)-Ni(2)-O(111)	88.5(6)	O(47)-Ni(23)-O(38)	87.4(7)
O(109)-Ni(2)-O(99)	174.7(7)	O(47)-Ni(23)-O(56)	102.1(7)
O(109)-Ni(2)-O(116)	90.7(6)	Ni(23)-O(54)-Ni(22)	92.8(6)
O(109)-Ni(2)-O(118)	82.9(5)	Ni(21)-O(51)-Ni(24)	93.4(6)
O(106)-Ni(2)-O(111)	84.6(6)	C(541)-O(57)-Ni(21)	130.6(17)
O(117)-Ni(3)-O(118)	82.8(6)	O(49)-Ni(24)-O(50)	92.8(7)
O(117)-Ni(3)-O(113)	89.0(6)	O(49)-Ni(24)-O(58)	84.2(7)
O(103)-Ni(3)-O(117)	176.7(7)	O(40)-Ni(24)-Ni(23)	132.8(5)
O(103)-Ni(3)-O(118)	100.1(6)	O(40)-Ni(24)-O(56)	169.7(6)
O(103)-Ni(3)-O(113)	88.4(7)	O(56)-Ni(24)-Ni(21)	47.8(4)
O(102)-Ni(3)-O(117)	91.9(6)	O(51)-Ni(21)-Ni(22)	107.6(5)
O(102)-Ni(3)-O(103)	86.1(7)	O(51)-Ni(21)-O(42)	87.8(6)
O(102)-Ni(3)-O(118)	101.5(6)	O(51)-Ni(21)-O(57)	92.2(6)
O(102)-Ni(3)-O(113)	86.5(6)	O(51)-Ni(21)-O(43)	90.4(6)
O(113)-Ni(3)-O(118)	168.6(6)	O(44)-Ni(21)-O(42)	87.5(7)
O(117)-Ni(1)-O(118)	84.1(6)	O(42)-Ni(21)-Ni(24)	127.9(5)
O(116)-Ni(1)-O(117)	91.2(6)	O(42)-Ni(21)-O(43)	89.4(6)
O(116)-Ni(1)-O(118)	85.9(5)	O(42)-Ni(21)-O(56)	167.1(6)
O(101)-Ni(1)-O(117)	93.1(6)	O(57)-Ni(21)-O(43)	177.3(7)
O(101)-Ni(1)-O(116)	173.3(6)	O(57)-Ni(21)-O(56)	98.3(6)
O(101)-Ni(1)-O(114)	89.6(6)	O(43)-Ni(21)-Ni(24)	105.8(4)
O(101)-Ni(1)-O(118)	99.6(6)	O(43)-Ni(21)-Ni(22)	43.2(4)
O(101)-Ni(1)-O(100)	82.7(7)	O(43)-Ni(21)-O(56)	82.4(6)
O(114)-Ni(1)-O(117)	87.8(6)	Ni(21)-Ni(22)-Ni(23)	89.43(18)
O(114)-Ni(1)-O(116)	85.4(6)	O(54)-Ni(22)-Ni(21)	105.2(5)
O(114)-Ni(1)-O(118)	168.1(6)	O(54)-Ni(22)-Ni(23)	43.0(4)
O(100)-Ni(1)-O(117)	170.0(7)	O(55)-Ni(22)-O(56)	166.5(5)
O(100)-Ni(1)-O(116)	92.2(6)	O(43)-Ni(22)-Ni(21)	45.5(5)
O(100)-Ni(1)-O(114)	83.0(7)	O(43)-Ni(22)-Ni(23)	105.1(5)
O(100)-Ni(1)-O(118)	105.5(6)	O(43)-Ni(22)-O(54)	88.1(6)
O(108)-Ni(4)-Ni(3)	126.6(4)	O(56)-Ni(22)-Ni(21)	48.2(4)
O(108)-Ni(4)-Ni(2)	127.6(4)	O(56)-Ni(22)-Ni(23)	45.4(4)
O(108)-Ni(4)-O(109)	89.6(6)	O(46)-Ni(22)-Ni(21)	137.7(6)
O(108)-Ni(4)-O(118)	167.6(6)	O(46)-Ni(22)-Ni(23)	77.9(6)
O(105)-Ni(4)-Ni(3)	139.5(4)	O(46)-Ni(22)-O(54)	92.5(7)
O(105)-Ni(4)-Ni(2)	79.9(5)	O(46)-Ni(22)-O(55)	86.8(7)
O(105)-Ni(4)-O(108)	87.7(6)	O(46)-Ni(22)-O(43)	176.1(8)
O(105)-Ni(4)-O(109)	93.0(6)	O(46)-Ni(22)-O(45)	83.0(7)
O(105)-Ni(4)-O(118)	101.6(6)	O(46)-Ni(22)-O(56)	99.9(7)
O(105)-Ni(4)-O(104)	82.4(6)	Ni(24)-Ni(23)-Ni(22)	90.65(18)
O(109)-Ni(4)-O(118)	81.8(5)	O(54)-Ni(23)-O(50)	89.2(6)

O(104)-Ni(4)-O(108)	88.8(6)	O(48)-Ni(23)-Ni(24)	76.0(5)
O(104)-Ni(4)-O(109)	175.2(7)	O(48)-Ni(23)-Ni(22)	136.8(5)
O(104)-Ni(4)-O(118)	100.5(6)	O(48)-Ni(23)-O(54)	177.2(8)
O(111)-Ni(2)-O(118)	168.7(6)	O(38)-Ni(23)-Ni(24)	127.5(5)
O(99)-Ni(2)-O(111)	86.2(7)	O(38)-Ni(23)-O(56)	168.7(6)
O(99)-Ni(2)-O(116)	89.6(6)	O(56)-Ni(23)-Ni(24)	46.8(4)
O(99)-Ni(2)-O(118)	102.5(6)	O(56)-Ni(23)-Ni(22)	48.3(4)
O(116)-Ni(2)-O(111)	88.5(6)	O(47)-Ni(23)-Ni(22)	77.9(6)
O(116)-Ni(2)-O(118)	84.2(5)	O(47)-Ni(23)-O(54)	92.1(7)
O(109)-Ni(2)-O(111)	88.5(6)	O(47)-Ni(23)-O(38)	87.4(7)
O(109)-Ni(2)-O(99)	174.7(7)	O(47)-Ni(23)-O(56)	102.1(7)
O(109)-Ni(2)-O(116)	90.7(6)	Ni(23)-O(54)-Ni(22)	92.8(6)
O(109)-Ni(2)-O(118)	82.9(5)	Ni(21)-O(51)-Ni(24)	93.4(6)
O(106)-Ni(2)-O(111)	84.6(6)	C(541)-O(57)-Ni(21)	130.6(17)
O(117)-Ni(3)-O(118)	82.8(6)	O(49)-Ni(24)-O(50)	92.8(7)
O(117)-Ni(3)-O(113)	89.0(6)	O(49)-Ni(24)-O(58)	84.2(7)
O(103)-Ni(3)-O(117)	176.7(7)	O(40)-Ni(24)-Ni(23)	132.8(5)
O(103)-Ni(3)-O(118)	100.1(6)	O(40)-Ni(24)-O(56)	169.7(6)
O(103)-Ni(3)-O(113)	88.4(7)	O(56)-Ni(24)-Ni(21)	47.8(4)
O(102)-Ni(3)-O(117)	91.9(6)	O(51)-Ni(21)-Ni(22)	107.6(5)
O(102)-Ni(3)-O(103)	86.1(7)	O(51)-Ni(21)-O(42)	87.8(6)
O(102)-Ni(3)-O(118)	101.5(6)	O(51)-Ni(21)-O(57)	92.2(6)
O(102)-Ni(3)-O(113)	86.5(6)	O(51)-Ni(21)-O(43)	90.4(6)
O(113)-Ni(3)-O(118)	168.6(6)	O(44)-Ni(21)-O(42)	87.5(7)
O(116)-Ni(1)-O(117)	91.2(6)	O(42)-Ni(21)-O(43)	89.4(6)
O(116)-Ni(1)-O(118)	85.9(5)	O(42)-Ni(21)-O(56)	167.1(6)
O(101)-Ni(1)-O(117)	93.1(6)	O(57)-Ni(21)-O(43)	177.3(7)
O(101)-Ni(1)-O(116)	173.3(6)	O(57)-Ni(21)-O(56)	98.3(6)
O(101)-Ni(1)-O(114)	89.6(6)	O(43)-Ni(21)-Ni(24)	105.8(4)
O(101)-Ni(1)-O(118)	99.6(6)	O(43)-Ni(21)-Ni(22)	43.2(4)
O(101)-Ni(1)-O(100)	82.7(7)	O(43)-Ni(21)-O(56)	82.4(6)
O(114)-Ni(1)-O(117)	87.8(6)	Ni(21)-Ni(22)-Ni(23)	89.43(18)
O(114)-Ni(1)-O(116)	85.4(6)	O(54)-Ni(22)-Ni(21)	105.2(5)
O(114)-Ni(1)-O(118)	168.1(6)	O(54)-Ni(22)-Ni(23)	43.0(4)
O(100)-Ni(1)-O(117)	170.0(7)	O(55)-Ni(22)-O(56)	166.5(5)
O(100)-Ni(1)-O(116)	92.2(6)	O(43)-Ni(22)-Ni(21)	45.5(5)
O(100)-Ni(1)-O(114)	83.0(7)	O(43)-Ni(22)-Ni(23)	105.1(5)
O(100)-Ni(1)-O(118)	105.5(6)	O(43)-Ni(22)-O(54)	88.1(6)
O(8AA)-Ni(4)-O(118)	84.4(6)	O(45)-Ni(22)-O(43)	95.9(7)
O(8AA)-Ni(4)-O(104)	92.9(6)	O(45)-Ni(22)-O(56)	107.6(6)
O(108)-Ni(4)-Ni(3)	126.6(4)	O(56)-Ni(22)-Ni(21)	48.2(4)
O(108)-Ni(4)-Ni(2)	127.6(4)	O(56)-Ni(22)-Ni(23)	45.4(4)
O(108)-Ni(4)-O(109)	89.6(6)	O(46)-Ni(22)-Ni(21)	137.7(6)
O(108)-Ni(4)-O(118)	167.6(6)	O(46)-Ni(22)-Ni(23)	77.9(6)

O(105)-Ni(4)-O(108)	87.7(6)	O(46)-Ni(22)-O(43)	176.1(8)
O(105)-Ni(4)-O(109)	93.0(6)	O(46)-Ni(22)-O(45)	83.0(7)
O(105)-Ni(4)-O(118)	101.6(6)	O(46)-Ni(22)-O(56)	99.9(7)
O(105)-Ni(4)-O(104)	82.4(6)	Ni(24)-Ni(23)-Ni(22)	90.65(18)
O(109)-Ni(4)-O(118)	81.8(5)	O(54)-Ni(23)-O(50)	89.2(6)
O(104)-Ni(4)-O(108)	88.8(6)	O(48)-Ni(23)-Ni(24)	76.0(5)
O(104)-Ni(4)-O(109)	175.2(7)	O(48)-Ni(23)-Ni(22)	136.8(5)
O(104)-Ni(4)-O(118)	100.5(6)	O(48)-Ni(23)-O(54)	177.2(8)
O(111)-Ni(2)-O(118)	168.7(6)	O(38)-Ni(23)-Ni(24)	127.5(5)
O(99)-Ni(2)-O(111)	86.2(7)	O(38)-Ni(23)-O(56)	168.7(6)
O(99)-Ni(2)-O(116)	89.6(6)	O(56)-Ni(23)-Ni(24)	46.8(4)
O(99)-Ni(2)-O(118)	102.5(6)	O(56)-Ni(23)-Ni(22)	48.3(4)
O(109)-Ni(2)-O(111)	88.5(6)	O(47)-Ni(23)-O(38)	87.4(7)
O(109)-Ni(2)-O(99)	174.7(7)	O(47)-Ni(23)-O(56)	102.1(7)
O(109)-Ni(2)-O(116)	90.7(6)	Ni(23)-O(54)-Ni(22)	92.8(6)
O(109)-Ni(2)-O(118)	82.9(5)	Ni(21)-O(51)-Ni(24)	93.4(6)
O(106)-Ni(2)-O(111)	84.6(6)	C(541)-O(57)-Ni(21)	130.6(17)
O(117)-Ni(3)-O(118)	82.8(6)	O(49)-Ni(24)-O(50)	92.8(7)
O(117)-Ni(3)-O(113)	89.0(6)	O(49)-Ni(24)-O(58)	84.2(7)
O(103)-Ni(3)-O(117)	176.7(7)	O(40)-Ni(24)-Ni(23)	132.8(5)
O(103)-Ni(3)-O(118)	100.1(6)	O(40)-Ni(24)-O(56)	169.7(6)
O(103)-Ni(3)-O(113)	88.4(7)	O(56)-Ni(24)-Ni(21)	47.8(4)
O(102)-Ni(3)-O(117)	91.9(6)	O(51)-Ni(21)-Ni(22)	107.6(5)
O(102)-Ni(3)-O(103)	86.1(7)	O(51)-Ni(21)-O(42)	87.8(6)
O(102)-Ni(3)-O(118)	101.5(6)	O(51)-Ni(21)-O(57)	92.2(6)
O(102)-Ni(3)-O(113)	86.5(6)	O(51)-Ni(21)-O(43)	90.4(6)
O(117)-Ni(1)-O(118)	84.1(6)	O(42)-Ni(21)-Ni(24)	127.9(5)
O(116)-Ni(1)-O(117)	91.2(6)	O(42)-Ni(21)-O(43)	89.4(6)
O(116)-Ni(1)-O(118)	85.9(5)	O(42)-Ni(21)-O(56)	167.1(6)

Supplementary Table 3. Selected bond lengths [Å] and angles [°] for 1-Co

Co(15)-O(52)	2.083(3)	Co(2)-O(12)	2.057(3)
Co(15)-O(58)	2.113(3)	Co(2)-O(17)	2.130(3)
Co(15)-O(49)	2.033(3)	Co(2)-O(11)	2.123(3)
Co(15)-O(51)	2.081(3)	Co(2)-O(18)	2.208(3)
Co(15)-O(57)	2.211(3)	Co(2)-O(167)	2.057(3)
Co(15)-O(166)	2.009(3)	Co(2)-O(15)	1.947(3)
Co(16)-O(60)	2.110(3)	Co(4)-O(16)	2.151(3)
Co(16)-O(62)	2.115(3)	Co(4)-O(20)	2.052(3)
Co(16)-O(58)	2.079(3)	Co(4)-O(169)	2.020(3)
Co(16)-O(57)	2.235(3)	Co(4)-O(18)	2.173(3)
Co(16)-O(68)	2.045(3)	Co(4)-O(14)	2.009(3)
Co(16)-O(69)	2.034(3)	Co(4)-O(21)	2.122(3)
Co(7)-O(133)	2.173(3)	Co(14)-O(48)	2.020(3)
Co(7)-O(142)	2.102(3)	Co(14)-O(51)	2.066(3)
Co(7)-O(139)	2.048(3)	Co(14)-O(50)	2.118(3)
Co(7)-O(131)	2.098(3)	Co(14)-O(57)	2.248(3)
Co(7)-O(140)	2.096(3)	Co(14)-O(59)	2.089(3)
Co(7)-O(141)	2.023(3)	Co(14)-O(55)	2.042(3)
Co(13)-O(60)	2.046(2)	Co(24)-O(101)	2.194(3)
Co(13)-O(57)	2.169(3)	Co(24)-O(107)	2.094(3)
Co(13)-O(56)	2.025(3)	Co(24)-O(100)	2.090(3)
Co(13)-O(59)	2.112(3)	Co(24)-O(105)	2.066(3)
Co(13)-O(65)	2.030(3)	Co(24)-O(98)	2.051(3)
Co(13)-O(61)	2.112(3)	Co(24)-O(94)	2.042(3)
Co(9)-O(111)	2.097(3)	Co(3)-O(22)	2.101(3)
Co(9)-O(129)	2.152(3)	Co(3)-O(17)	2.093(3)
Co(9)-O(112)	2.135(3)	Co(3)-O(20)	2.157(3)
Co(9)-O(126)	2.099(3)	Co(3)-O(18)	2.263(3)
Co(9)-O(115)	2.022(3)	Co(3)-O(19)	2.047(3)
Co(9)-O(123)	2.016(3)	Co(3)-O(170)	1.965(3)
Co(23)-O(106)	2.089(3)	Co(21)-O(106)	2.069(3)
Co(23)-O(101)	2.273(3)	Co(21)-O(101)	2.144(3)
Co(23)-O(107)	2.052(3)	Co(21)-O(102)	2.045(4)
Co(23)-O(99)	2.038(3)	Co(21)-O(97)	2.053(3)
Co(23)-O(91)	2.110(3)	Co(21)-O(104)	2.076(3)
Co(23)-O(103)	2.038(3)	Co(21)-O(93)	2.111(3)
Co(10)-O(129)	2.273(3)	Co(5)-O(133)	2.262(3)
Co(10)-O(112)	2.073(3)	Co(5)-O(132)	2.091(3)
Co(10)-O(127)	2.090(3)	Co(5)-O(142)	2.112(3)
Co(10)-O(124)	2.004(3)	Co(5)-O(143)	2.084(3)
Co(10)-O(125)	1.985(3)	Co(5)-O(171)	1.981(3)
Co(10)-O(114)	2.115(3)	Co(5)-O(135)	2.049(3)
Co(22)-O(101)	2.269(3)	Co(11)-O(129)	2.272(3)

Co(22)-O(38)	2.105(3)	Co(11)-O(128)	2.136(3)
Co(22)-O(105)	2.037(3)	Co(11)-O(126)	2.062(3)
Co(22)-O(95)	2.027(3)	Co(11)-O(117)	1.995(3)
Co(22)-O(96)	2.005(3)	Co(11)-O(116)	1.995(3)
Co(22)-O(104)	2.084(3)	Co(11)-O(122)	2.120(3)
Co(8)-O(133)	2.261(3)	Co(20)-O(154)	2.055(3)
Co(8)-O(82)	2.049(3)	Co(20)-O(164)	2.011(3)
Co(8)-O(138)	2.002(3)	Co(20)-O(165)	2.102(3)
Co(8)-O(134)	2.078(3)	Co(20)-O(159)	2.312(3)
Co(8)-O(140)	2.051(3)	Co(20)-O(158)	2.003(3)
Co(8)-O(81)	2.116(3)	Co(20)-O(39)	2.091(4)
Co(17)-O(163)	2.066(3)	Co(1)-O(12)	2.115(3)
Co(17)-O(155)	2.001(3)	Co(1)-O(16)	2.074(3)
Co(17)-O(152)	2.092(3)	Co(1)-O(13)	2.048(3)
Co(17)-O(151)	2.127(3)	Co(1)-O(10)	2.109(3)
Co(17)-O(160)	2.044(3)	Co(1)-O(18)	2.220(3)
Co(17)-O(159)	2.259(3)	Co(1)-O(168)	1.981(3)
Co(12)-O(129)	2.131(3)	Co(6)-O(133)	2.136(3)
Co(12)-O(128)	2.135(3)	Co(6)-O(134)	2.110(3)
Co(12)-O(127)	2.071(3)	Co(6)-O(137)	2.113(4)
Co(12)-O(119)	2.131(3)	Co(6)-O(143)	2.044(3)
Co(12)-O(172)	2.030(3)	Co(6)-O(83)	2.021(3)
Co(12)-O(118)	2.009(3)	Co(6)-O(136)	2.036(4)
Co(19)-O(154)	2.109(3)	Co(18)-O(163)	2.104(3)
Co(19)-O(156)	2.024(3)	Co(18)-O(165)	2.059(3)
Co(19)-O(152)	2.099(3)	Co(18)-O(148)	2.104(3)
Co(19)-O(159)	2.166(3)	Co(18)-O(159)	2.133(3)
Co(19)-O(157)	2.001(3)	Co(18)-O(162)	1.993(3)
Co(19)-O(153)	2.108(4)	Co(18)-O(161)	2.031(4)
O(52)-Co(15)-O(58)	88.70(10)	O(12)-Co(2)-O(17)	90.44(11)
O(52)-Co(15)-O(57)	168.00(9)	O(12)-Co(2)-O(11)	86.25(11)
O(58)-Co(15)-O(57)	82.94(10)	O(12)-Co(2)-O(18)	84.81(11)
O(49)-Co(15)-O(52)	90.55(11)	O(17)-Co(2)-O(18)	83.81(11)
O(49)-Co(15)-O(58)	175.98(10)	O(11)-Co(2)-O(17)	85.57(12)
O(49)-Co(15)-O(51)	94.16(11)	O(11)-Co(2)-O(18)	166.06(11)
O(49)-Co(15)-O(57)	98.37(11)	O(167)-Co(2)-O(12)	92.57(12)
O(51)-Co(15)-O(52)	87.19(10)	O(167)-Co(2)-O(17)	175.94(11)
O(51)-Co(15)-O(58)	89.75(11)	O(167)-Co(2)-O(11)	91.90(13)
O(51)-Co(15)-O(57)	84.19(10)	O(167)-Co(2)-O(18)	99.17(12)
O(166)-Co(15)-O(52)	86.26(12)	O(15)-Co(2)-O(12)	172.95(14)
O(166)-Co(15)-O(58)	92.01(12)	O(15)-Co(2)-O(17)	93.02(13)
O(166)-Co(15)-O(49)	84.00(12)	O(15)-Co(2)-O(11)	87.89(14)
O(166)-Co(15)-O(51)	173.17(12)	O(15)-Co(2)-O(18)	101.66(13)

O(166)-Co(15)-O(57)	102.57(11)	O(15)-Co(2)-O(167)	83.69(14)
O(60)-Co(16)-O(62)	86.09(10)	O(16)-Co(4)-O(18)	82.78(11)
O(60)-Co(16)-O(57)	81.63(10)	O(20)-Co(4)-O(16)	87.73(12)
O(62)-Co(16)-O(57)	165.87(9)	O(20)-Co(4)-O(18)	85.00(11)
O(58)-Co(16)-O(60)	87.84(11)	O(20)-Co(4)-O(21)	88.39(12)
O(58)-Co(16)-O(62)	89.47(10)	O(169)-Co(4)-O(16)	175.61(12)
O(58)-Co(16)-O(57)	83.13(10)	O(169)-Co(4)-O(20)	93.54(13)
O(68)-Co(16)-Co(15)	138.72(8)	O(169)-Co(4)-O(18)	101.51(12)
O(68)-Co(16)-O(60)	92.16(11)	O(169)-Co(4)-O(21)	89.89(13)
O(68)-Co(16)-O(62)	86.50(11)	O(14)-Co(4)-O(16)	93.41(13)
O(68)-Co(16)-O(58)	175.96(10)	O(14)-Co(4)-O(20)	174.59(13)
O(68)-Co(16)-O(57)	100.87(10)	O(14)-Co(4)-O(169)	84.95(14)
O(69)-Co(16)-O(60)	173.86(11)	O(14)-Co(4)-O(18)	100.39(13)
O(69)-Co(16)-O(62)	88.90(11)	O(14)-Co(4)-O(21)	86.41(13)
O(69)-Co(16)-O(58)	95.67(11)	O(21)-Co(4)-O(16)	85.95(12)
O(69)-Co(16)-O(57)	103.76(11)	O(21)-Co(4)-O(18)	167.13(12)
O(69)-Co(16)-O(68)	83.99(12)	O(48)-Co(14)-O(51)	94.34(12)
O(142)-Co(7)-O(133)	82.69(11)	O(48)-Co(14)-O(50)	88.25(12)
O(139)-Co(7)-O(133)	100.27(11)	O(48)-Co(14)-O(57)	100.22(11)
O(139)-Co(7)-O(142)	176.95(11)	O(48)-Co(14)-O(59)	175.15(10)
O(139)-Co(7)-O(131)	86.88(12)	O(48)-Co(14)-O(55)	83.76(12)
O(139)-Co(7)-O(140)	93.81(12)	O(51)-Co(14)-O(50)	86.48(10)
O(131)-Co(7)-O(133)	167.76(12)	O(51)-Co(14)-O(57)	83.62(10)
O(131)-Co(7)-O(142)	90.33(11)	O(51)-Co(14)-O(59)	89.36(10)
O(140)-Co(7)-O(133)	83.70(11)	O(50)-Co(14)-O(57)	167.41(9)
O(140)-Co(7)-O(142)	87.23(11)	O(57)-Co(14)-Co(15)	47.21(7)
O(140)-Co(7)-O(131)	85.94(12)	O(59)-Co(14)-O(50)	88.85(11)
O(141)-Co(7)-O(133)	101.39(12)	O(59)-Co(14)-O(57)	83.29(10)
O(141)-Co(7)-O(142)	94.55(12)	O(55)-Co(14)-O(51)	174.81(11)
O(141)-Co(7)-O(139)	84.17(12)	O(55)-Co(14)-O(50)	88.63(11)
O(141)-Co(7)-O(131)	89.14(13)	O(55)-Co(14)-O(57)	101.45(11)
O(141)-Co(7)-O(140)	174.78(11)	O(55)-Co(14)-O(59)	92.29(11)
O(60)-Co(13)-O(57)	84.72(10)	O(107)-Co(24)-O(101)	83.99(11)
O(60)-Co(13)-O(59)	90.56(10)	O(100)-Co(24)-O(101)	168.41(12)
O(60)-Co(13)-O(61)	87.96(11)	O(100)-Co(24)-O(107)	89.38(12)
O(57)-Co(13)-Co(14)	48.56(7)	O(105)-Co(24)-O(101)	84.21(12)
O(56)-Co(13)-O(60)	171.26(12)	O(105)-Co(24)-O(107)	90.57(12)
O(56)-Co(13)-O(57)	102.73(11)	O(105)-Co(24)-O(100)	86.37(13)
O(56)-Co(13)-O(59)	94.63(11)	O(98)-Co(24)-O(101)	101.52(13)
O(56)-Co(13)-O(65)	82.04(12)	O(98)-Co(24)-O(107)	94.30(12)
O(56)-Co(13)-O(61)	85.27(12)	O(98)-Co(24)-O(100)	88.42(14)
O(59)-Co(13)-O(57)	84.70(10)	O(98)-Co(24)-O(105)	172.83(12)
O(59)-Co(13)-O(61)	87.30(11)	O(94)-Co(24)-O(101)	97.00(12)
O(65)-Co(13)-O(60)	92.13(12)	O(94)-Co(24)-O(107)	176.52(12)

O(65)-Co(13)-O(57)	101.05(12)	O(94)-Co(24)-O(100)	90.18(13)
O(65)-Co(13)-O(59)	173.84(11)	O(94)-Co(24)-O(105)	92.85(12)
O(65)-Co(13)-O(61)	87.27(12)	O(94)-Co(24)-O(98)	82.24(13)
O(61)-Co(13)-O(57)	169.10(9)	O(22)-Co(3)-O(20)	85.85(11)
O(111)-Co(9)-O(129)	166.58(10)	O(22)-Co(3)-O(18)	164.75(10)
O(111)-Co(9)-O(112)	88.17(11)	O(17)-Co(3)-O(22)	89.14(12)
O(112)-Co(9)-O(129)	82.86(11)	O(17)-Co(3)-O(20)	86.13(11)
O(126)-Co(9)-O(129)	83.80(10)	O(17)-Co(3)-O(18)	83.33(11)
O(126)-Co(9)-O(112)	87.23(11)	O(20)-Co(3)-O(18)	80.44(11)
O(115)-Co(9)-O(111)	88.39(12)	O(19)-Co(3)-O(22)	88.76(13)
O(115)-Co(9)-O(129)	100.74(12)	O(19)-Co(3)-O(17)	94.48(12)
O(115)-Co(9)-O(112)	176.36(12)	O(19)-Co(3)-O(20)	174.56(12)
O(115)-Co(9)-O(126)	93.71(12)	O(19)-Co(3)-O(18)	104.99(12)
O(123)-Co(9)-O(111)	89.33(11)	O(170)-Co(3)-O(22)	88.23(13)
O(123)-Co(9)-O(129)	101.41(11)	O(170)-Co(3)-O(17)	177.35(13)
O(123)-Co(9)-O(112)	95.46(12)	O(170)-Co(3)-O(20)	93.29(12)
O(123)-Co(9)-O(126)	174.38(11)	O(170)-Co(3)-O(18)	99.13(13)
O(123)-Co(9)-O(115)	83.30(13)	O(170)-Co(3)-O(19)	85.85(13)
O(106)-Co(23)-O(101)	80.93(11)	O(106)-Co(21)-O(101)	84.56(11)
O(106)-Co(23)-O(91)	88.12(12)	O(106)-Co(21)-O(104)	88.87(12)
O(107)-Co(23)-O(106)	87.82(12)	O(106)-Co(21)-O(93)	87.59(11)
O(107)-Co(23)-O(101)	82.98(11)	O(102)-Co(21)-O(106)	93.12(13)
O(107)-Co(23)-O(91)	90.54(12)	O(102)-Co(21)-O(101)	99.54(13)
O(99)-Co(23)-O(106)	174.73(12)	O(102)-Co(21)-O(97)	82.82(13)
O(99)-Co(23)-O(101)	104.27(11)	O(102)-Co(21)-O(104)	175.52(11)
O(99)-Co(23)-O(107)	93.62(13)	O(102)-Co(21)-O(93)	87.66(13)
O(99)-Co(23)-O(91)	86.80(12)	O(97)-Co(21)-O(106)	172.73(12)
O(91)-Co(23)-O(101)	167.45(12)	O(97)-Co(21)-O(101)	102.03(11)
O(103)-Co(23)-O(106)	92.61(13)	O(97)-Co(21)-O(104)	94.76(12)
O(103)-Co(23)-O(101)	100.91(12)	O(97)-Co(21)-O(93)	86.22(11)
O(103)-Co(23)-O(107)	176.11(12)	O(104)-Co(21)-O(101)	84.64(11)
O(103)-Co(23)-O(99)	85.61(13)	O(104)-Co(21)-O(93)	88.41(11)
O(103)-Co(23)-O(91)	85.62(13)	O(93)-Co(21)-O(101)	169.61(11)
O(112)-Co(10)-O(129)	81.36(11)	O(132)-Co(5)-O(133)	165.80(12)
O(112)-Co(10)-O(127)	87.05(11)	O(132)-Co(5)-O(142)	89.31(12)
O(112)-Co(10)-O(114)	89.53(12)	O(142)-Co(5)-O(133)	80.38(11)
O(127)-Co(10)-O(129)	80.99(11)	O(143)-Co(5)-O(133)	81.50(11)
O(127)-Co(10)-O(114)	86.21(11)	O(143)-Co(5)-O(132)	88.34(12)
O(124)-Co(10)-O(129)	106.41(11)	O(143)-Co(5)-O(142)	87.31(12)
O(124)-Co(10)-O(112)	94.46(12)	O(171)-Co(5)-O(133)	103.39(12)
O(124)-Co(10)-O(127)	172.58(12)	O(171)-Co(5)-O(132)	87.07(13)
O(124)-Co(10)-O(114)	86.54(12)	O(171)-Co(5)-O(142)	94.82(12)
O(125)-Co(10)-O(129)	100.82(12)	O(171)-Co(5)-O(143)	174.92(12)
O(125)-Co(10)-O(112)	177.10(13)	O(171)-Co(5)-O(135)	87.13(14)

O(125)-Co(10)-O(127)	91.39(12)	O(135)-Co(5)-O(133)	103.67(12)
O(125)-Co(10)-O(124)	86.78(12)	O(135)-Co(5)-O(132)	86.20(13)
O(125)-Co(10)-O(114)	87.92(13)	O(135)-Co(5)-O(142)	175.01(11)
O(114)-Co(10)-O(129)	164.61(10)	O(135)-Co(5)-O(143)	90.39(14)
O(38)-Co(22)-O(101)	168.31(10)	O(128)-Co(11)-O(129)	80.78(10)
O(105)-Co(22)-O(101)	82.98(11)	O(126)-Co(11)-O(129)	81.71(11)
O(105)-Co(22)-O(38)	88.55(12)	O(126)-Co(11)-O(128)	86.75(12)
O(105)-Co(22)-O(104)	89.34(11)	O(126)-Co(11)-O(122)	87.24(11)
O(95)-Co(22)-O(101)	101.62(12)	O(117)-Co(11)-O(129)	104.42(12)
O(95)-Co(22)-O(38)	86.83(12)	O(117)-Co(11)-O(128)	93.13(13)
O(95)-Co(22)-O(105)	93.19(12)	O(117)-Co(11)-O(126)	173.77(11)
O(95)-Co(22)-O(104)	176.29(12)	O(117)-Co(11)-O(122)	86.53(12)
O(96)-Co(22)-O(101)	102.75(12)	O(116)-Co(11)-O(129)	103.45(12)
O(96)-Co(22)-O(38)	85.71(12)	O(116)-Co(11)-O(128)	175.34(11)
O(96)-Co(22)-O(105)	174.25(13)	O(116)-Co(11)-O(126)	91.90(13)
O(96)-Co(22)-O(95)	86.16(12)	O(116)-Co(11)-O(117)	87.72(14)
O(96)-Co(22)-O(104)	91.05(12)	O(116)-Co(11)-O(122)	87.10(13)
O(104)-Co(22)-O(101)	81.37(11)	O(122)-Co(11)-O(129)	164.90(12)
O(104)-Co(22)-O(38)	90.53(12)	O(122)-Co(11)-O(128)	88.38(11)
O(82)-Co(8)-O(133)	103.26(12)	O(154)-Co(20)-O(165)	87.42(12)
O(82)-Co(8)-O(134)	92.84(12)	O(154)-Co(20)-O(159)	81.45(12)
O(82)-Co(8)-O(140)	174.08(13)	O(154)-Co(20)-O(39)	91.15(14)
O(82)-Co(8)-O(81)	85.88(12)	O(164)-Co(20)-O(154)	178.52(14)
O(138)-Co(8)-O(133)	102.63(13)	O(164)-Co(20)-O(165)	93.14(13)
O(138)-Co(8)-O(82)	85.46(12)	O(164)-Co(20)-O(159)	99.98(13)
O(138)-Co(8)-O(134)	175.11(13)	O(164)-Co(20)-O(39)	87.52(15)
O(138)-Co(8)-O(140)	92.12(12)	O(165)-Co(20)-O(159)	81.10(11)
O(138)-Co(8)-O(81)	86.36(13)	O(158)-Co(20)-O(154)	92.66(13)
O(134)-Co(8)-O(133)	82.21(11)	O(158)-Co(20)-O(164)	86.64(13)
O(134)-Co(8)-O(81)	88.95(12)	O(158)-Co(20)-O(165)	173.58(13)
O(140)-Co(8)-O(133)	82.54(11)	O(158)-Co(20)-O(159)	105.27(12)
O(140)-Co(8)-O(134)	89.13(11)	O(158)-Co(20)-O(39)	87.44(14)
O(140)-Co(8)-O(81)	88.58(12)	O(39)-Co(20)-O(165)	86.14(13)
O(81)-Co(8)-O(133)	167.53(10)	O(39)-Co(20)-O(159)	165.49(12)
O(163)-Co(17)-O(152)	88.07(12)	O(12)-Co(1)-O(18)	83.19(11)
O(163)-Co(17)-O(151)	90.13(11)	O(16)-Co(1)-O(12)	88.06(12)
O(163)-Co(17)-O(159)	82.36(11)	O(16)-Co(1)-O(10)	89.13(11)
O(155)-Co(17)-O(163)	175.90(12)	O(16)-Co(1)-O(18)	83.44(11)
O(155)-Co(17)-O(152)	93.39(13)	O(13)-Co(1)-O(12)	172.51(13)
O(155)-Co(17)-O(151)	86.13(13)	O(13)-Co(1)-O(16)	93.31(13)
O(155)-Co(17)-O(160)	87.09(14)	O(13)-Co(1)-O(10)	87.80(12)
O(155)-Co(17)-O(159)	101.63(12)	O(13)-Co(1)-O(18)	104.28(13)
O(152)-Co(17)-O(151)	86.58(12)	O(10)-Co(1)-O(12)	84.85(11)
O(152)-Co(17)-O(159)	81.96(11)	O(10)-Co(1)-O(18)	166.11(10)

O(151)-Co(17)-O(159)	166.48(12)	O(168)-Co(1)-O(12)	94.17(13)
O(160)-Co(17)-O(163)	90.83(13)	O(168)-Co(1)-O(16)	176.15(12)
O(160)-Co(17)-O(152)	170.42(12)	O(168)-Co(1)-O(13)	84.08(14)
O(160)-Co(17)-O(151)	83.91(13)	O(168)-Co(1)-O(10)	87.95(12)
O(160)-Co(17)-O(159)	107.32(12)	O(168)-Co(1)-O(18)	99.93(12)
O(129)-Co(12)-O(128)	84.13(11)	O(134)-Co(6)-O(133)	84.57(12)
O(129)-Co(12)-O(119)	167.89(11)	O(134)-Co(6)-O(137)	87.13(14)
O(127)-Co(12)-O(129)	84.88(12)	O(137)-Co(6)-O(133)	168.78(12)
O(127)-Co(12)-O(128)	88.45(12)	O(143)-Co(6)-O(133)	85.57(11)
O(127)-Co(12)-O(119)	86.79(12)	O(143)-Co(6)-O(134)	90.24(12)
O(119)-Co(12)-O(128)	86.87(11)	O(143)-Co(6)-O(137)	86.93(12)
O(172)-Co(12)-O(129)	101.42(11)	O(83)-Co(6)-O(133)	99.13(12)
O(172)-Co(12)-O(128)	174.30(11)	O(83)-Co(6)-O(134)	93.74(13)
O(172)-Co(12)-O(127)	93.30(12)	O(83)-Co(6)-O(137)	88.91(13)
O(172)-Co(12)-O(119)	87.81(12)	O(83)-Co(6)-O(143)	174.10(13)
O(118)-Co(12)-O(129)	101.51(13)	O(83)-Co(6)-O(136)	82.01(14)
O(118)-Co(12)-O(128)	94.08(12)	O(136)-Co(6)-O(133)	102.55(14)
O(118)-Co(12)-O(127)	173.33(12)	O(136)-Co(6)-O(134)	172.17(13)
O(118)-Co(12)-O(119)	87.18(13)	O(136)-Co(6)-O(137)	86.22(15)
O(118)-Co(12)-O(172)	83.61(13)	O(136)-Co(6)-O(143)	93.53(13)
O(154)-Co(19)-O(159)	83.83(12)	O(163)-Co(18)-O(148)	86.84(12)
O(156)-Co(19)-O(154)	178.49(13)	O(163)-Co(18)-O(159)	84.58(11)
O(156)-Co(19)-O(152)	91.63(13)	O(165)-Co(18)-O(163)	89.34(12)
O(156)-Co(19)-O(159)	97.65(13)	O(165)-Co(18)-O(148)	86.81(13)
O(156)-Co(19)-O(153)	89.41(15)	O(165)-Co(18)-O(159)	86.55(12)
O(152)-Co(19)-O(154)	88.22(12)	O(148)-Co(18)-O(159)	169.20(12)
O(152)-Co(19)-O(159)	84.08(11)	O(162)-Co(18)-O(163)	175.35(13)
O(152)-Co(19)-O(153)	86.68(12)	O(162)-Co(18)-O(165)	94.34(14)
O(157)-Co(19)-O(154)	96.19(13)	O(162)-Co(18)-O(148)	90.52(13)
O(157)-Co(19)-O(156)	83.80(14)	O(162)-Co(18)-O(159)	98.46(12)
O(157)-Co(19)-O(152)	172.35(13)	O(162)-Co(18)-O(161)	81.34(14)
O(157)-Co(19)-O(159)	102.56(12)	O(161)-Co(18)-O(163)	94.64(13)
O(157)-Co(19)-O(153)	87.15(13)	O(161)-Co(18)-O(165)	171.49(13)
O(153)-Co(19)-O(154)	89.07(14)	O(161)-Co(18)-O(148)	85.91(14)
O(153)-Co(19)-O(159)	168.51(12)	O(161)-Co(18)-O(159)	101.29(13)
O(157)-Co(19)-O(152)	172.35(13)	O(162)-Co(18)-O(161)	81.34(14)
O(157)-Co(19)-O(159)	102.56(12)	O(161)-Co(18)-O(163)	94.64(13)
O(157)-Co(19)-O(153)	87.15(13)	O(161)-Co(18)-O(165)	171.49(13)
O(153)-Co(19)-O(154)	89.07(14)	O(161)-Co(18)-O(148)	85.91(14)
O(153)-Co(19)-O(159)	168.51(12)	O(161)-Co(18)-O(159)	101.29(13)

Supplementary Table 4. Selected bond lengths [Å] and angles [°] for 1-Co'

Co(3)-O(36)	1.989(3)
Co(3)-O(43)	2.286(3)
Co(3)-O(40)	2.080(2)
Co(3)-O(38)	2.042(3)
Co(3)-O(35)	1.936(3)
Co(3)-O(16)#1	1.984(5)
Co(4)-O(39)	2.034(2)
Co(4)-O(33)	2.112(3)
Co(4)-O(43)	2.196(3)
Co(4)-O(38)	2.123(3)
Co(4)-O(31)	1.966(3)
Co(4)-O(34)	1.929(3)
Co(5)-Co(2)	2.9183(9)
Co(5)-O(32)	2.068(3)
Co(5)-O(29)	2.088(3)
Co(5)-O(43)	2.107(3)
Co(5)-O(31)	2.059(3)
Co(5)-O(13)#1	1.968(2)
Co(2)-O(41)	2.055(3)
Co(2)-O(43)	2.109(3)
Co(2)-O(40)	2.064(2)
Co(2)-O(14)#1	1.997(3)
Co(2)-O(15)#1	1.971(5)

O(36)-Co(3)-Co(4)	129.04(8)
O(36)-Co(3)-O(43)	166.69(11)
O(36)-Co(3)-O(40)	89.24(10)
O(36)-Co(3)-O(38)	86.22(11)
O(43)-Co(3)-Co(4)	47.81(6)
O(40)-Co(3)-Co(4)	106.62(6)
O(40)-Co(3)-O(43)	80.62(9)
O(38)-Co(3)-Co(4)	46.36(8)
O(38)-Co(3)-O(43)	85.49(11)
O(38)-Co(3)-O(40)	91.62(10)
O(35)-Co(3)-Co(4)	78.42(10)
O(35)-Co(3)-O(36)	87.30(13)
O(35)-Co(3)-O(43)	103.34(12)
O(35)-Co(3)-O(40)	174.96(12)
O(35)-Co(3)-O(38)	91.81(13)
O(35)-Co(3)-O(16)#1	84.02(19)
O(16)#1-Co(3)-Co(4)	137.45(16)
O(16)#1-Co(3)-O(36)	87.90(18)
O(16)#1-Co(3)-O(43)	100.99(18)

O(16)#1-Co(3)-O(40)	92.20(17)
O(16)#1-Co(3)-O(38)	172.94(18)
Co(3)-Co(4)-Co(5)	91.22(2)
O(39)-Co(4)-Co(3)	134.84(8)
O(39)-Co(4)-Co(5)	120.65(8)
O(39)-Co(4)-O(33)	84.62(11)
O(39)-Co(4)-O(43)	163.47(11)
O(39)-Co(4)-O(38)	92.95(11)
O(33)-Co(4)-Co(3)	137.87(8)
O(33)-Co(4)-Co(5)	74.40(9)
O(33)-Co(4)-O(43)	96.76(11)
O(33)-Co(4)-O(38)	177.31(12)
O(43)-Co(4)-Co(3)	50.47(8)
O(43)-Co(4)-Co(5)	45.08(8)
O(38)-Co(4)-Co(3)	44.12(8)
O(38)-Co(4)-Co(5)	107.91(8)
O(38)-Co(4)-O(43)	85.90(11)
O(31)-Co(4)-Co(3)	104.58(8)
O(31)-Co(4)-Co(5)	43.61(8)
O(31)-Co(4)-O(39)	83.73(11)
O(31)-Co(4)-O(33)	91.94(12)
O(31)-Co(4)-O(43)	79.76(11)
O(31)-Co(4)-O(38)	88.93(11)
O(34)-Co(4)-Co(3)	83.62(9)
O(34)-Co(4)-Co(5)	137.14(9)
O(34)-Co(4)-O(39)	90.92(11)
O(34)-Co(4)-O(33)	81.32(12)
O(34)-Co(4)-O(43)	105.59(11)
O(34)-Co(4)-O(38)	97.60(12)
O(34)-Co(4)-O(31)	171.78(12)
Co(2)-Co(5)-Co(4)	89.34(3)
O(32)-Co(5)-Co(4)	83.78(9)
O(32)-Co(5)-Co(2)	141.34(9)
O(32)-Co(5)-O(29)	83.40(12)
O(32)-Co(5)-O(43)	107.02(11)
O(29)-Co(5)-Co(4)	130.94(9)
O(29)-Co(5)-Co(2)	127.33(9)
O(29)-Co(5)-O(43)	168.29(11)
O(43)-Co(5)-Co(4)	47.55(7)
O(43)-Co(5)-Co(2)	46.21(7)
O(31)-Co(5)-Co(4)	41.20(7)
O(31)-Co(5)-Co(2)	103.20(8)
O(31)-Co(5)-O(32)	96.33(11)
O(31)-Co(5)-O(29)	93.91(12)

O(31)-Co(5)-O(43)	79.87(10)
O(13)#1-Co(5)-Co(4)	134.52(8)
O(13)#1-Co(5)-Co(2)	79.64(8)
O(13)#1-Co(5)-O(32)	78.51(11)
O(13)#1-Co(5)-O(29)	88.26(12)
O(13)#1-Co(5)-O(43)	98.90(10)
O(13)#1-Co(5)-O(31)	174.15(11)
O(41)-Co(2)-Co(5)	127.64(9)
O(41)-Co(2)-O(43)	168.43(11)
O(41)-Co(2)-O(40)	88.53(10)
O(43)-Co(2)-Co(5)	46.17(9)
O(40)-Co(2)-Co(5)	107.93(6)
O(40)-Co(2)-O(43)	85.35(10)
O(14)#1-Co(2)-Co(5)	82.16(9)
O(14)#1-Co(2)-O(41)	84.88(12)
O(14)#1-Co(2)-O(43)	102.41(11)
O(14)#1-Co(2)-O(40)	169.91(11)
O(15)#1-Co(2)-Co(5)	137.74(16)
O(15)#1-Co(2)-O(41)	88.97(18)
O(15)#1-Co(2)-O(43)	100.96(18)
O(15)#1-Co(2)-O(40)	91.25(17)
O(15)#1-Co(2)-O(14)#1	81.01(18)

Symmetry transformations used to generate equivalent atoms: #1 -y+1,x-1,z #2 y+1,-x+1,z #3 -x+2,-y,z

Supplementary Table 5. Optimization of the reaction conditions for [3+2] coupling reaction

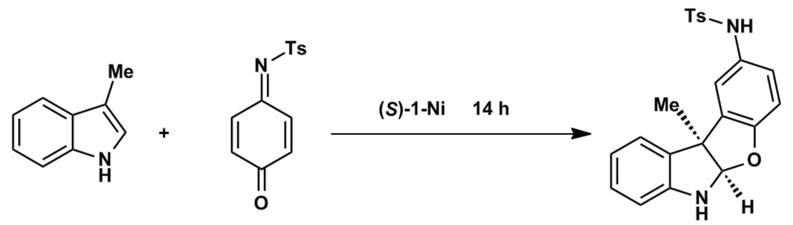

Entry	Solvent	catalyst loading (*x* mol%)	T (° C)	yield (%)[a]	e.e. (%)[b]
1	MeCN	0.5	RT	96	91
2	THF	0.5	RT	90	88
3	DCM	0.5	RT	86	79
4	$CHCl_3$	0.5	RT	88	82
5	Ph-CH_3	0.5	RT	95	84
6	EA	0.5	RT	66	70
7	acetone	0.5	RT	75	66
8	H_2O	0.5	RT	82	0
9	MeCN	0.5	0	94	99
10	MeCN	0.1	0	92	99
11	MeCN	0.05	0	92	99
12	MeCN[c]	0.02	0	72	94
13	MeCN	0.04	0	92	99

[a] Isolated yield. [b] Determined by HPLC. [c] Reaction time: 30 h.

Supplementary Table 6. Optimization of the reaction conditions for Friedel-Crafts reaction

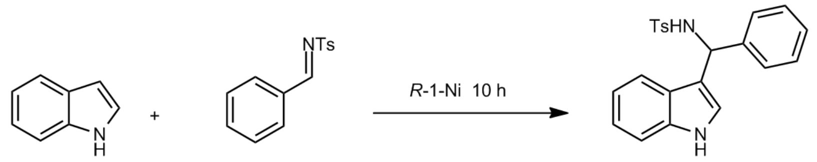

entry	solvent	catalyst loading (*x* mol%)	T (° C)	yield (%)[a]	ee. (%)[b]
1	DCM	0.04	RT	93	82
2	DCE	0.04	RT	94	85
3	Ph-CH_3	0.04	RT	88	80
4	THF	0.04	RT	64	65
5	$CHCl_3$	0.04	RT	82	76
6	H_2O	0.04	RT	0	n.d.
7	DCE	0.04	0	92	91
8	DCE[c]	0.02	0	80	88

[a] Isolated yield. [b] Determined by HPLC. [c] Reaction time: 24 h.

Supplementary Table 7. Catalytic activities of reported related homogeneous and heterogeneous catalysts in F-C reaction

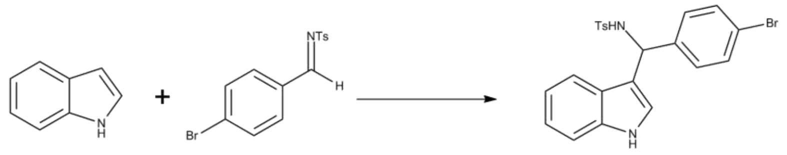

Entry	Cat.	Temp (° C)	Yield (%)	ee (%)	
1	Meso-silica/9-thiourea epi-quinine [9]	40	68	90	Hetero
2	Polystyrene-supported phosphoric acid [10]	RT	86	87	Hetero
3	SPINOL-Based Phosphoric Acid [11]	0	99	99	Homo
4	BINOL-Based Phosphoric Acid [12]	-60	71	82	Homo
5	BINOL-Sulfonimide [13]	-40	60	88	Homo
6	bisoxazoline/Cu [14]	20	89	96	Homo
7	Schiff base/Cu [15]	20	90	95	Homo

Supplementary Table 8. Recycling tests of the 1-Ni catalyst in Friedel-Crafts reaction

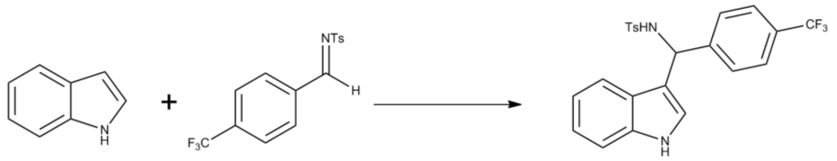

Run	1	2	3	4	5	6	7	8	9	10
Yield (%)	96	96	97	94	95	93	91	90	92	90
ee (%)	96	95	96	94	94	95	96	94	94	95

Supplementary Figures

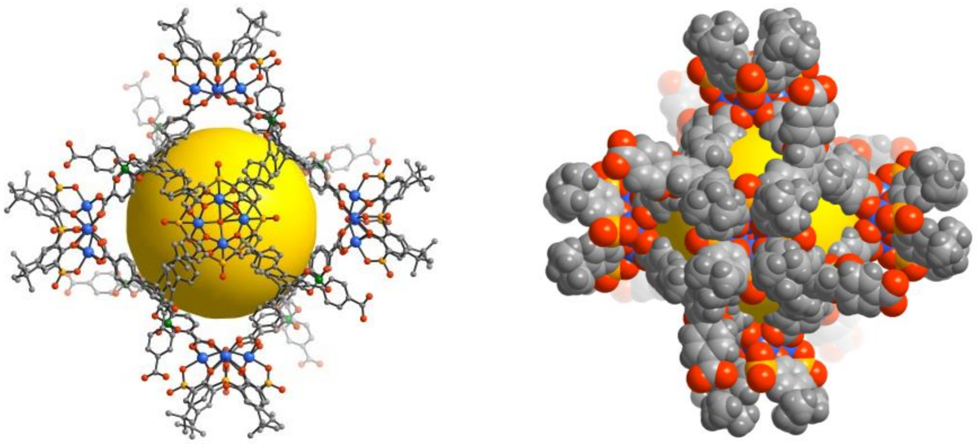

Supplementary Figure 1. Crystal structures. left: A single octahedral cage of **1**-Ni viewed along the long axis; **right**: Space filling model of **1**-Ni

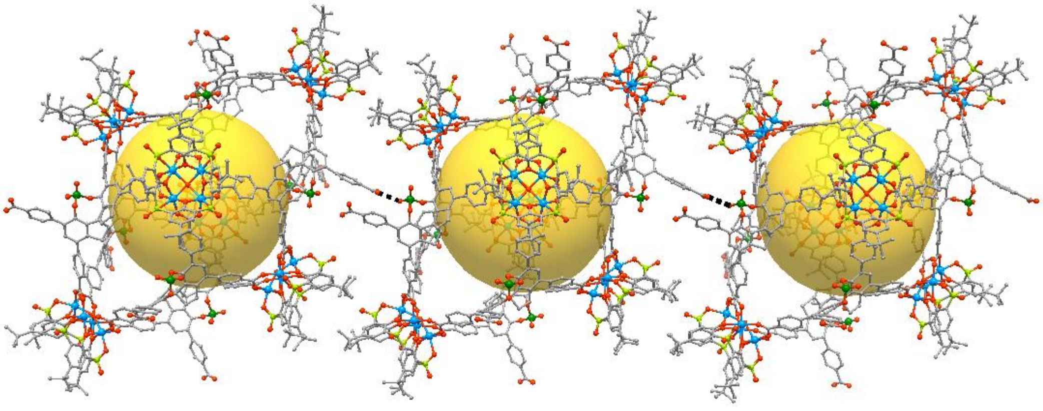

Supplementary Figure 2. Crystal structure. View of the 1D chain formed by adjacent cages in **1**-Ni; (b) The 2D net formed by octahedral cages in **1**-Ni.

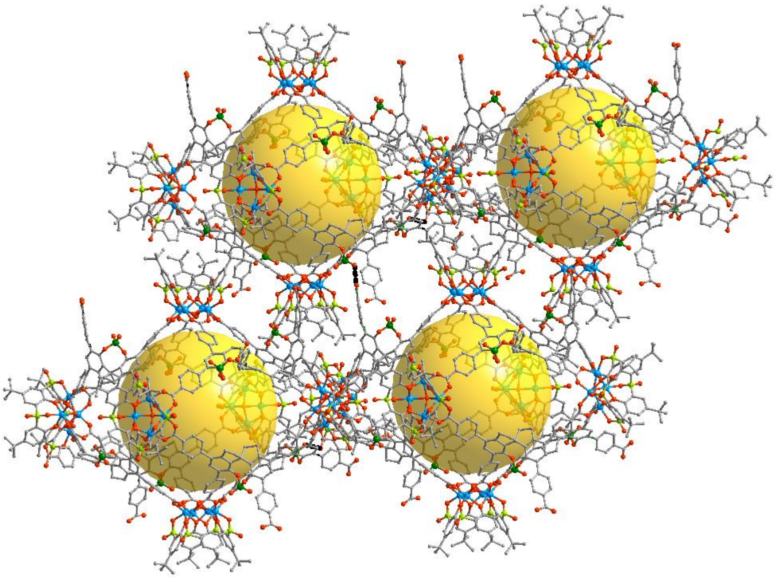

Supplementary Figure 3. Crystal structure. View of the 2D net formed by adjacent cages in **1**-Ni

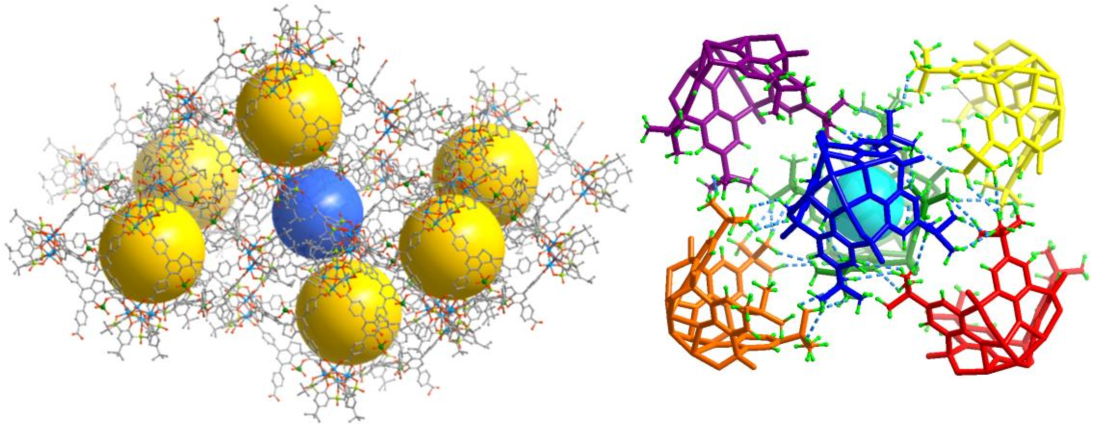

Supplementary Figure 4. Crystal structure. left: View of the hydrophobic cavity formed by six octahedral cages in **1**-Ni. **right**: View of the hydrophobic cavity formed by six TBSC moieties from six cages in **1**-Ni.

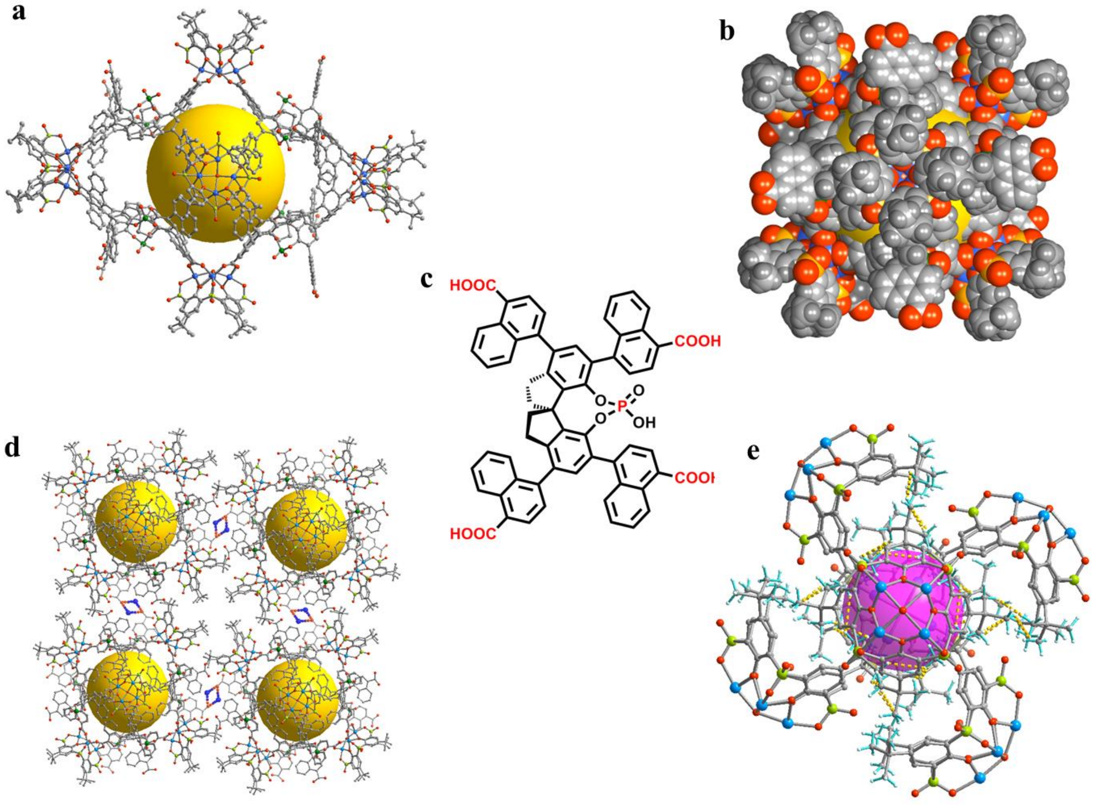

Supplementary Figure 5. Chemical and Crystal structure. (a) A single octahedral cage of **1**-Co’ viewed along the short axis. (b) Space filling model of **1**-Co’along the c axis. (c) The chemical structure of the expanded ligand. (d) Packing structure along the c axis showing the O-H…H intercage hydrogen bonds in **1**-Co’. (e) Packing structure through six $TBSC^{4-}$ units along the c axis showing the C-H…H-C interactions in **1**-Co’.

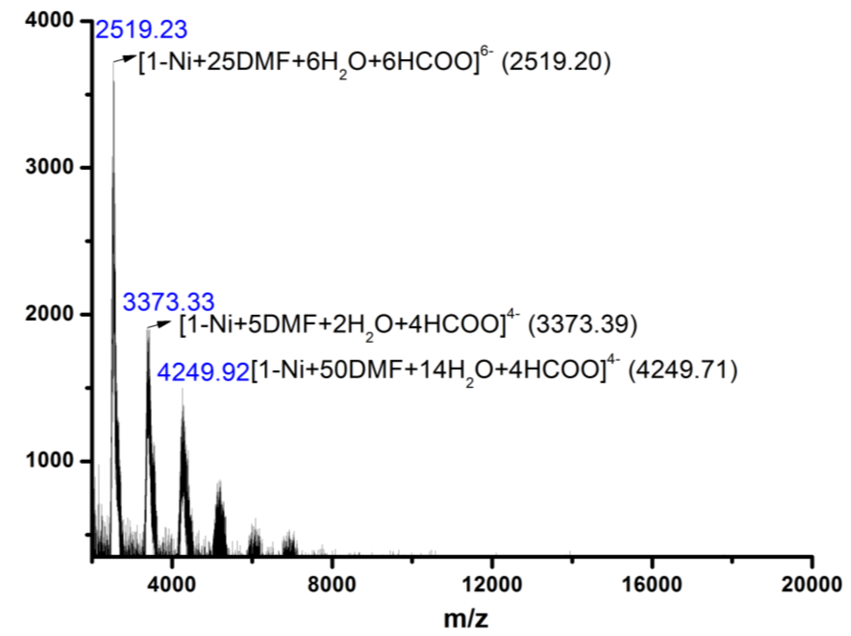

Supplementary Figure 6. Q-TOF-MS. Quadrupole-time-offlight mass spectrometry of **1**-Ni*

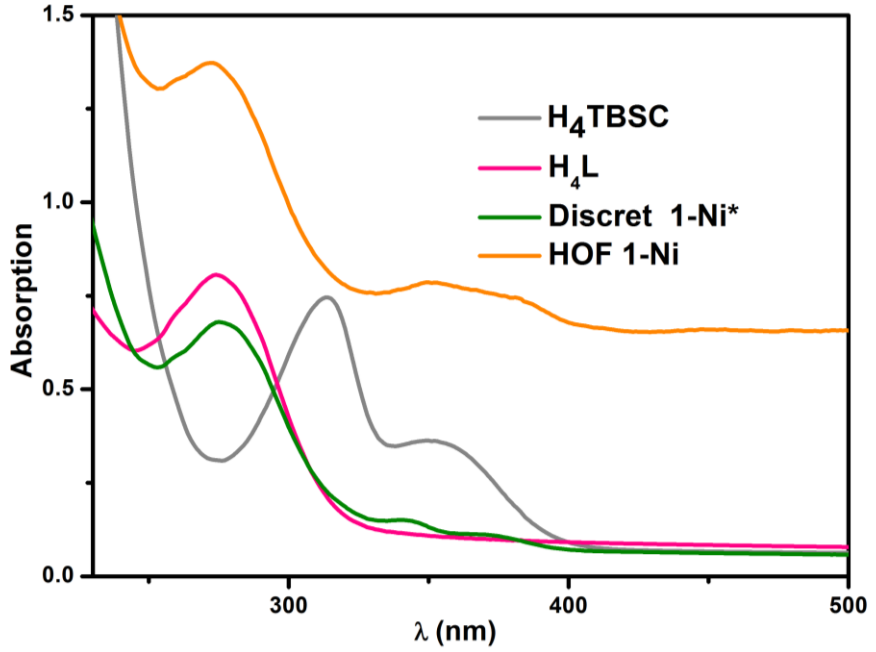

Supplementary Figure 7. UV-vis spectra. UV-vis spectra of ligand H_4TBSC, H_4**L** and discret cage **1**-Ni* and HOF **1**-Ni

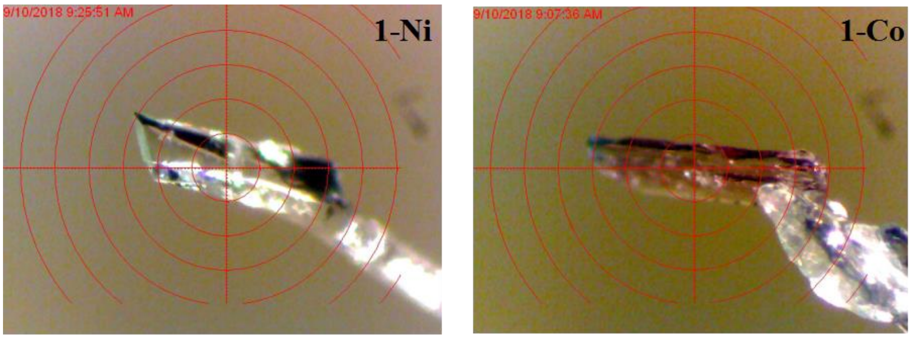

Supplementary Figure 8. Crystal photographs. Microscopical crystal photographs of 1-Ni and 1-Co

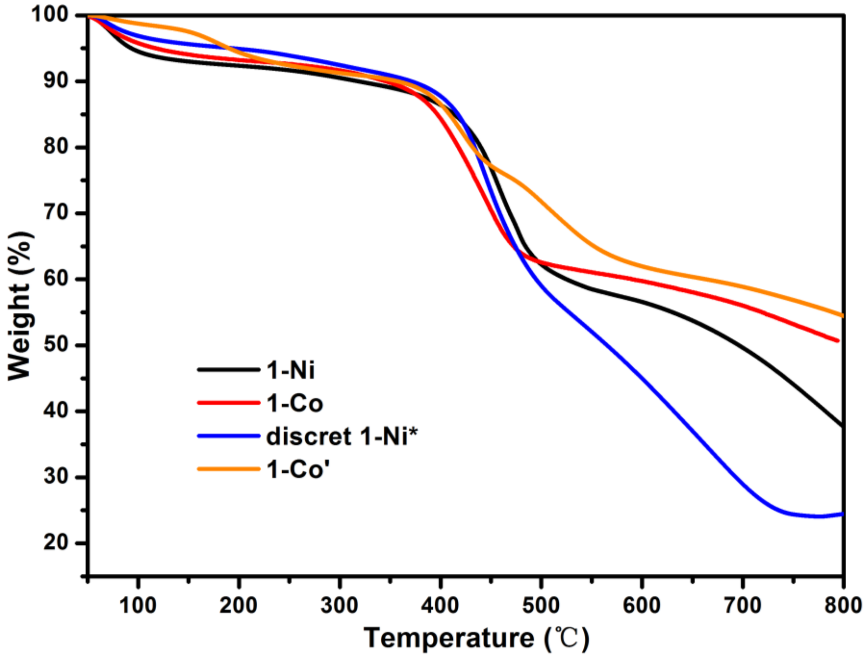

Supplementary Figure 9. TGA curves. TGA curves of HOFs **1**-Ni, **1**-Co, **1**-Co’ and discret cage **1**-Ni*

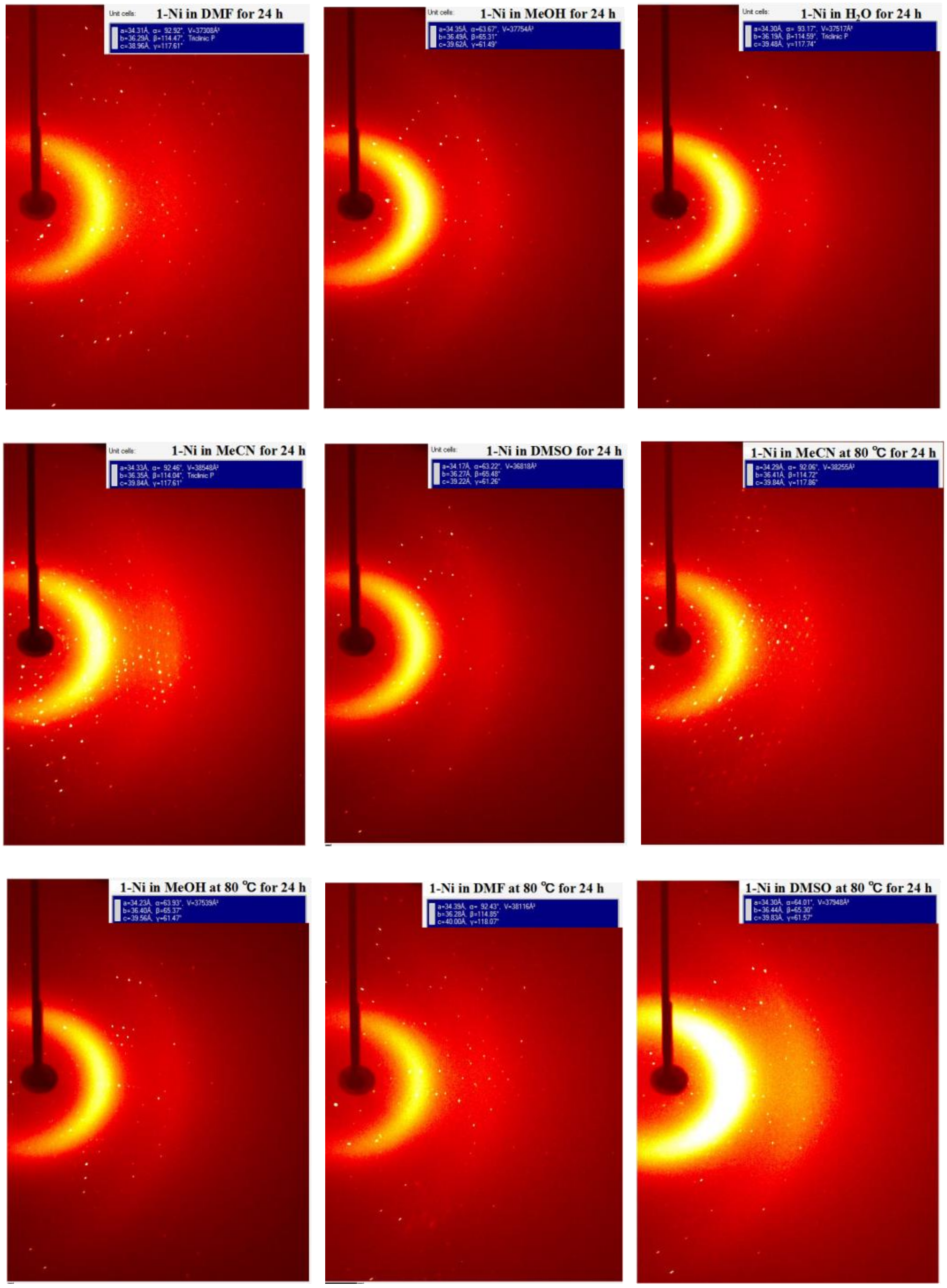

Supplementary Figure 10. Diffraction pictures. Single-crystal X-ray diffraction and unit cell determination of **1**-Ni after different treatments

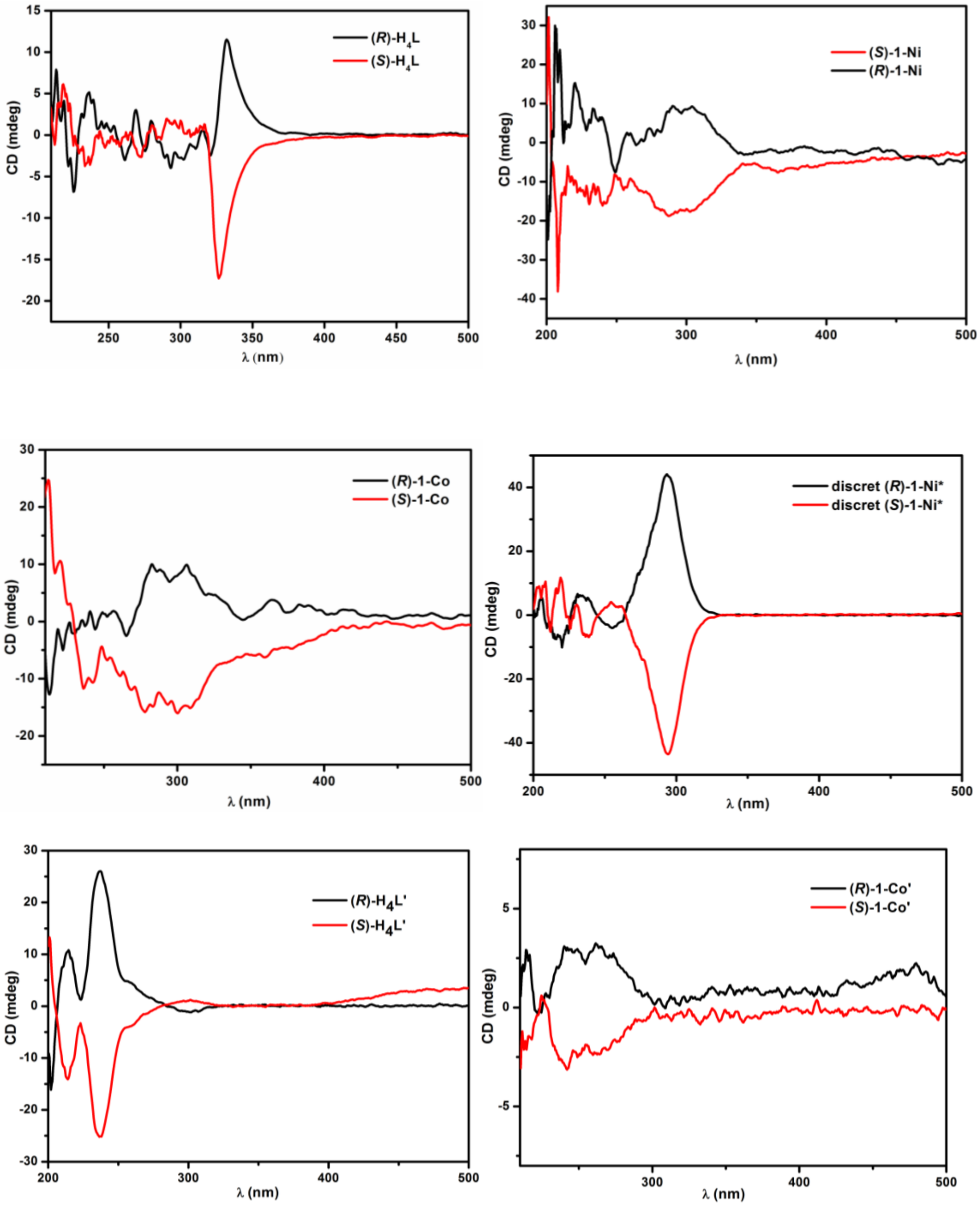

Supplementary Figure 11. CD spectra. CD spectra of ligands, HOFs and discret cages

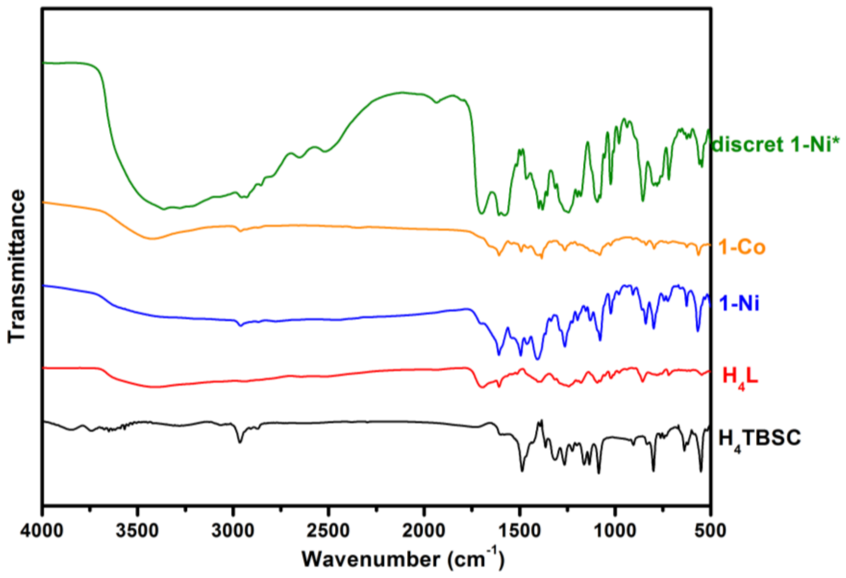

Supplementary Figure 12. IR spectra. IR spectra of ligands H_4TBSC, H_4**L** and HOFs **1**-Ni, **1**-Co and discret cage **1**-Ni*

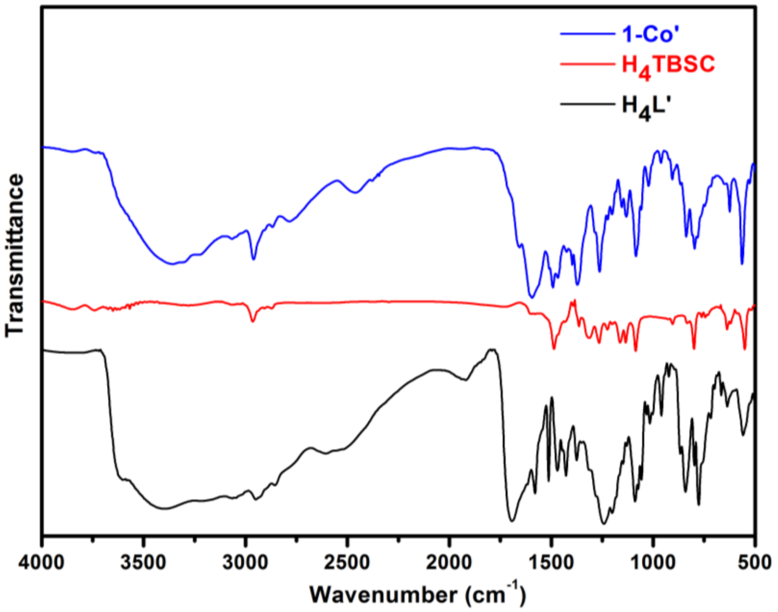

Supplementary Figure 13. IR spectra. IR spectra of ligands H_4TBSC, H_4**L'** and HOF**1**-Co'

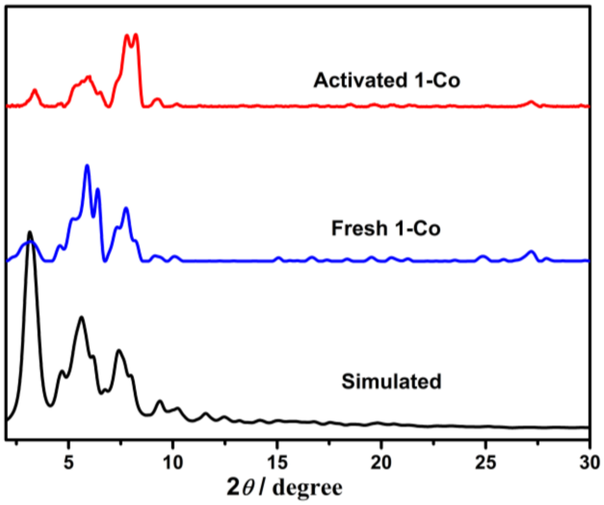

Supplementary Figure 14. PXRD patterns. PXRD patterns of HOF **1**-Co

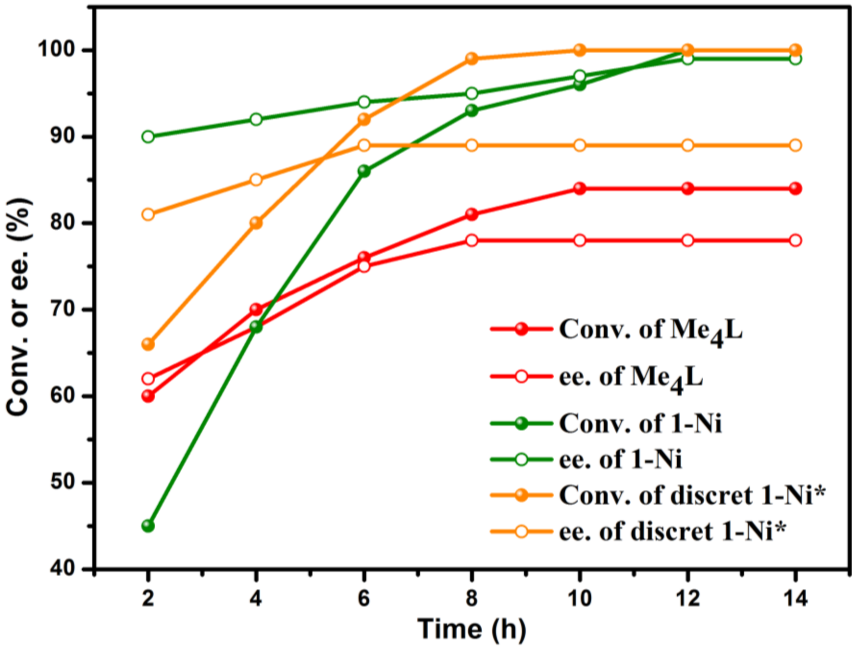

Supplementary Figure 15. Kinetic curves. Kinetic study of asymmetric [3+2] coupling of 3-methyl indole with quinone monoimine

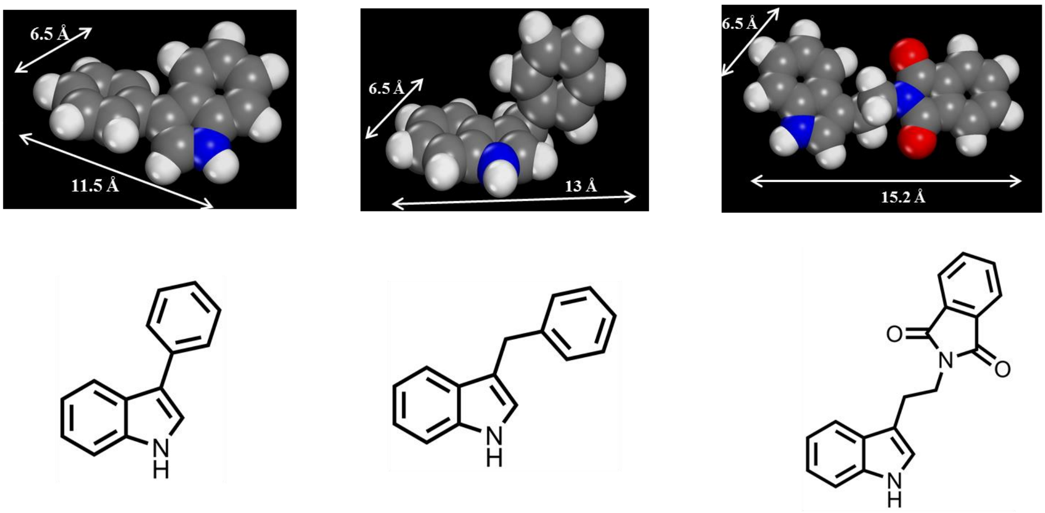

3-phenyl indole **3-benzyl indole** **3-(2-phthalimidoethyl)-indole**

Supplementary Figure 16. Molecular simulations. Molecular mechanics simulation results of three large substrates. The space-filling models were calculated by Materials Studio (7.0), and were fully optimized using MS Forcite molecular dynamics module (universal force fields, Ewald summations) method.

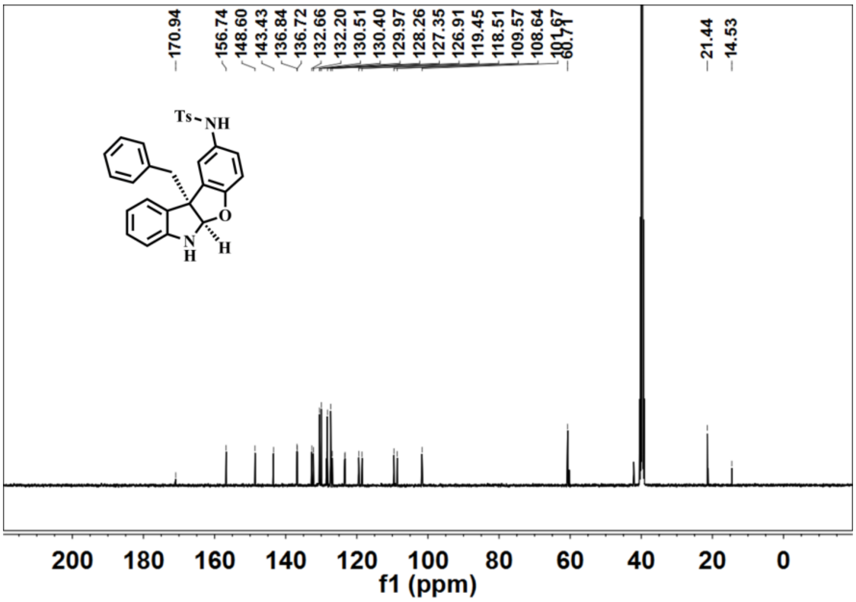

^{13}C NMR of product of [3+2] coupling reaction of 3-benzyl indole with quinone monoimine in DMSO-d6

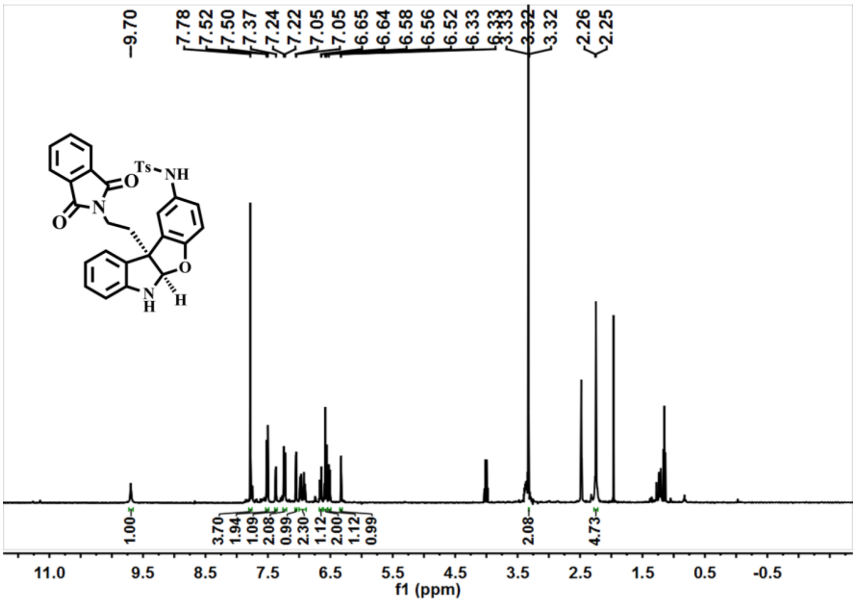

^{1}H NMR of product of [3+2] coupling reaction of 3-(2-phthalimidoethyl)-indole with quinone monoimine in DMSO-d6

HPLC and NMR copies of [3+2] coupling of 3-substituted indoles with quinone monoimine

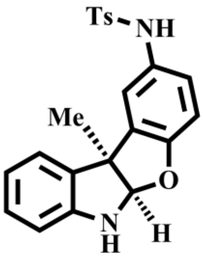

Enantiomeric excess was determined by HPLC with a chiralcel OD-H column (hexane/iPrOH = 70/30, 1.0 mL/min), t_{major} = 9.27 min, t_{minor} = 13.85 min; ee = 99.9 %. ^{1}H NMR (400 MHz, $CDCl_3$) δ: 7.52 (d, *J* = 8.3 Hz, 2H), 7.16 (d, *J* = 8.3 Hz, 2H), 7.09 – 7.02 (m, 3H), 6.77 (t, *J* = 7.4 Hz, 1H), 6.71 – 6.63 (m, 2H), 6.58 (d, *J* = 8.4 Hz, 1H), 6.44 (s, 1H), 6.04 (d, *J* = 2.5 Hz, 1H), 5.01 (s, 1H), 2.37 (s, 3H), 1.60 (s, 3H).

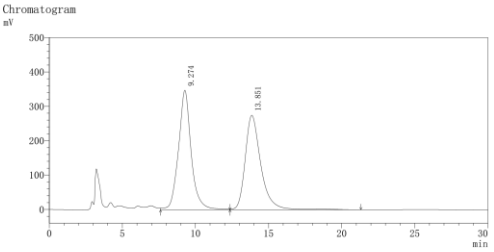

230nm

ID#	Start	End	Ret. Time	Height	Area	Area%
1	7.608	12.358	9.274	347762	21126624	50.564
2	12.358	21.317	13.851	274879	20655630	49.436
				622641	41782254	100.000

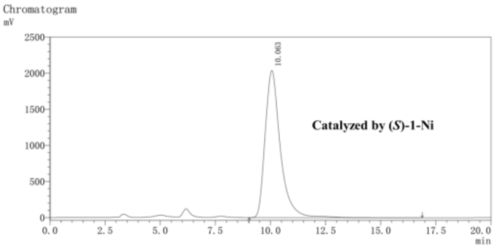

ID#	Start	End	Ret. Time	Height	Area	Area%
1	9.033	16.883	10.063	2038709	97562043	100.000
				2038709	97562043	100.000

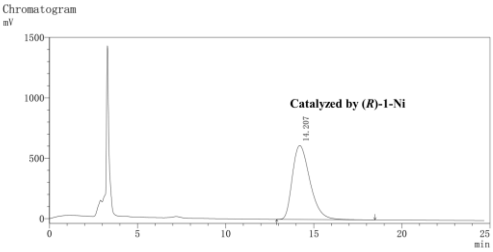

230nm

ID#	Start	End	Ret. Time	Height	Area	Area%
1	12.900	18.475	14.207	612467	42469812	100.000
				612467	42469812	100.000

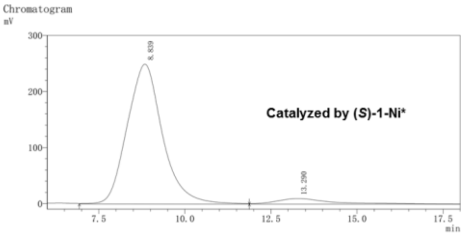

254nm

ID#	Start	End	Ret. Time	Height	Area	Area%
1	6.933	11.875	8.839	249637	17853089	94.160
2	11.875	18.833	13.290	9845	1107359	5.840
				259481	18960449	100.000

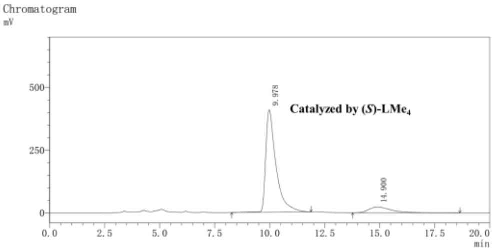

254nm

ID#	Start	End	Ret. Time	Height	Area	Area%
1	8.275	11.875	9.978	407888	13143711	89.020
2	13.775	18.650	14.900	22797	1621260	10.980
				430686	14764971	100.000

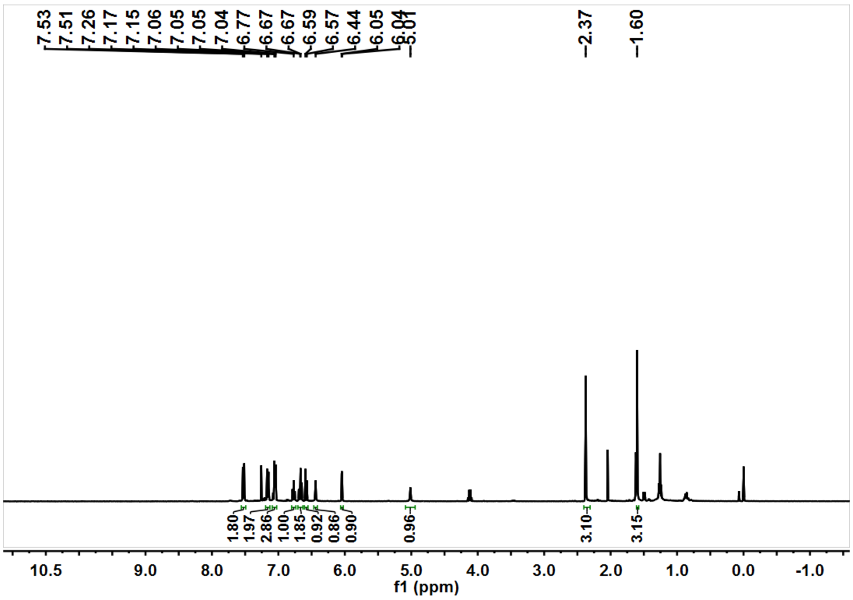

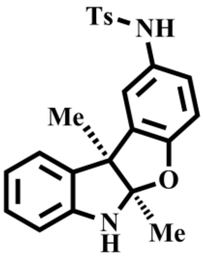

Enantiomeric excess was determined by HPLC with a chiralcel AD-H column (hexane/iPrOH = 60/40, 1.0 mL/min), t_{major} = 11.35 min, t_{minor} = 35.98 min; ee = 95%. ^{1}H NMR (400 MHz, $CDCl_3$) δ: 7.54 (d, *J* = 8.2 Hz, 2H), 7.16 (d, *J* = 8.1 Hz, 2H), 7.03 (dd, *J* = 14.9, 4.9 Hz, 2H), 6.96 (d, *J* = 7.4 Hz, 1H), 6.74 (t, *J* = 7.4 Hz, 1H), 6.71 – 6.58 (m, 3H), 6.52 (d, *J* = 8.3 Hz, 1H), 4.84 (s, 1H), 2.38 (s, 3H), 1.62 (s, 3H), 1.49 (s, 3H).

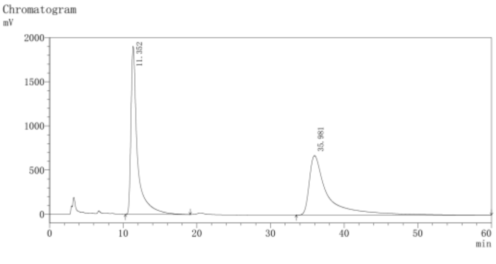

230nm

ID#	Start	End	Ret. Time	Height	Area	Area%
1	10.292	19.092	11.352	1903080	116300807	49.846
2	33.492	60.000	35.981	671959	117017370	50.154
				2575039	233318177	100.000

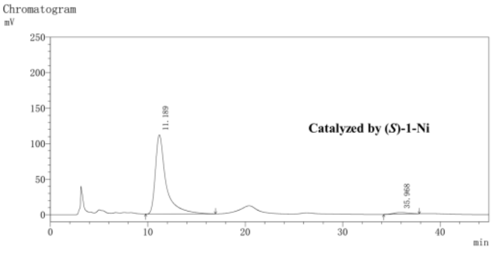

254nm

ID#	Start	End	Ret. Time	Height	Area	Area%
1	9.775	16.942	11.189	111533	8480484	97.274
2	34.208	37.867	35.968	2263	237649	2.726
				113796	8718133	100.000

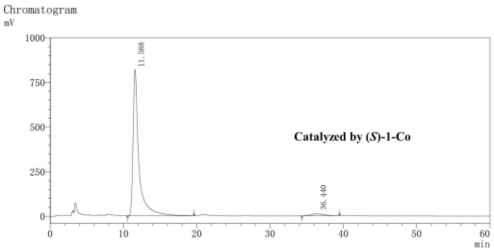

230nm

ID#	Start	End	Ret. Time	Height	Area	Area%
1	10.558	19.633	11.588	817859	44891904	97.166
2	34.367	39.467	36.440	10629	1309279	2.834
				828489	46201184	100.000

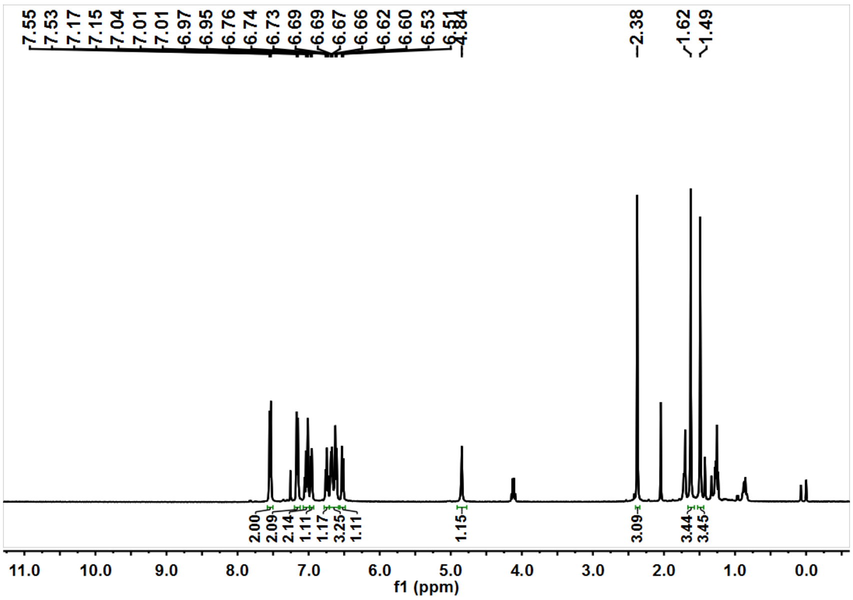

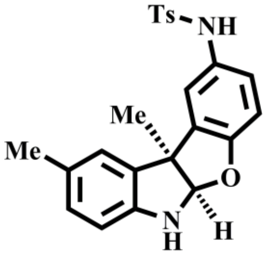

Enantiomeric excess was determined by HPLC with a chiralcel AD-H column (hexane/iPrOH = 60/40, 1.0 mL/min), t_{major} = 11.74 min, t_{minor} = 26.80 min; ee = 94%. ^{1}H NMR (400 MHz, $CDCl_3$) δ: 7.58 (d, *J* = 8.3 Hz, 2H), 7.20 (d, *J* = 8.1 Hz, 2H), 7.15 (d, *J* = 2.2 Hz, 1H), 6.95 (s, 1H), 6.90 (d, *J* = 7.9 Hz, 1H), 6.66 (dd, *J* = 8.4, 2.2 Hz, 1H), 6.59 (dd, *J* = 8.1, 2.0 Hz, 2H), 6.42 (s, 1H), 6.06 (d, *J* = 2.6 Hz, 1H), 4.93 (d, *J* = 1.8 Hz, 1H), 2.39 (s, 3H), 2.28 (s, 3H), 1.62 (s, 3H).

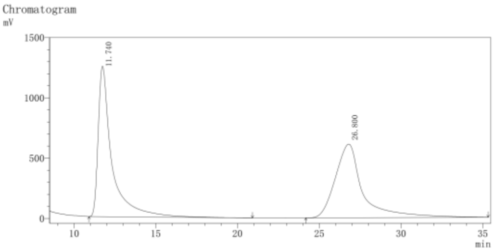

230nm

ID#	Start	End	Ret. Time	Height	Area	Area%
1	10.917	20.917	11.740	1247787	73171459	50.565
2	24.175	35.333	26.800	611085	71537327	49.435
				1858872	144708785	100.000

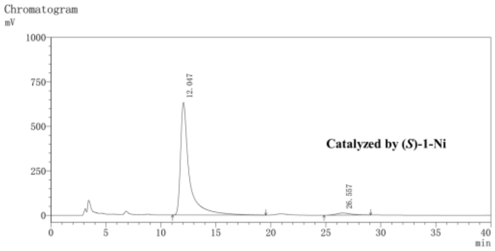

230nm

ID#	Start	End	Ret. Time	Height	Area	Area%
1	11.058	19.550	12.047	632466	34849913	97.007
2	24.833	29.075	26.557	11659	1075137	2.993
				644126	35925050	100.000

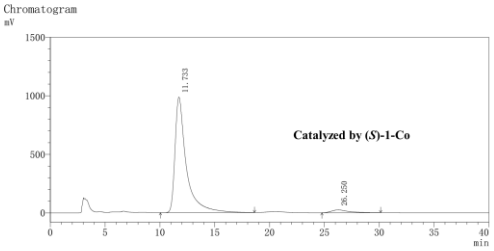

230nm

ID#	Start	End	Ret. Time	Height	Area	Area%
1	10.050	18.625	11.733	987407	70540793	96.535
2	24.775	30.125	26.250	23851	2532182	3.465
				1011259	73072975	100.000

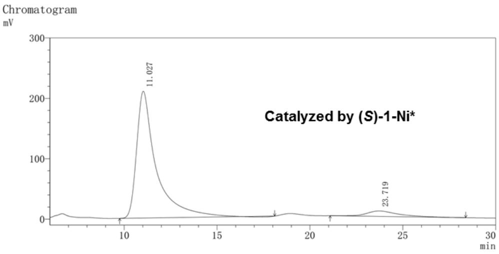

254nm

ID#	Start	End	Ret. Time	Height	Area	Area%
1	9.758	18.100	11.027	210067	15383104	93.249
2	21.083	28.383	23.719	9047	1113623	6.751
				219114	16496727	100.000

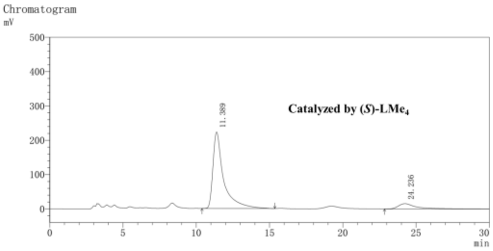

254nm

ID#	Start	End	Ret. Time	Height	Area	Area%
1	10.392	15.367	11.389	222635	11326374	88.837
2	22.850	31.167	24.236	15651	1423272	11.163
				238286	12749646	100.000

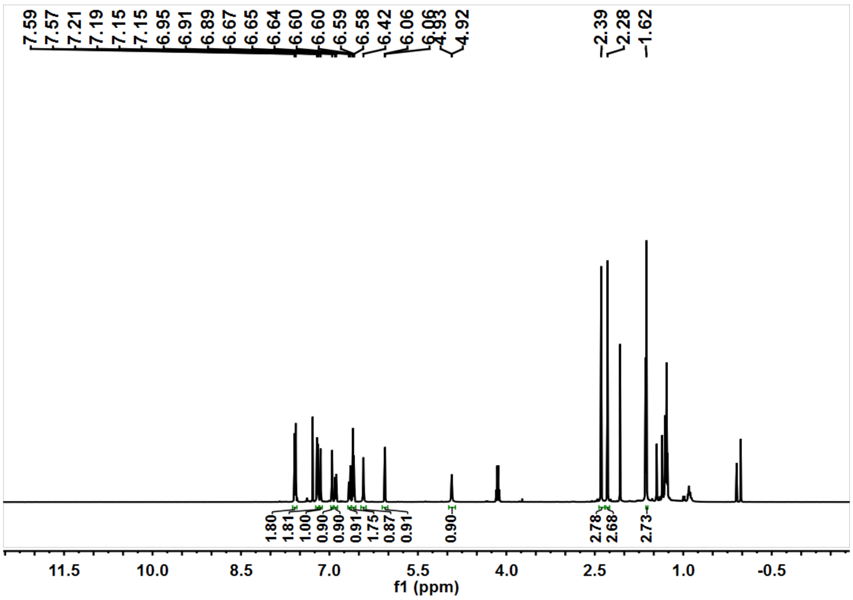

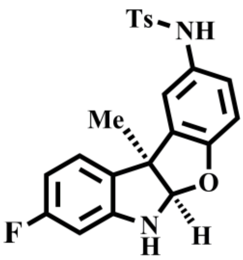

Enantiomeric excess was determined by HPLC with a chiralcel AD-H column (hexane/iPrOH = 60/40, 1.0 mL/min), t_{major} = 11.56 min, t_{minor} = 38.63 min; ee = 95%. ^{1}H NMR (400 MHz, $CDCl_3$) δ 7.53 (d, *J* = 8.3 Hz, 2H), 7.16 (d, *J* = 8.0 Hz, 2H), 7.04 (d, *J* = 2.2 Hz, 1H), 6.94 (dd, *J* = 8.1, 5.4 Hz, 1H), 6.69 (dd, *J* = 8.5, 2.2 Hz, 1H), 6.62 – 6.52 (m, 2H), 6.46 – 6.38 (m, 1H), 6.34 (dd, *J* = 9.5, 2.2 Hz, 1H), 6.05 (d, *J* = 2.3 Hz, 1H), 5.10 (s, 1H), 2.37 (s, 3H), 1.58 (s, 3H).

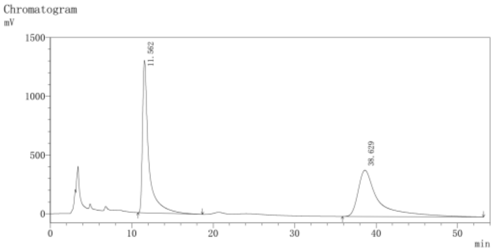

230nm

ID#	Start	End	Ret. Time	Height	Area	Area%
1	10.758	18.692	11.562	1297782	69446571	50.158
2	35.875	53.200	38.629	394456	69009003	49.842
				1692238	138455574	100.000

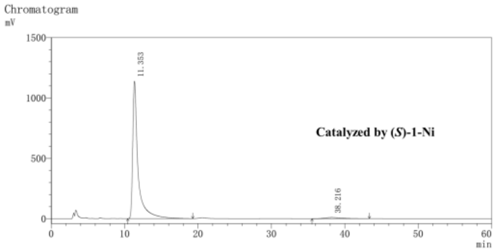

230nm

ID#	Start	End	Ret. Time	Height	Area	Area%
1	10.425	19.275	11.353	1137433	57479596	97.335
2	35.533	43.342	38.216	10226	1573876	2.665
				1147659	59053472	100.000

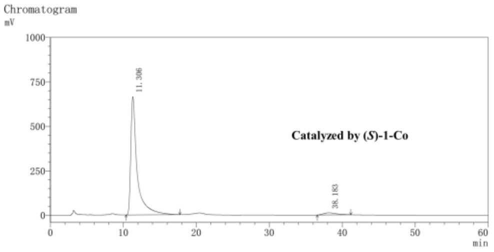

254nm

ID#	Start	End	Ret. Time	Height	Area	Area%
1	10.358	17.750	11.306	664946	40535575	96.516
2	36.575	41.192	38.183	11771	1463326	3.484
				676717	41998901	100.000

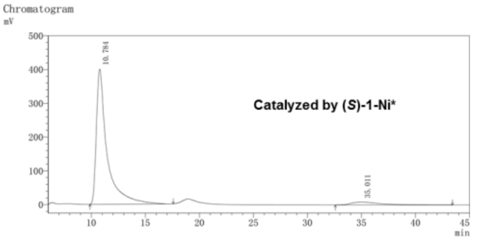

254nm

ID#	Start	End	Ret. Time	Height	Area	Area%
1	9.875	17.583	10.784	400273	27456883	94.306
2	32.575	43.417	35.011	8904	1657917	5.694
				409177	29114801	100.000

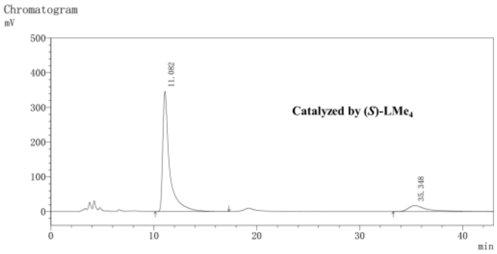

254nm

ID#	Start	End	Ret. Time	Height	Area	Area%
1	10.150	17.300	11.082	345866	15889752	87.632
2	33.242	43.517	35.348	17443	2242582	12.368
				363309	18132334	100.000

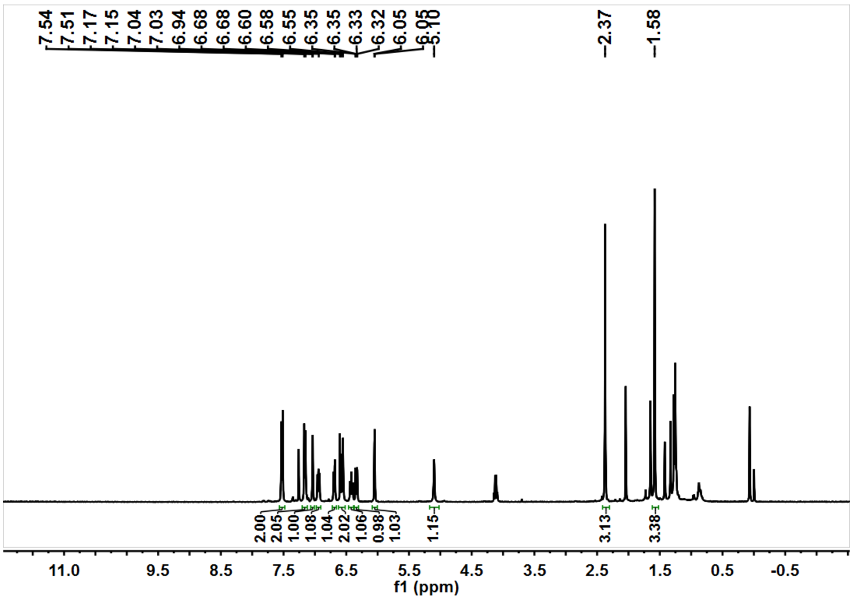

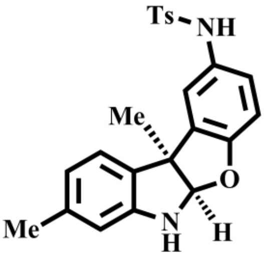

Enantiomeric excess was determined by HPLC with a chiralcel AD-H column (hexane/iPrOH = 60/40, 1.0 mL/min), t_{major} = 15.36 min, t_{minor} = 26.52 min; ee = 99.9%. ^{1}H NMR (400 MHz, $CDCl_3$) δ: 7.52 (d, *J* = 8.2 Hz, 2H), 7.17 (d, *J* = 8.0 Hz, 2H), 7.02 (d, *J* = 2.1 Hz, 1H), 6.93 (d, *J* = 7.6 Hz, 1H), 6.69 – 6.63 (m, 1H), 6.58 (t, *J* = 7.0 Hz, 2H), 6.49 (s, 1H), 6.32 (s, 1H), 6.03 (d, *J* = 2.4 Hz, 1H), 4.94 (s, 1H), 2.38 (s, 3H), 2.26 (s, 3H), 1.58 (s, 3H).

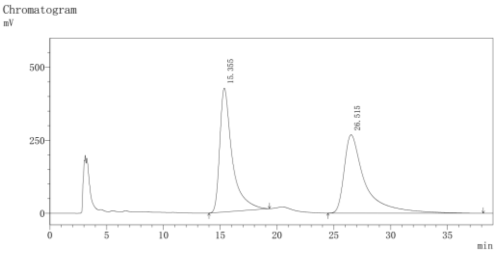

230nm

ID#	Start	End	Ret. Time	Height	Area	Area%
1	14.008	19.325	15.355	424309	32395332	48.431
2	24.475	38.150	26.515	268429	34494420	51.569
				692738	66889752	100.000

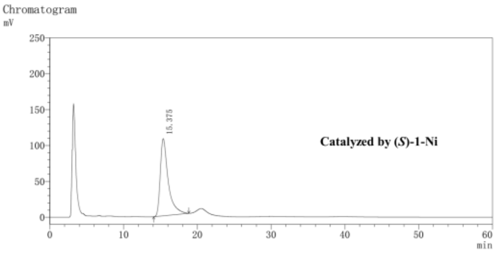

230nm

ID#	Start	End	Ret. Time	Height	Area	Area%
1	14.075	18.850	15.375	107666	8041499	100.000
				107666	8041499	100.000

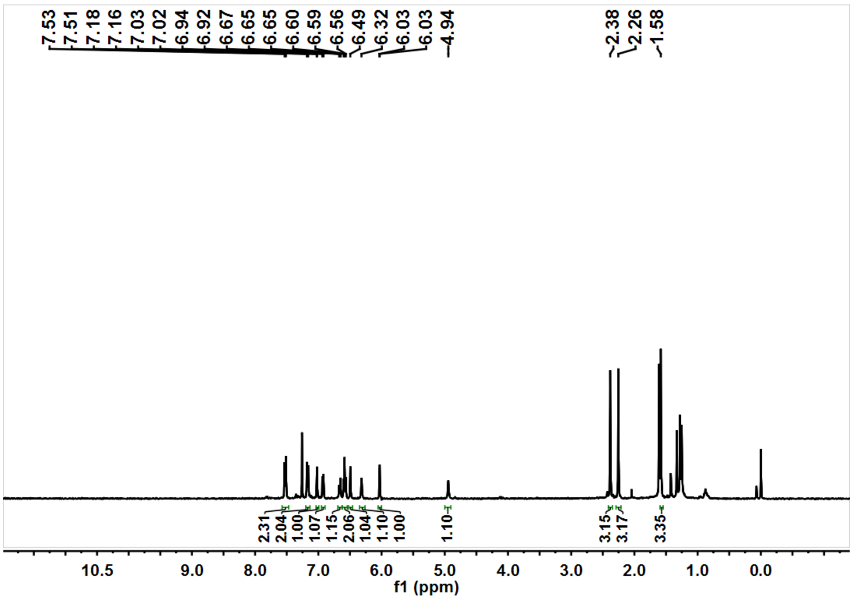

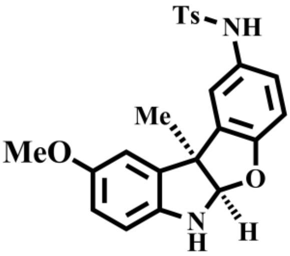

Enantiomeric excess was determined by HPLC with a chiralcel AD-H column (hexane/iPrOH = 60/40, 1.0 mL/min), t_{major} = 18.51 min, t_{minor} = 46.43 min; ee = 91%. ^{1}H NMR (400 MHz, $CDCl_3$) δ: 7.52 (d, *J* = 7.8 Hz, 2H), 7.18 (d, *J* = 7.9 Hz, 2H), 7.05 (s, 1H), 6.72 – 6.56 (m, 5H), 6.20 (s, 1H), 6.04 (s, 1H), 5.30 (s, 1H), 3.75 (s, 3H), 2.37 (s, 3H), 1.60 (s, 3H).

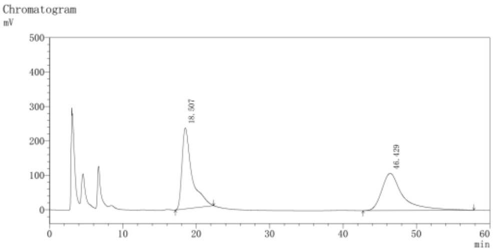

230nm

ID#	Start	End	Ret. Time	Height	Area	Area%
1	17.150	22.375	18.507	234863	22253991	50.832
2	42.725	57.850	46.429	107928	21525425	49.168
				342792	43779416	100.000

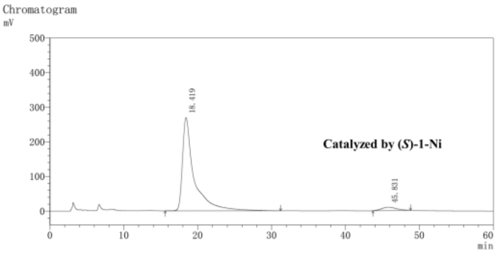

254nm

ID#	Start	End	Ret. Time	Height	Area	Area%
1	15.592	31.217	18.419	269311	29635231	95.639
2	43.708	48.808	45.831	9629	1351249	4.361
				278940	30986480	100.000

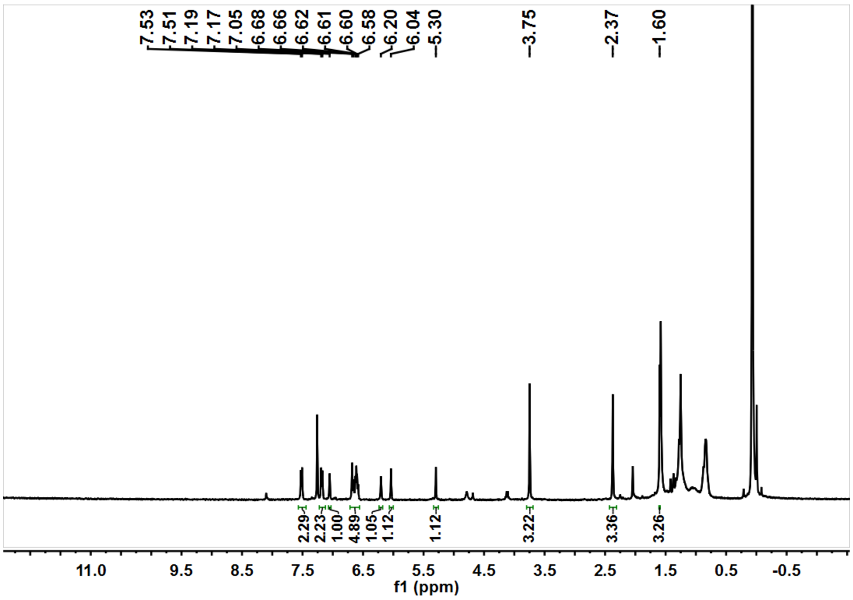

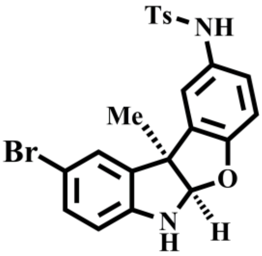

Enantiomeric excess was determined by HPLC with a chiralcel AD-H column (hexane/iPrOH = 60/40, 1.0 mL/min), t_{major} = 9.56 min, t_{minor} = 40.39 min; ee = 92%. ^{1}H NMR (400 MHz, $CDCl_3$) δ: 7.60 (d, *J* = 8.1 Hz, 2H), 7.20 (d, *J* = 8.0 Hz, 2H), 7.17 – 7.08 (m, 2H), 7.04 (d, *J* = 1.7 Hz, 1H), 6.97 (s, 1H), 6.76 (dd, *J* = 8.4, 1.7 Hz, 1H), 6.57 (d, *J* = 8.4 Hz, 1H), 6.50 (d, *J* = 8.2 Hz, 1H), 6.00 (d, *J* = 1.5 Hz, 1H), 5.14 (s, 1H), 2.36 (s, 3H), 1.55 (s, 3H).

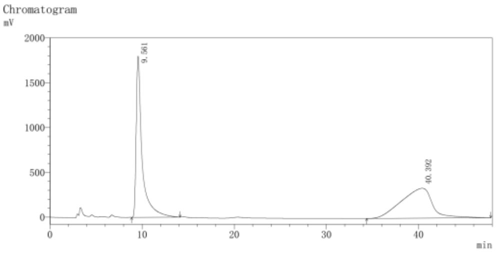

230nm

ID#	Start	End	Ret. Time	Height	Area	Area%
1	8.867	14.117	9.561	1798393	80596274	50.687
2	34.375	47.842	40.392	332977	78412108	49.313
				2131370	159008382	100.000

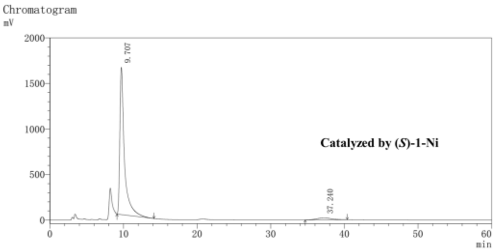

230nm

ID#	Start	End	Ret. Time	Height	Area	Area%
1	9.108	14.133	9.707	1617615	70010382	95.962
2	34.675	40.392	37.240	21906	2946211	4.038
				1639522	72956592	100.000

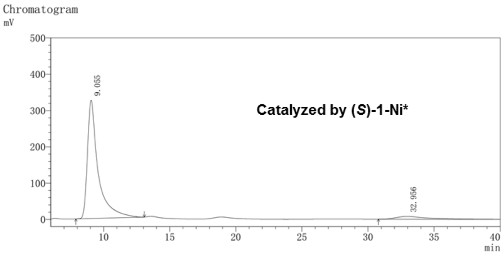

254nm

ID#	Start	End	Ret. Time	Height	Area	Area%
1	7.900	13.075	9.055	326163	18402130	93.565
2	30.783	40.392	32.956	7696	1265620	6.435
				333859	19667750	100.000

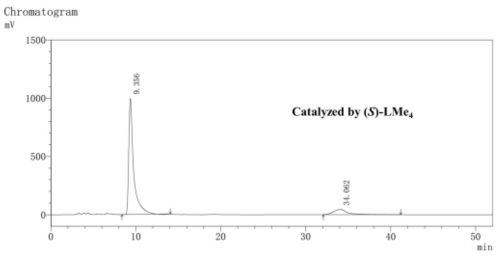

254nm

ID#	Start	End	Ret. Time	Height	Area	Area%
1	8.358	14.058	9.356	994117	39485784	88.214
2	32.067	41.183	34.062	44993	5275548	11.786
				1039109	44761332	100.000

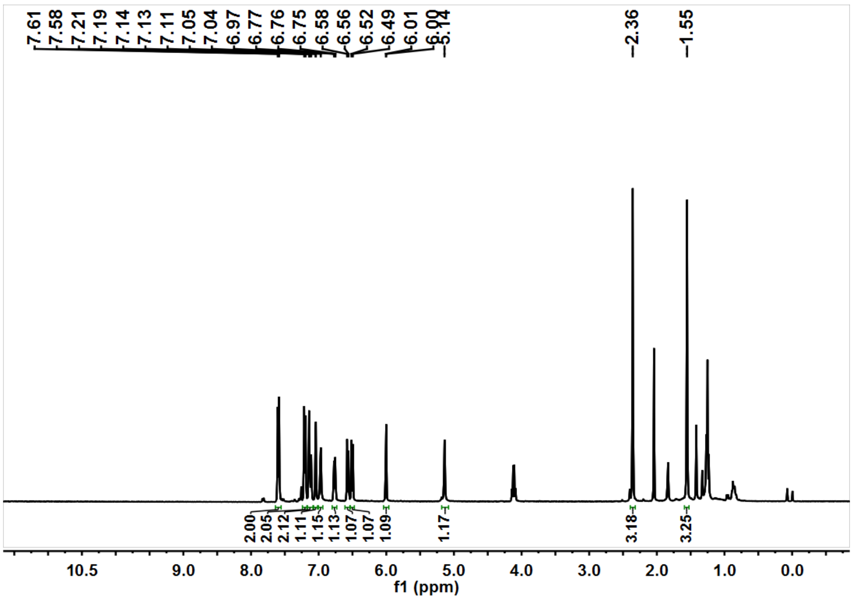

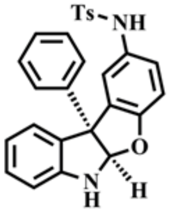

Enantiomeric excess was determined by HPLC with a chiralcel AD-H column (hexane/iPrOH = 60/40, 1.0 mL/min), t_{major} = 11.703 min, t_{minor} = 30.201 min; ee = 99.7%. ^{1}H NMR (400 MHz, DMSO-d^6) δ 9.74 (s, 1H), 7.51 (d, *J* = 8.2 Hz, 2H), 7.41 (s, 1H), 7.32 (d, *J* = 7.9 Hz, 5H), 7.09 (t, *J* = 7.3 Hz, 1H), 6.85 (dd, *J* = 13.3, 8.0 Hz, 5H), 6.76 – 6.65 (m, 3H), 6.06 (d, *J* = 2.1 Hz, 1H), 2.37 (s, 3H).

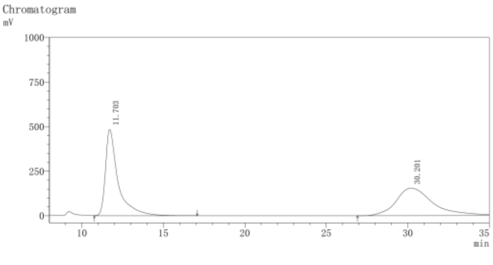

230nm

ID#	Start	End	Ret. Time	Height	Area	Area%
1	10.758	17.075	11.703	484028	26020554	51.082
2	26.908	37.675	30.201	154569	24918668	48.918
				638597	50939221	100.000

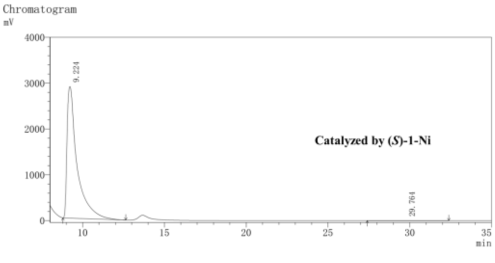

230nm

ID#	Start	End	Ret. Time	Height	Area	Area%
1	8.800	12.642	9.224	2872903	122215899	99.852
2	27.392	32.383	29.764	1604	181442	0.148
				2874507	122397340	100.000

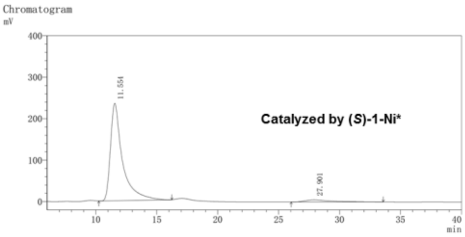

254nm

ID#	Start	End	Ret. Time	Height	Area	Area%
1	10.250	16.233	11.554	234842	16102704	95.440
2	26.025	33.583	27.901	5227	769371	4.560
				240069	16872075	100.000

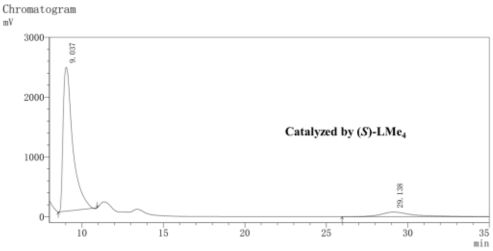

230nm

ID#	Start	End	Ret. Time	Height	Area	Area%
1	8.542	10.942	9.037	2407420	99198003	90.091
2	25.967	38.983	29.138	76386	10910484	9.909
				2483806	110108487	100.000

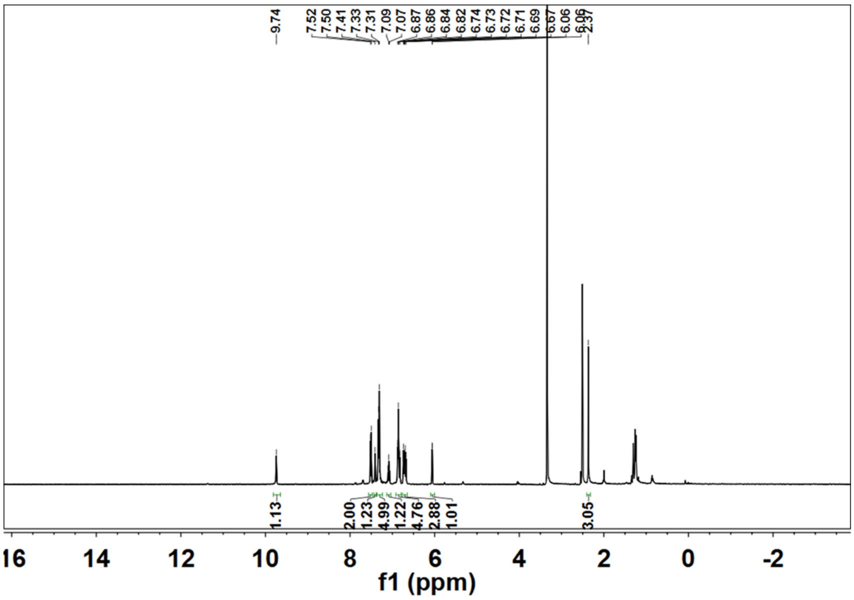

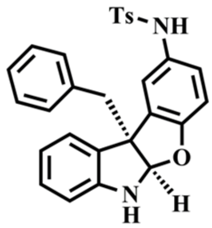

Enantiomeric excess was determined by HPLC with a chiralcel AD-H column (hexane/iPrOH = 60/40, 1.0 mL/min), t_{major} = 16.148 min, t_{minor} = 41.278 min; ee = 94%. ^{1}H NMR (400 MHz, DMSO) δ 9.81 (s, 1H), 7.59 (d, *J* = 8.3 Hz, 2H), 7.31 (dd, *J* = 10.8, 5.2 Hz, 3H), 7.23 (d, *J* = 7.2 Hz, 1H), 7.19 – 7.05 (m, 4H), 6.97 (td, *J* = 7.7, 1.1 Hz, 1H), 6.94 – 6.88 (m, 2H), 6.67 (td, *J* = 7.4, 0.7 Hz, 1H), 6.62 (dd, *J* = 8.5, 2.3 Hz, 1H), 6.47 (dd, *J* = 8.0, 5.4 Hz, 2H), 6.17 (d, *J* = 2.7 Hz, 1H), 3.36 (d, *J* = 13.6 Hz, 1H), 3.11 (d, *J* = 13.6 Hz, 1H), 2.33 (s, 3H).

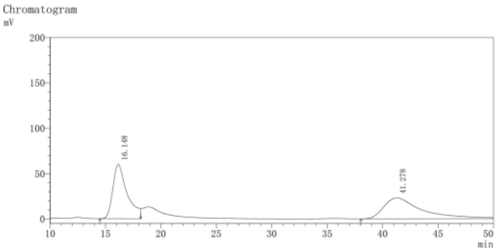

254nm

ID#	Start	End	Ret. Time	Height	Area	Area%
1	14.492	18.183	16.148	59896	5379024	48.773
2	38.033	55.175	41.278	23059	5649653	51.227
				82955	11028677	100.000

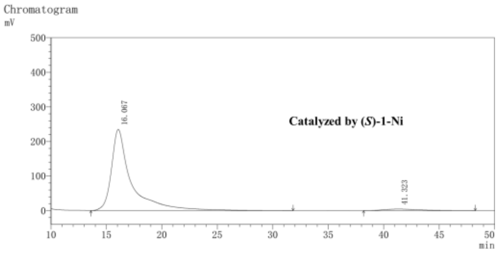

254nm

ID#	Start	End	Ret. Time	Height	Area	Area%
1	13.600	31.833	16.067	234838	29500400	96.741
2	38.183	48.267	41.323	4518	993735	3.259
				239356	30494135	100.000

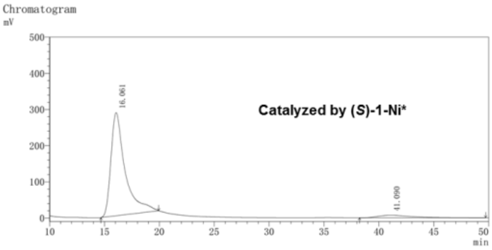

254nm

ID#	Start	End	Ret. Time	Height	Area	Area%
1	14.675	19.933	16.061	285156	25691476	94.369
2	38.233	49.725	41.090	7269	1533016	5.631
				292425	27224492	100.000

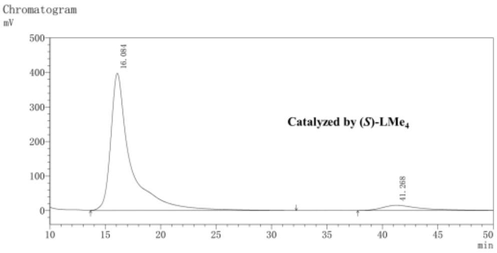

254nm

ID#	Start	End	Ret. Time	Height	Area	Area%
1	13.667	32.233	16.084	397527	49507604	93.306
2	37.783	52.025	41.268	14875	3551701	6.694
				412402	53059305	100.000

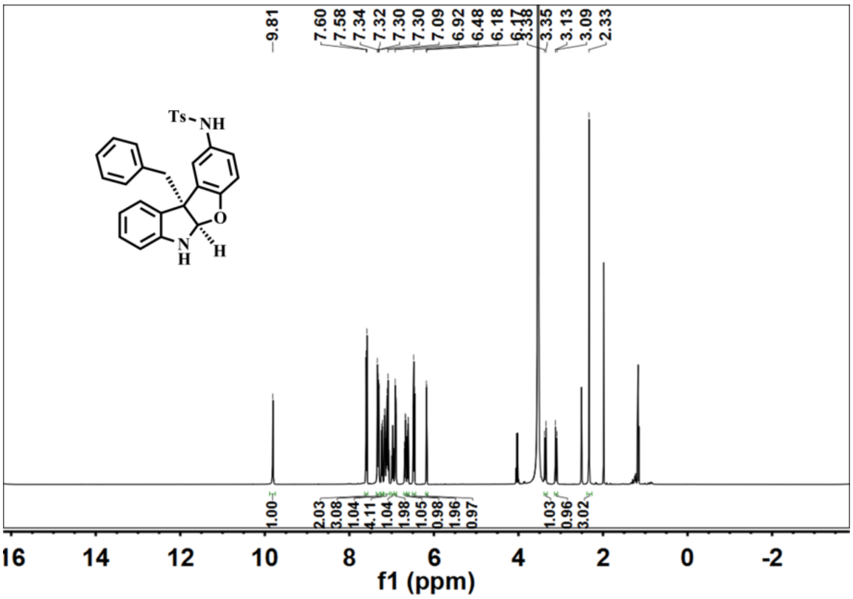

HPLC and NMR copies of Friedel-Crafts alkylation of indole with aryl aldimines

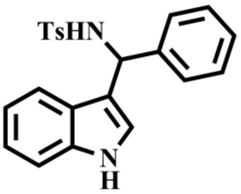

Enantiomeric excess was determined by HPLC with a chiralcel OD-H column (hexane/iPrOH = 70/30, 0.6 mL/min), t_{major} = 15.383 min, t_{minor} = 24.875 min; ee = 91%. ^{1}H NMR (400 MHz, DMSO-d_6) δ 10.85 (s, 1H), 8.47 (d, *J* = 8.9 Hz, 1H), 7.47 (d, *J* = 7.9 Hz, 2H), 7.31 – 7.20 (m, 4H), 7.19 – 7.07 (m, 5H), 7.01 (dd, *J* = 8.0, 7.2 Hz, 1H), 6.86 (dd, *J* = 7.9, 7.1 Hz, 1H), 6.75 (d, *J* = 2.4 Hz, 1H), 5.72 (d, *J* = 8.8 Hz, 1H), 2.26 (s, 3H).

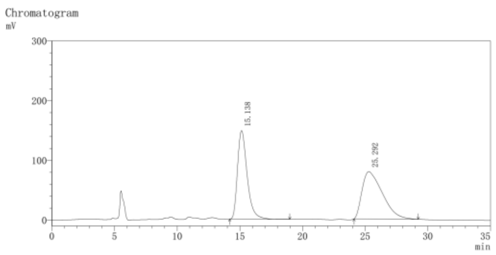

254nm

ID#	Start	End	Ret. Time	Height	Area	Area%
1	14.192	18.983	15.138	148280	7919993	47.043
2	24.108	29.242	25.292	79276	8915585	52.957
				227556	16835578	100.000

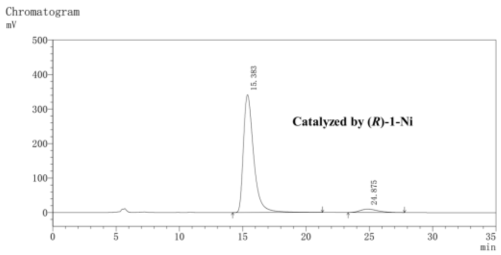

254nm

ID#	Start	End	Ret. Time	Height	Area	Area%
1	14.208	21.308	15.383	341341	19177438	95.363
2	23.325	27.783	24.875	9697	932561	4.637
				351038	20109999	100.000

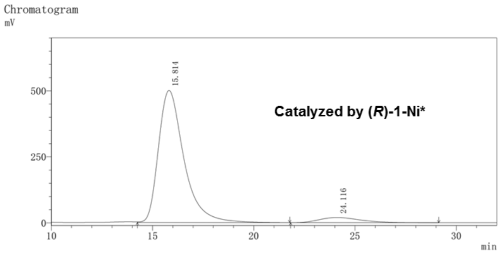

254nm

ID#	Start	End	Ret. Time	Height	Area	Area%
1	14.250	21.775	15.814	499559	43714373	94.092
2	21.817	29.133	24.116	19538	2744958	5.908
				519097	46459331	100.000

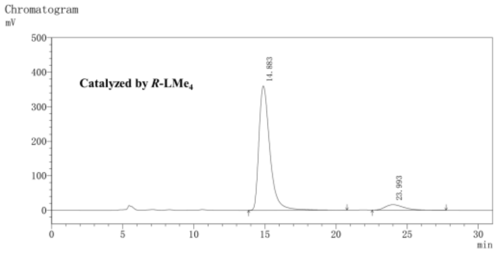

254nm

ID#	Start	End	Ret. Time	Height	Area	Area%
1	13.850	20.767	14.883	360415	19224293	92.989
2	22.533	27.725	23.993	15852	1449476	7.011
				376267	20673769	100.000

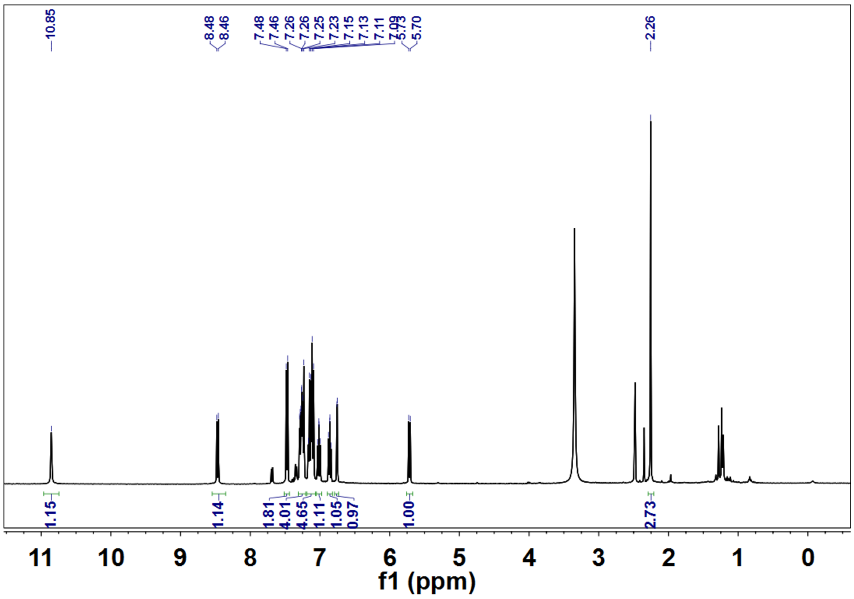

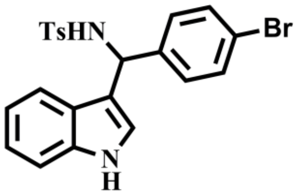

Enantiomeric excess was determined by HPLC with a chiralcel OD-H column (hexane/iPrOH = 70/30, 0.6 mL/min), t_{major} = 18.451 min, t_{minor} = 29.391 min; ee = 97%. ^{1}H NMR (400 MHz, DMSO-d_6) δ 11.01 (s, 1H), 8.68 (d, *J* = 8.6 Hz, 1H), 8.05 (t, *J* = 9.6 Hz, 2H), 7.53 (dd, *J* = 19.0, 8.4 Hz, 4H), 7.32 (d, *J* = 8.4 Hz, 2H), 7.15 (d, *J* = 8.1 Hz, 2H), 7.07 (t, *J* = 7.7 Hz, 1H), 6.92 (t, *J* = 7.6 Hz, 1H), 6.79 (d, *J* = 2.2 Hz, 1H), 5.88 (d, *J* = 8.6 Hz, 1H), 2.27 (s, 3H).

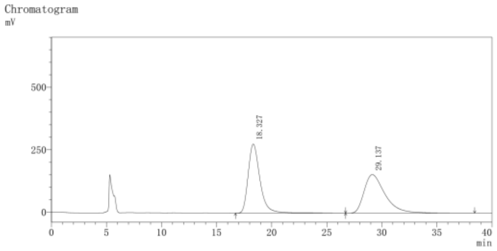

254nm

ID#	Start	End	Ret. Time	Height	Area	Area%
1	16.700	26.717	18.327	276328	20734082	50.431
2	26.717	38.425	29.137	154132	20379608	49.569
				430461	41113690	100.000

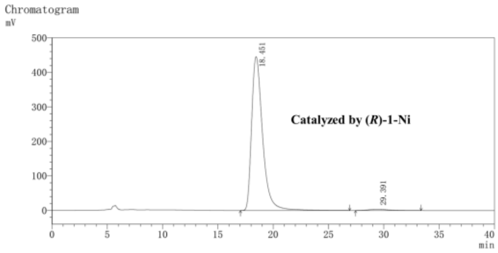

254nm

ID#	Start	End	Ret. Time	Height	Area	Area%
1	17.033	26.908	18.451	446119	30815422	98.671
2	27.442	33.375	29.391	3290	414974	1.329
				449409	31230395	100.000

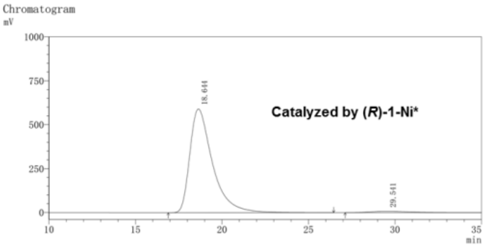

254nm

ID#	Start	End	Ret. Time	Height	Area	Area%
1	16.900	26.458	18.644	590502	53885404	97.674
2	27.117	35.125	29.541	8059	1283454	2.326
				598561	55168858	100.000

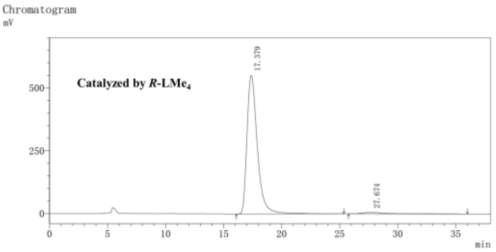

254nm

ID#	Start	End	Ret. Time	Height	Area	Area%
1	16.092	25.358	17.379	552251	34286995	97.613
2	25.758	36.008	27.674	5542	838334	2.387
				557794	35125328	100.000

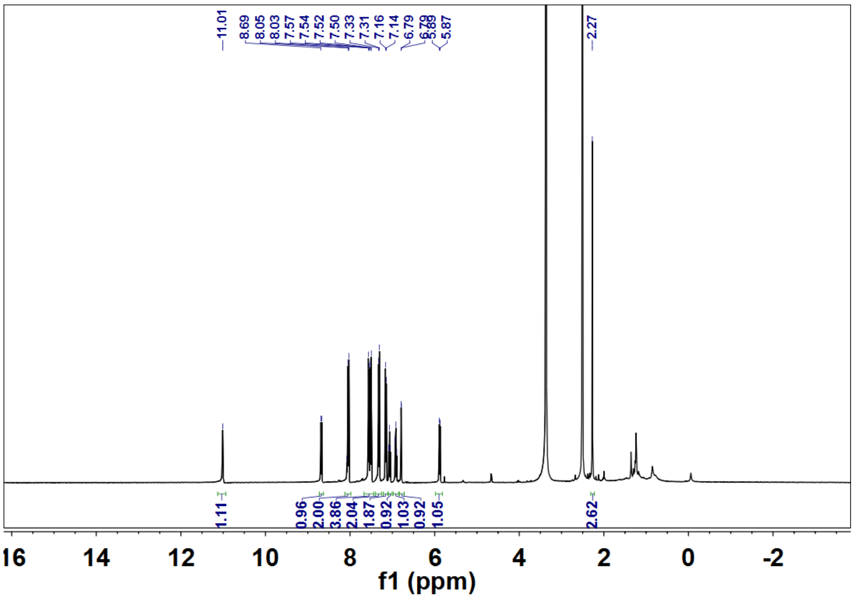

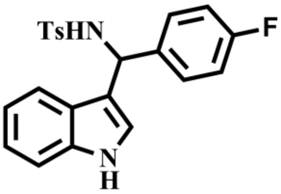

Enantiomeric excess was determined by HPLC with a chiralcel OD-H column (hexane/iPrOH = 70/30, 0.6 mL/min), t_{minor} = 16.614 min, t_{major} = 25.836min; ee = 94%. ^{1}H NMR (400 MHz, DMSO-d_6) δ 10.97 (s, 1H), 8.64 (d, *J* = 8.6 Hz, 1H), 8.09 – 7.89 (m, 2H), 7.52 (ddd, *J* = 18.0, 6.2, 2.1 Hz, 4H), 7.29 (dd, *J* = 8.2, 0.7 Hz, 2H), 7.17 – 7.09 (m, 2H), 7.04 (ddd, *J* = 8.3, 7.1, 1.0 Hz, 1H), 6.89 (ddd, *J* = 7.9, 7.1, 1.0 Hz, 1H), 6.77 (d, *J* = 2.5 Hz, 1H), 5.86 (d, *J* = 8.6 Hz, 1H), 2.25 (s, 3H).

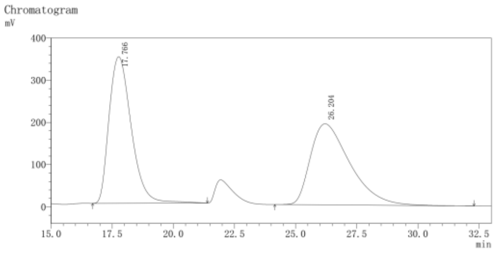

254nm

ID#	Start	End	Ret. Time	Height	Area	Area%
1	16.700	21.392	17.766	347731	21993137	50.383
2	24.150	32.292	26.204	192174	21658731	49.617
				539905	43651868	100.000

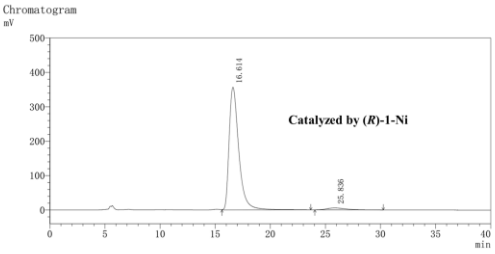

254nm

ID#	Start	End	Ret. Time	Height	Area	Area%
1	15.608	23.675	16.614	356762	21775173	97.085
2	24.042	30.250	25.836	5631	653701	2.915
				362393	22428874	100.000

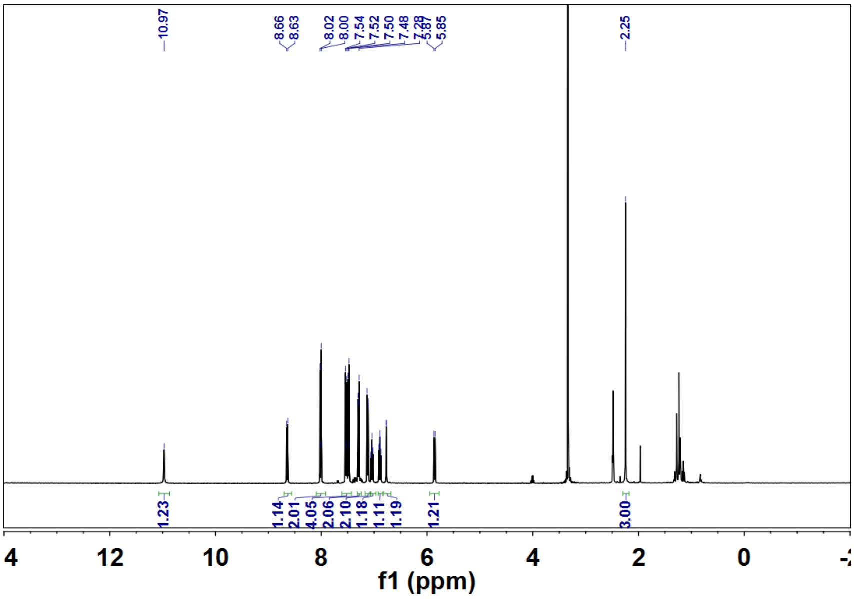

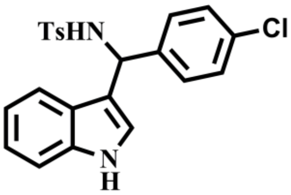

Enantiomeric excess was determined by HPLC with a chiralcel OD-H column (hexane/iPrOH = 70/30, 0.6 mL/min), t_{major} = 16.681 min, t_{minor} = 26.278 min; ee = 98%. ^{1}H NMR (400 MHz, DMSO-d_6) δ 10.94 (s, 1H), 8.54 (d, *J* = 8.9 Hz, 1H), 7.49 (d, *J* = 8.1 Hz, 2H), 7.32 (dd, *J* = 7.9, 4.6 Hz, 2H), 7.27 (d, *J* = 8.5 Hz, 2H), 7.21 (d, *J* = 8.4 Hz, 2H), 7.15 (d, *J* = 8.0 Hz, 2H), 7.06 (t, *J* = 7.6 Hz, 1H), 6.91 (t, *J* = 7.5 Hz, 1H), 6.77 (d, *J* = 2.0 Hz, 1H), 5.81 – 5.69 (m, 1H), 2.31 (s, 3H).

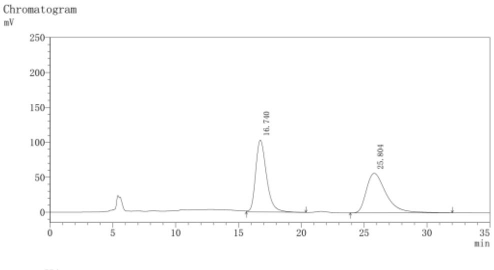

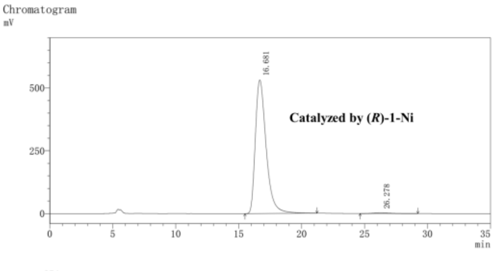

254nm

ID#	Start	End	Ret. Time	Height	Area	Area%
1	15.617	20.367	16.740	102930	6211341	50.260
2	23.900	32.017	25.804	56624	6147083	49.740
				159555	12358425	100.000

254nm

ID#	Start	End	Ret. Time	Height	Area	Area%
1	15.500	21.225	16.681	531778	31722179	99.241
2	24.642	29.242	26.278	2304	242737	0.759
				534083	31964916	100.000

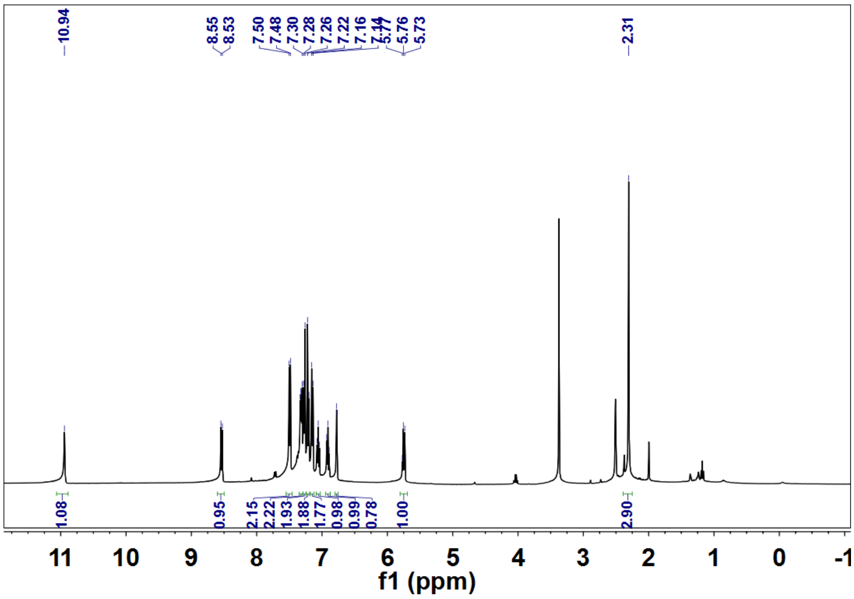

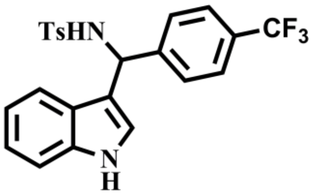

Enantiomeric excess was determined by HPLC with a chiralcel OD-H column (hexane/iPrOH = 70/30, 0.6 mL/min), t_{major} = 16.555 min, t_{minor} = 25.836 min; ee = 99%. ^{1}H NMR (400 MHz, DMSO-d_6) δ 10.92 (s, 1H), 8.57 (d, J = 8.9 Hz, 1H), 7.68 (d, J = 8.2 Hz, 2H), 7.45 (s, 4H), 7.34 (d, J = 7.3 Hz, 3H), 7.30 (d, J = 8.1 Hz, 1H), 7.25 (s, 2H), 7.03 (d, J = 8.0 Hz, 1H), 6.90 (t, J = 7.5 Hz, 1H), 6.75 (d, J = 2.3 Hz, 1H), 5.80 (d, J = 8.9 Hz, 1H), 2.35 (s, 3H).

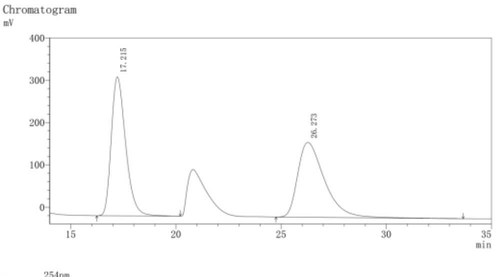

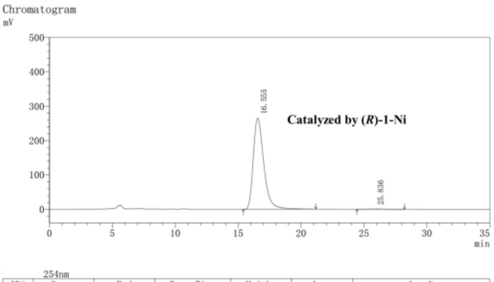

254nm

ID#	Start	End	Ret. Time	Height	Area	Area%
1	16.242	20.208	17.215	328717	15608173	49.796
2	24.758	33.658	26.273	176977	15736093	50.204
				505695	31344267	100.000

254nm

ID#	Start	End	Ret. Time	Height	Area	Area%
1	15.417	21.167	16.555	265261	15468256	99.499
2	24.442	28.217	25.836	811	77871	0.501
				266072	15546128	100.000

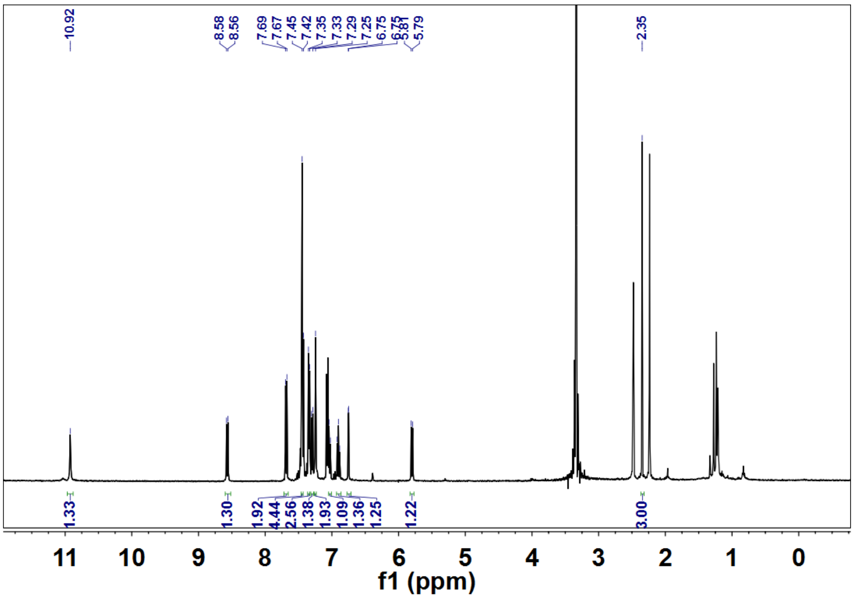

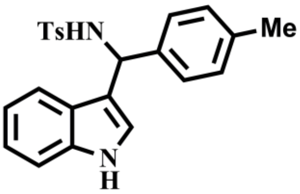

Enantiomeric excess was determined by HPLC with a chiralcel OD-H column (hexane/iPrOH = 70/30, 0.6 mL/min), t_{major} = 13.510 min, t_{minor} = 22.172 min; ee = 90%. ^{1}H NMR (400 MHz, DMSO-d_6) δ 10.84 (s, 1H), 8.40 (d, J = 8.8 Hz, 1H), 7.49 – 7.42 (m, 2H), 7.26 (dd, J = 6.8, 5.8 Hz, 2H), 7.14 – 7.05 (m, 4H), 7.01 (t, J = 7.5 Hz, 1H), 6.94 (d, J = 7.3 Hz, 2H), 6.85 (t, J = 7.6 Hz, 1H), 6.77 (s, 1H), 5.66 (d, J = 8.7 Hz, 1H), 2.26 (s, 3H), 2.19 (s, 3H).

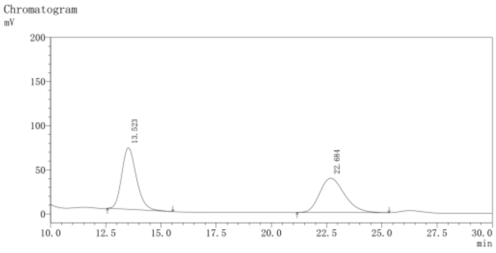

254nm

ID#	Start	End	Ret. Time	Height	Area	Area%
1	12.575	15.542	13.523	69743	3241658	51.677
2	21.167	25.325	22.684	38826	3031259	48.323
				108569	6272918	100.000

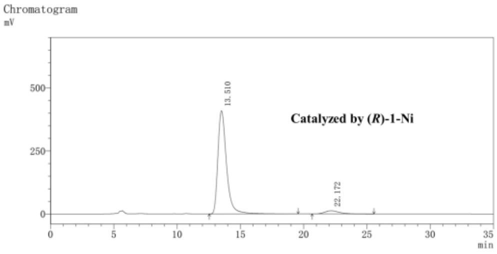

254nm

ID#	Start	End	Ret. Time	Height	Area	Area%
1	12.542	19.567	13.510	409668	19321367	95.140
2	20.675	25.558	22.172	12323	986952	4.860
				421991	20308318	100.000

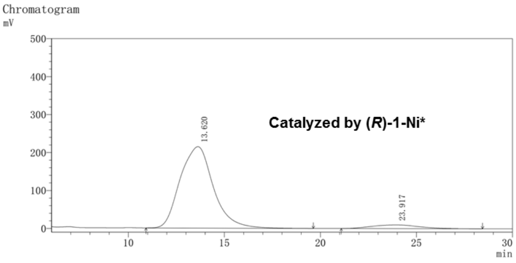

254nm

ID#	Start	End	Ret. Time	Height	Area	Area%
1	10.917	19.633	13.620	214613	25870069	93.945
2	21.083	28.442	23.917	9788	1667397	6.055
				224401	27537466	100.000

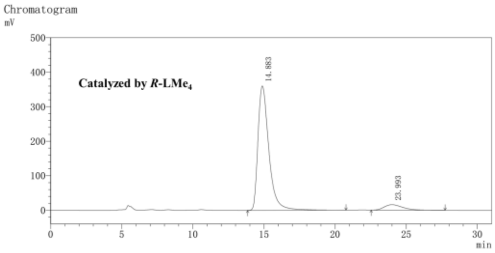

254nm

ID#	Start	End	Ret. Time	Height	Area	Area%
1	13.850	20.767	14.883	360415	19224293	92.989
2	22.533	27.725	23.993	15852	1449476	7.011
				376267	20673769	100.000

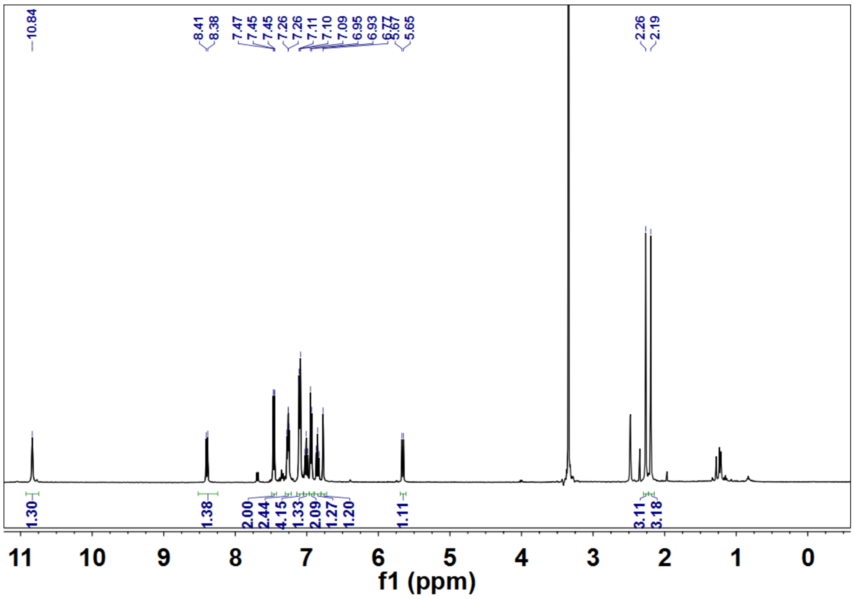

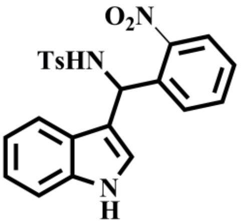

Enantiomeric excess was determined by HPLC with a chiralcel OD-H column (hexane/iPrOH = 70/30, 0.6 mL/min), t_{major} = 16.969 min, t_{minor} = 31.089 min; ee = 97%. ^{1}H NMR (400 MHz, DMSO-d_6) δ 10.99 (s, 1H), 8.68 (d, *J* = 8.2 Hz, 1H), 7.86 – 7.73 (m, 2H), 7.60 (t, *J* = 7.6 Hz, 1H), 7.54 – 7.41 (m, 3H), 7.33 (d, *J* = 8.1 Hz, 1H), 7.22 (d, *J* = 8.1 Hz, 2H), 7.18 – 7.01 (m, 2H), 6.91 (t, *J* = 7.5 Hz, 1H), 6.56 (d, *J* = 2.3 Hz, 1H), 6.39 (d, *J* = 8.1 Hz, 1H), 2.33 (s, 3H).

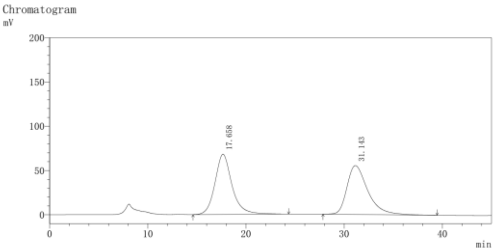

254nm

ID#	Start	End	Ret. Time	Height	Area	Area%
1	14.608	24.350	17.658	68081	8223472	50.232
2	27.842	39.458	31.143	55224	8147489	49.768
				123305	16370961	100.000

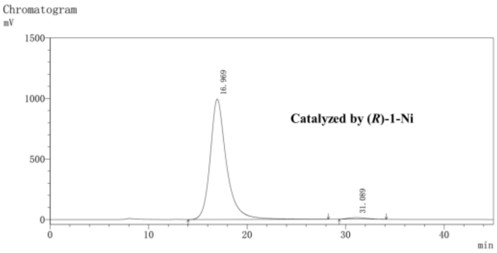

254nm

ID#	Start	End	Ret. Time	Height	Area	Area%
1	14.017	28.242	16.969	993616	114131679	98.552
2	29.350	34.133	31.089	12803	1676658	1.448
				1006418	115808337	100.000

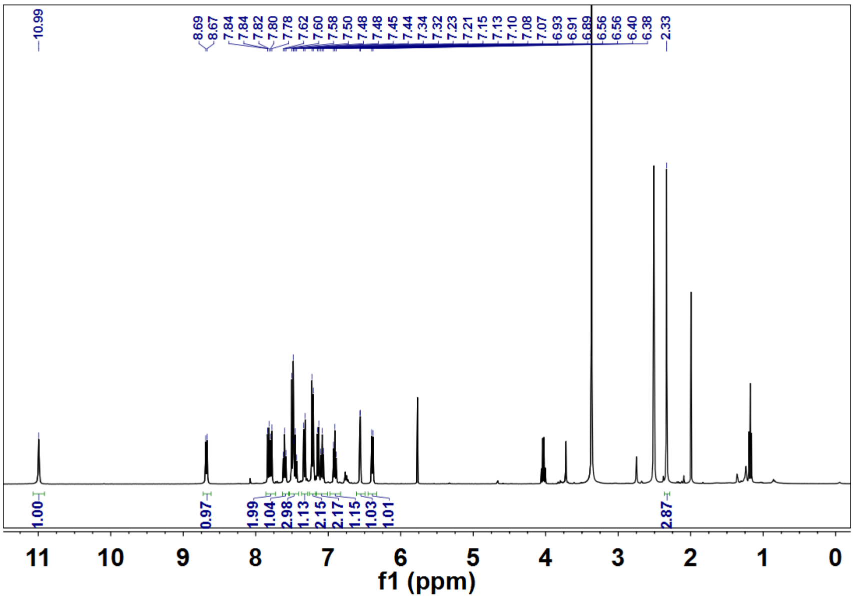

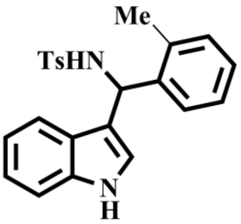

Enantiomeric excess was determined by HPLC with a chiralcel OD-H column (hexane/iPrOH = 70/30, 0.6 mL/min), t_{major} = 9.536 min, t_{minor} = 20.848 min; ee = 99.7%. ^{1}H NMR (400 MHz, DMSO-d_6) δ 10.88 (s, 1H), 8.48 (d, *J* = 8.9 Hz, 1H), 7.69 (d, *J* = 8.2 Hz, 2H), 7.47 (d, *J* = 8.2 Hz, 2H), 7.34 (dd, *J* = 8.0, 0.5 Hz, 3H), 7.12 (d, *J* = 8.4 Hz, 2H), 7.03 (t, *J* = 7.5 Hz, 1H), 6.92 – 6.85 (m, 1H), 6.74 (d, *J* = 2.4 Hz, 1H), 5.73 (d, *J* = 8.9 Hz, 1H), 2.35 (s, 3H), 2.27 (s, 3H).

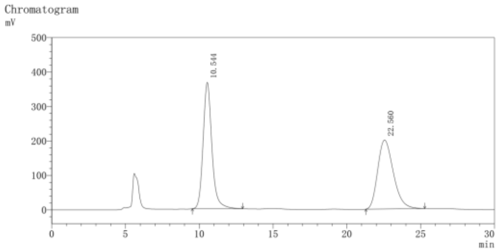

230nm

ID#	Start	End	Ret. Time	Height	Area	Area%
1	9.533	12.933	10.544	366531	15178516	50.664
2	21.292	25.275	22.560	199893	14780376	49.336
				566424	29958892	100.000

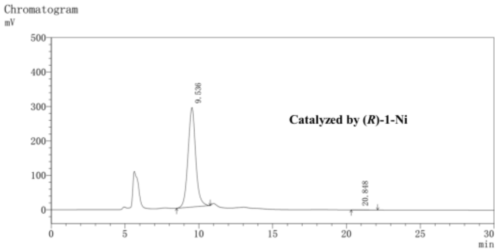

230nm

ID#	Start	End	Ret. Time	Height	Area	Area%
1	8.492	10.775	9.536	288980	10425178	99.849
2	20.317	22.117	20.848	266	15716	0.151
				289246	10440893	100.000

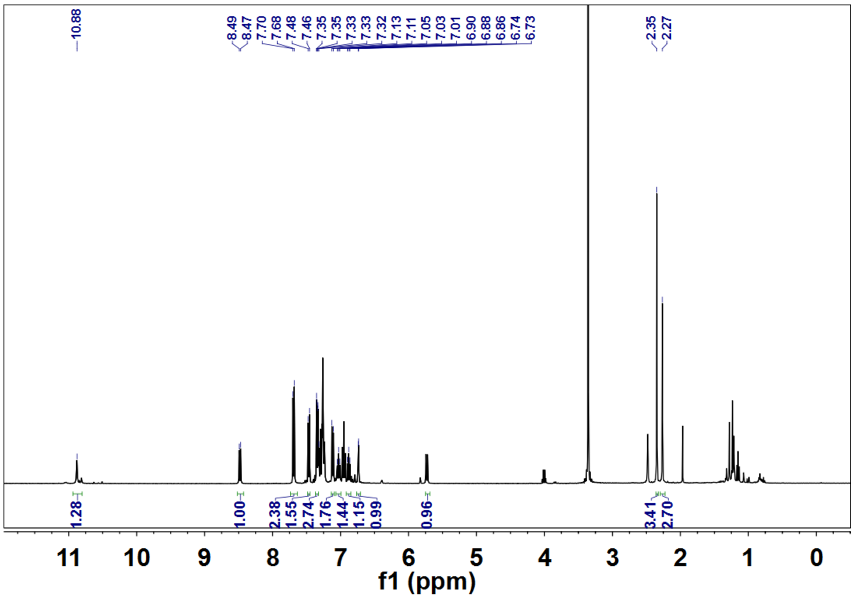

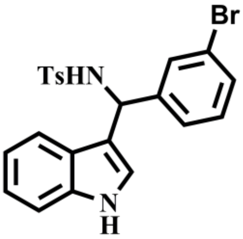

Enantiomeric excess was determined by HPLC with a chiralcel AD-H column (hexane/iPrOH = 80/20, 0.6 mL/min), t_{major} = 45.888 min, t_{minor} = 51.379 min; ee = 99.6%. ^{1}H NMR (400 MHz, DMSO-d_6) δ 10.95 (s, 1H), 8.54 (d, *J* = 9.0 Hz, 1H), 7.50 (d, *J* = 8.1 Hz, 2H), 7.32 (ddd, *J* = 17.7, 12.7, 7.5 Hz, 5H), 7.19 – 7.10 (m, 3H), 7.06 (t, *J* = 7.5 Hz, 1H), 6.92 (t, *J* = 7.4 Hz, 1H), 6.79 (d, *J* = 2.1 Hz, 1H), 5.80 – 5.70 (m, 1H), 2.30 (s, 3H).

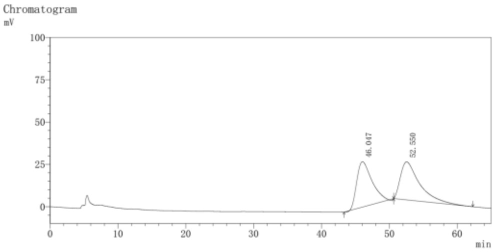

254nm

ID#	Start	End	Ret. Time	Height	Area	Area%
1	43.325	50.642	46.047	26867	4158337	48.381
2	50.642	62.317	52.550	22514	4436597	51.619
				49381	8594935	100.000

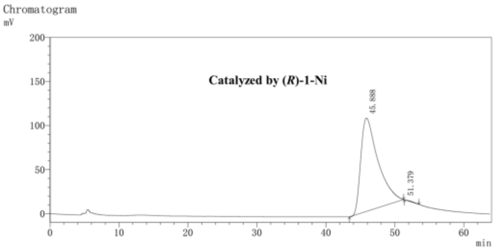

254nm

ID#	Start	End	Ret. Time	Height	Area	Area%
1	43.408	51.275	45.888	105896	18476373	99.782
2	51.375	53.500	51.379	10	40366	0.218
				105907	18516739	100.000

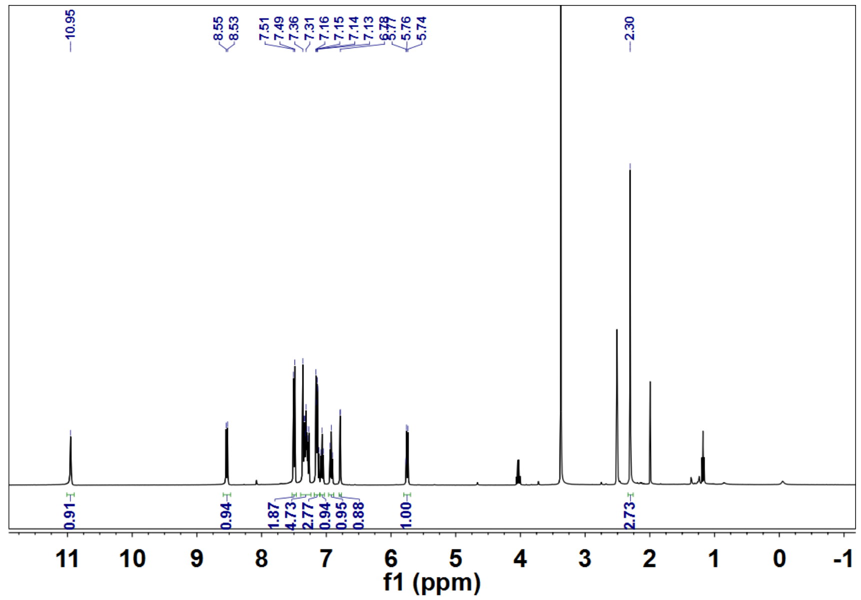
10.95
8.55
8.53
7.51
7.49
7.36
7.31
7.16
7.15
7.14
7.13
5.77
5.76
5.74
2.30
0.91
0.94
1.87
4.73
2.77
0.94
0.95
0.88
1.00
2.73
f1 (ppm)

HPLC copies of recycle experiment for [3+2] coupling reaction

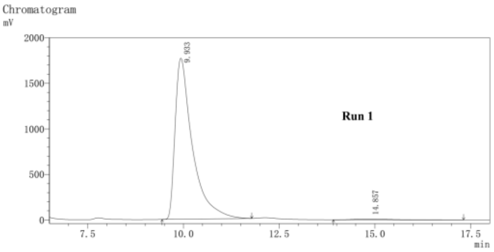

254nm

ID#	Start	End	Ret. Time	Height	Area	Area%
1	9.442	11.783	9.933	1767877	55505677	99.053
2	13.917	17.317	14.857	8037	530427	0.947
				1775913	56036105	100.000

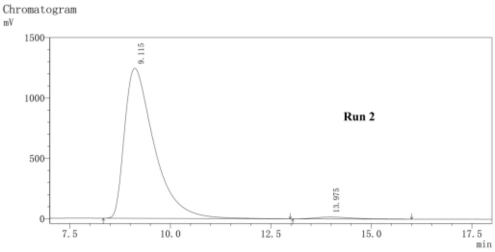

254nm

ID#	Start	End	Ret. Time	Height	Area	Area%
1	8.333	12.983	9.115	1243332	63829400	98.502
2	13.042	16.000	13.975	15738	970814	1.498
				1259070	64800214	100.000

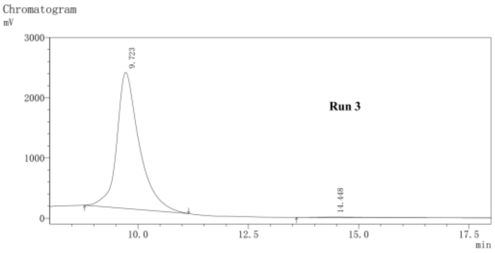

254nm

ID#	Start	End	Ret. Time	Height	Area	Area%
1	8.792	11.150	9.723	2261965	80040574	99.215
2	13.592	18.708	14.448	7684	633664	0.785
				2269649	80674238	100.000

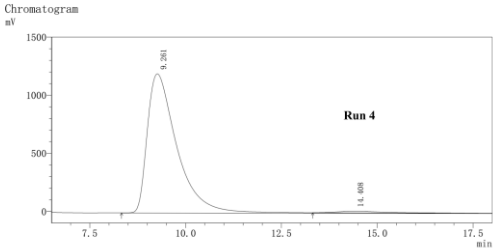

254nm

ID#	Start	End	Ret. Time	Height	Area	Area%
1	8.325	18.933	9.261	1198191	65405348	97.939
2	13.317	18.508	14.408	14356	1376414	2.061
				1212547	66781762	100.000

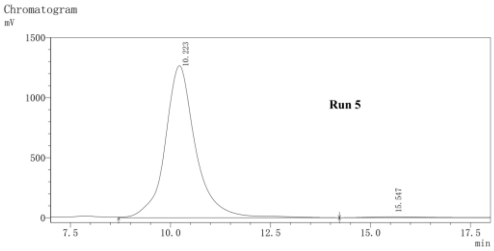

254nm

ID#	Start	End	Ret. Time	Height	Area	Area%
1	8.700	14.225	10.223	1265488	66955546	98.971
2	14.225	19.217	15.547	7197	696049	1.029
				1272684	67651595	100.000

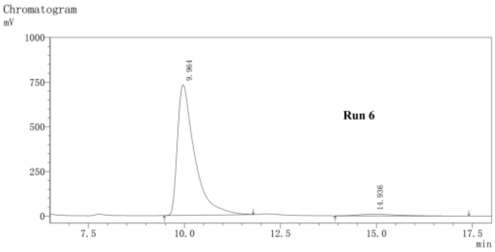

254nm

ID#	Start	End	Ret. Time	Height	Area	Area%
1	9.475	11.792	9.964	731729	22777899	97.382
2	13.925	17.408	14.936	9026	612402	2.618
				740755	23390301	100.000

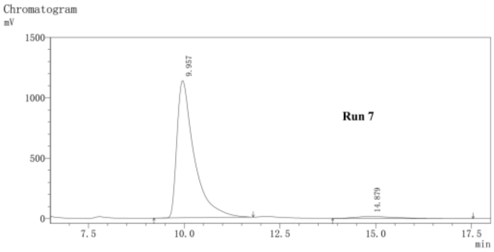

254nm

ID#	Start	End	Ret. Time	Height	Area	Area%
1	9.208	11.800	9.957	1136693	35648689	97.194
2	13.875	17.542	14.879	15129	1029093	2.806
				1151822	36677782	100.000

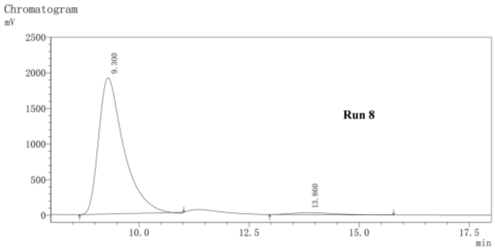

254nm

ID#	Start	End	Ret. Time	Height	Area	Area%
1	8.650	11.017	9.300	1912632	75689198	97.730
2	12.967	15.783	13.860	26865	1757841	2.270
				1939497	77447039	100.000

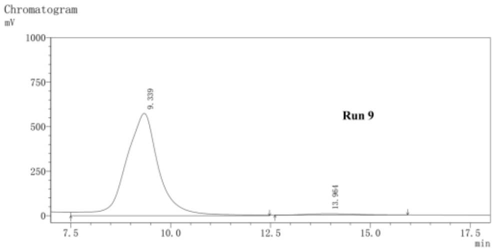

254nm

ID#	Start	End	Ret. Time	Height	Area	Area%
1	7.500	12.475	9.339	575000	32946874	98.152
2	12.608	15.933	13.964	7357	620370	1.848
				582357	33567244	100.000

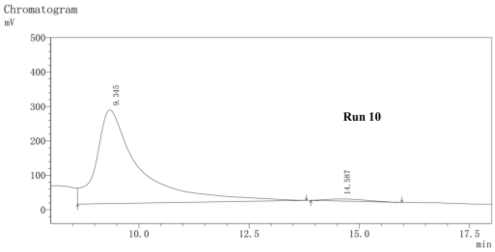

254nm

ID#	Start	End	Ret. Time	Height	Area	Area%
1	8.608	13.800	9.345	271737	18586637	97.989
2	13.900	15.967	14.587	5997	381539	2.011
				277734	18968176	100.000

HPLC copies of recycle experiment for Friedel-Crafts alkylation

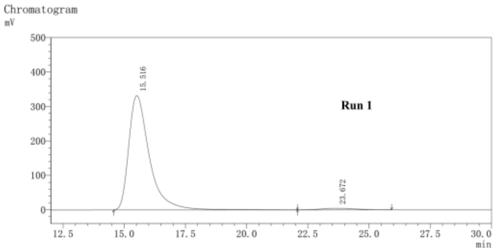

ID#	Start	End	Ret. Time	Height	Area	Area%
1	14.567	22.075	15.516	331922	19364614	97.771
2	22.075	25.925	23.672	4608	441389	2.229
				336530	19806003	100.000

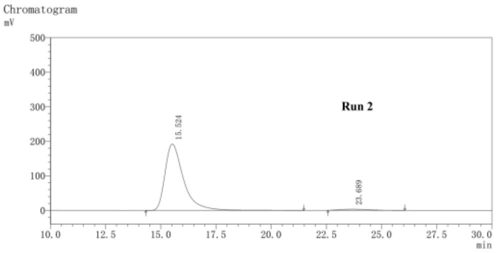

ID#	Start	End	Ret. Time	Height	Area	Area%
1	14.325	21.483	15.524	192284	11280067	97.428
2	22.575	26.050	23.689	3309	297829	2.572
				195593	11577896	100.000

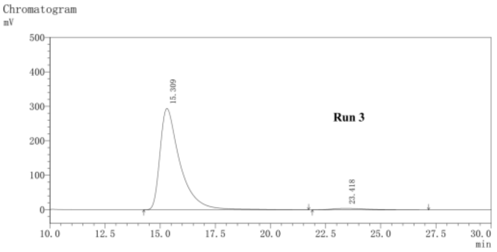

254nm

ID#	Start	End	Ret. Time	Height	Area	Area%
1	14.258	21.733	15.309	294198	18543783	97.752
2	21.900	27.167	23.418	4126	426366	2.248
				298324	18970149	100.000

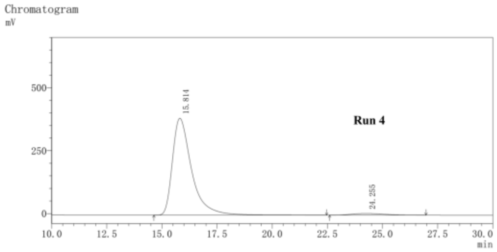

254nm

ID#	Start	End	Ret. Time	Height	Area	Area%
1	14.633	22.467	15.814	384997	23287528	97.185
2	22.600	26.975	24.255	6562	674651	2.815
				391559	23962180	100.000

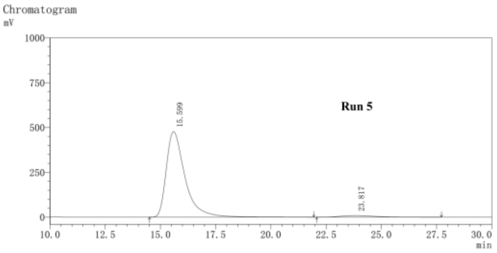

ID#	Start	End	Ret. Time	Height	Area	Area%
1	14.508	21.950	15.599	477319	28136168	96.833
2	22.067	27.725	23.817	8824	920129	3.167
				486143	29056297	100.000

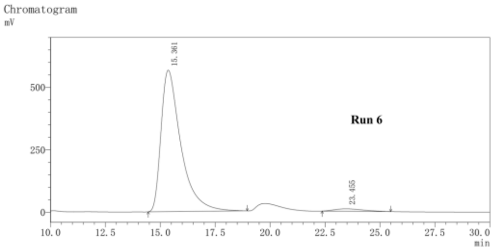

254nm

ID#	Start	End	Ret. Time	Height	Area	Area%
1	14.450	18.958	15.361	566090	35524881	97.610
2	22.367	25.492	23.455	9661	869855	2.390
				575751	36394736	100.000

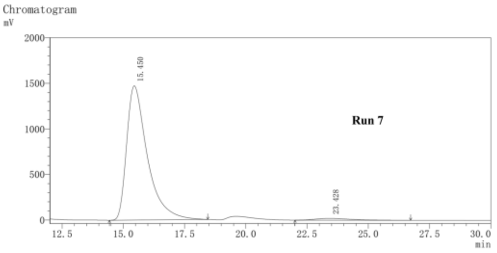

254nm

ID#	Start	End	Ret. Time	Height	Area	Area%
1	14.433	18.450	15.450	1472931	86578660	97.830
2	22.008	26.725	23.428	19406	1920153	2.170
				1492337	88498813	100.000

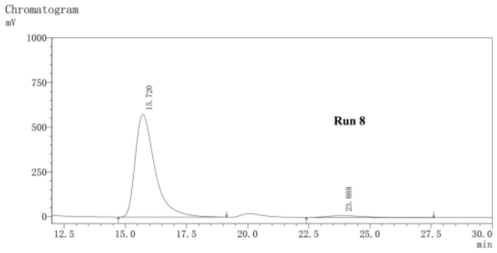

ID#	Start	End	Ret. Time	Height	Area	Area%
1	14.717	19.142	15.720	573879	32177723	96.886
2	22.400	27.608	23.888	10624	1034083	3.114
				584503	33211806	100.000

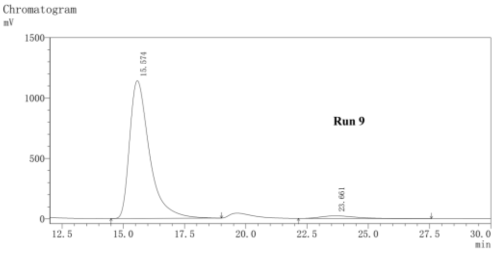

254nm

ID#	Start	End	Ret. Time	Height	Area	Area%
1	14.500	19.008	15.574	1139709	64116479	96.766
2	22.150	27.575	23.661	21697	2142952	3.234
				1161406	66259431	100.000

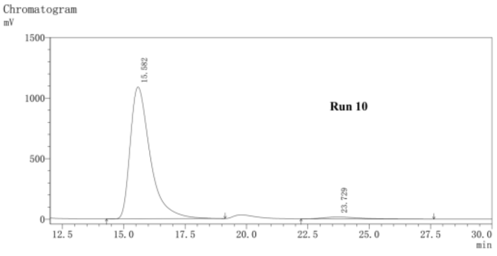

ID#	Start	End	Ret. Time	Height	Area	Area%
1	14.300	19.133	15.582	1088149	61649494	97.527
2	22.225	27.633	23.729	15796	1563097	2.473
				1103944	63212591	100.000

HPLC copies of [3+2] coupling reaction at different catalyst loadings

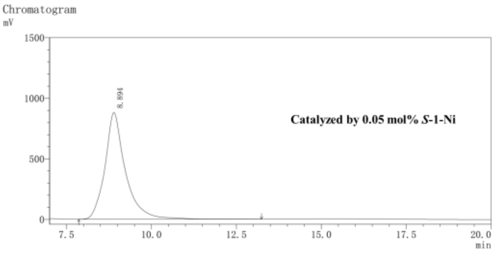

254nm

ID#	Start	End	Ret. Time	Height	Area	Area%
1	7.858	13.242	8.894	880881	36921182	100.000
				880881	36921182	100.000

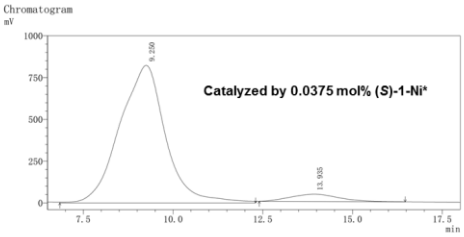

230nm

ID#	Start	End	Ret. Time	Height	Area	Area%
1	6.842	12.300	9.250	823477	73335366	94.773
2	12.400	16.467	13.935	42879	4044297	5.227
				866355	77379663	100.000

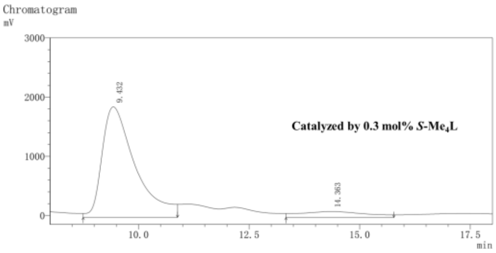

230nm

ID#	Start	End	Ret. Time	Height	Area	Area%
1	8.750	10.883	9.432	1865325	97305581	90.010
2	13.333	15.775	14.363	100583	10799612	9.990
				1965908	108105192	100.000

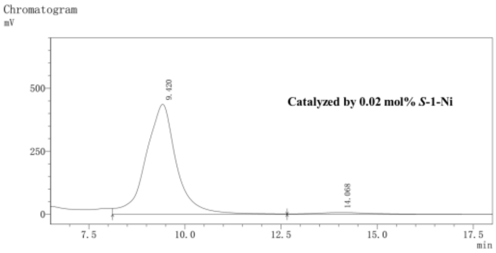

ID#	Start	End	Ret. Time	Height	Area	Area%
1	8.117	12.658	9.420	436628	24223170	97.054
2	12.658	18.500	14.068	6897	735231	2.946
				443525	24958401	100.000

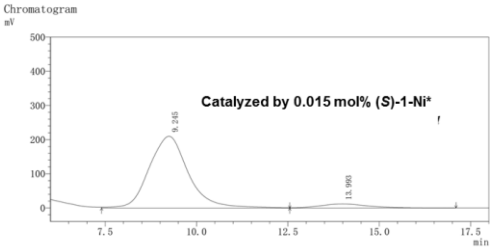

254nm

ID#	Start	End	Ret. Time	Height	Area	Area%
1	7.400	12.550	9.245	210108	15639189	93.562
2	12.550	17.100	13.993	11752	1076220	6.438
				221860	16715410	100.000

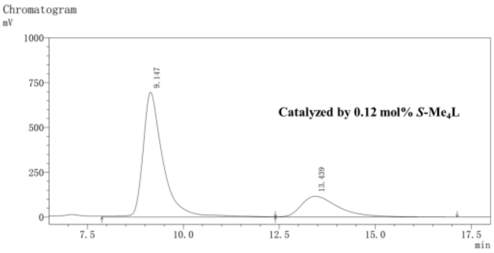

ID#	Start	End	Ret. Time	Height	Area	Area%
1	7.875	12.400	9.147	695922	25200505	76.462
2	12.400	17.133	13.439	115792	7757827	23.538
				811714	32958332	100.000

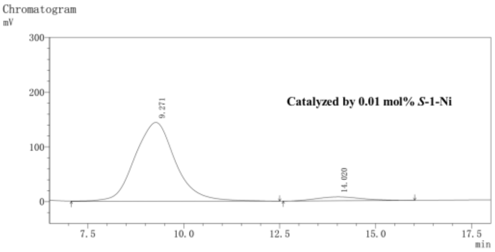

ID#	Start	End	Ret. Time	Height	Area	Area%
1	7.067	12.500	9.271	144143	10643279	94.738
2	12.592	16.017	14.020	7138	591194	5.262
				151280	11234473	100.000

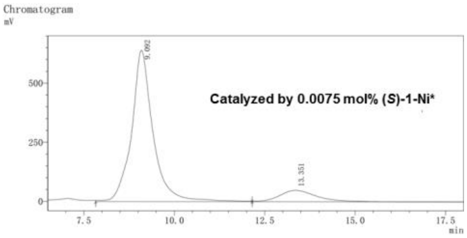

254nm

ID#	Start	End	Ret. Time	Height	Area	Area%
1	7.825	12.150	9.092	640640	26618688	88.210
2	12.150	19.275	13.351	48832	3557928	11.790
				689472	30176615	100.000

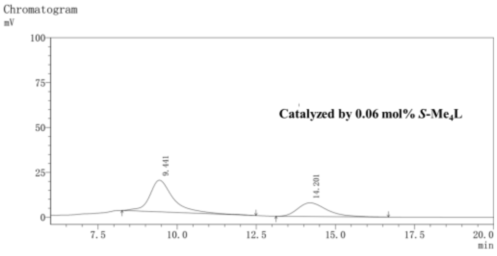

ID#	Start	End	Ret. Time	Height	Area	Area%
1	8.258	12.492	9.441	17647	935330	65.769
2	13.125	16.675	14.201	7602	486804	34.231
				25250	1422133	100.000

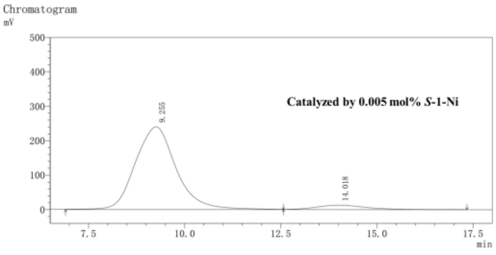

ID#	Start	End	Ret. Time	Height	Area	Area%
1	6.900	12.567	9.255	240645	17906667	93.744
2	12.567	17.342	14.018	13046	1195041	6.256
				253690	19101709	100.000

Chromatogram

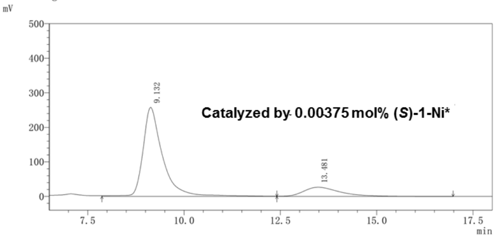

254nm

ID#	Start	End	Ret. Time	Height	Area	Area%
1	7.867	12.408	9.132	257857	9179351	83.746
2	12.408	16.983	13.481	26740	1781543	16.254
				284597	10960895	100.000

Supplementary References

(1) V. B. Birman., A. L. Rheingold. & K. Lam. 1,1′-Spirobiindane-7,7′-diol: a novel, *C*2-symmetric chiral ligand. *Tetrahedron: Asymmetry*, **10**, 125-131 (1999).

(2) Zhang, J. *et al.* Highly efficient and practical resolution of 1,1′-spirobiindane-7,7′-diol by inclusion crystallization with N-benzylcinchonidinium chloride. *Tetrahedron: Asymmetry*, **13**, 1363-1366 (2002).

(3) Shu, C. *et al.* Lewis Acid Catalyzed [3+2] Coupling of Indoles with Quinone Monoacetals or Quinone Imine Ketal. *Eur. J. Org. Chem.*, 4467-4471 (2014).

(4) Liao, L. *et al.* Highly Enantioselective [3+2] Coupling of Indoles with Quinone Monoimines Promoted by a Chiral Phosphoric Acid. *Angew. Chem. Int. Ed.*, **53**, 10471-10475 (2014).

(5) Cheng, H. *et al.* Highly Enantioselective Friedel–Crafts Alkylation/N‐Hemiacetalization Cascade Reaction with Indoles. *Angew. Chem. Int. Ed.*, **52**, 3250-3254 (2013).

(6) Tu, D. *et al.* Palladium-catalysed direct C-2 methylation of indoles. *Organic & Biomolecular Chemistry*, **14**, 7443-7446 (2016).

(7) Angelovski, G. *et al.* A Rapid and Reliable Assay for Regioselectivity Using Fluorescence Spectroscopy. *Adv. Synth. Catal.*, **348**, 1193-1199 (2006).

(8) Yang, Z. *et al.* In situ generated cationic Pd(II)/bipyridine-catalyzed addition of arylboronic acids to N-sulfonyl-arylaldimines. *Tetrahedron Letters*, **58**, 2034-2037 (2017).

(9) Yu. P., He. J. & Guo. C. 9-Thiourea Cinchona alkaloid supported on mesoporous silica as a highly enantioselective, recyclable heterogeneous asymmetric catalyst. *Chem. Commun.*, 2355-2357 (2008).

(10) Planes, L. O., Escrich, C. R. & Pericàs, M. A. Enantioselective Continuous‐Flow Production of 3-Indolylmethanamines Mediated by an Immobilized Phosphoric Acid Catalyst. *Chem. Eur. J.*, **20**, 2367 (2014).

(11) Xing. C. *et al.* Optically Active 1,1′-Spirobiindane-7,7′-diol (SPINOL)-Based Phosphoric Acids as Highly Enantioselective Catalysts for Asymmetric Organocatalysis. *J. Org. Chem.*, **76**, 4125-4131 (2011).

(12) Kang. Q., Zhao. Z. & You. S. Highly Enantioselective Friedel−Crafts Reaction of Indoles with Imines by a Chiral Phosphoric Acid. *J. Am. Chem. Soc.*, **129**, 1484-1485 (2007).

(13) Chen. L. *et al.* Chiral Sulfonimide as a Brønsted Acid Organocatalyst for Asymmetric Friedel–Crafts Alkylation of Indoles with Imines. *J. Org. Chem.*, **76**, 7141-7147 (2011).

(14) Jia. Y. *et al.* Asymmetric Friedel−Crafts Addition of Indoles to N-Sulfonyl Aldimines: A Simple Approach to Optically Active 3-Indolyl-methanamine Derivatives. *Org. Lett.*, **8**, 1621-1624 (2006).

(15) Kumari. P. *et al.* Asymmetric Friedel–Crafts addition of indoles to N-sulfonyl aldimines catalyzed by Cu(II) chiral amino alcohol based Schiff base complexes. *Catal. Sci. Technol.*, **4**, 563 (2014).
